# HIV risk perception, trust and PrEP adherence among participants in an HIV prevention trial: a qualitative longitudinal study, South Africa

**DOI:** 10.1136/bmjopen-2024-086742

**Published:** 2025-04-23

**Authors:** Rujeko Samanthia Chimukuche, Londiwe Shandu, Silindile Zulu, Phindile Khanyile, Nishanta Singh, Zakir Gaffoor, Rachel Kawuma, Sheena McCormack, Janet Seeley, on behalf of the PrEPVacc Study Group, Glenda Gray

**Affiliations:** 1 Africa Health Research Institute, Durban, KwaZulu-Natal, South Africa; 2 Division of Infection & Immunity, University College London, London, UK; 3 HIV and Other Infectious Diseases Research Unit, South African Medical Research Council, Cape Town, South Africa; 4 Social Science, MRC/UVRI and LSHTM Uganda Research Unit, Entebbe, Uganda; 5 MRC Clinical Trials Unit, University College London, London, UK; 6 Department of Global Health and Development, London School of Hygiene and Tropical Medicine, London, UK

**Keywords:** HIV & AIDS, QUALITATIVE RESEARCH, Health, Public health

## Abstract

**Abstract:**

**Introduction:**

Ensuring the effectiveness of HIV prevention and treatment methods requires high levels of adherence. Studies have recognised the significance of trust in shaping HIV risk perception.

**Aim:**

In this qualitative analysis, our aim was to explore risk perceptions and understand how individuals assess and respond to HIV risks, as well as their uptake and adherence to oral pre-exposure prophylaxis (PrEP).

**Setting:**

The study was based on the setting of an HIV prevention trial conducted in South Africa.

**Methods:**

Thirty individuals, 9% of the total clinical trial participants, enrolled in the clinical trial were purposively selected and interviewed at three time points within the trial during the follow-up phase. Data analysis was conducted using the Trust, Confidence and Cooperation framework that included constructs of trust, confidence and cooperation.

**Results:**

The findings show that the ongoing participation in the clinical trial played a significant role in influencing participants’ decision to continue PrEP as HIV prevention. This decision was grounded in their trust that PrEP would effectively reduce their vulnerability to HIV and infection.

**Conclusion:**

Clear and consistent health-promoting initiatives enhance participants’ self-awareness of HIV risks and promote understanding (uptake) and effectiveness of HIV prevention methods.

**Trial registration number:**

NCT04066881.

**STRENGTHS AND LIMITATIONS OF THIS STUDY**

- The study used a longitudinal approach to assess participants’ views over time.
- The study used the Trust, Confidence, and Cooperation (TCC) framework.
- The case study approach explored each individual’s experiences in detail.
- The sample was limited to participants in a clinical trial population and not generalisable to the broader population.
- HIV risk perception was based on the TCC framework which is mainly cognitive.

## Introduction

 In 2015, the WHO included daily oral pre-exposure prophylaxis (PrEP) as an additional prevention choice for those at substantial risk of HIV infection.^1^ In 2019, WHO updated the recommendation to include an event-based regimen for men who have sex with men.^2 3^ Since then, oral PrEP has become increasingly available as a daily regimen.^4^ In 2021, HIV prevention studies and clinical trial global ethical guidelines recommended that investigators and sponsors of HIV prevention studies adhere to baseline requirements of offering the WHO-recommended package of HIV prevention strategies that includes the provision of PrEP for individuals facing a significant risk of contracting HIV.^5^ In South Africa, the focus of this paper, PrEP has been incorporated into national HIV prevention policies and programmes.^6^

Effectiveness in preventing HIV requires good adherence to HIV prevention and treatment methods. Research has identified the importance of trust in risk perception and the acceptance of new ideas or interventions. Trust can be considered an important factor in explaining acceptance or non-use of HIV prevention interventions in settings such as clinical trials, because when people have little knowledge about an issue that is important to them, motivations and confidence to use and adhere to prevention are based on trust.^7 8^ HIV risk perception is related to the level of trust a person has in the behaviour of their sexual partner,^9^ which influences whether someone believes they need to take preventive measures to maintain their HIV-negative status.^10^ This perception therefore correlates with the willingness to use PrEP, which is key in PrEP uptake and adherence.^11^

Using the Trust, Confidence, and Cooperation (TCC) model, we assessed HIV risk perception as an approach to understanding how individuals evaluate and respond to HIV risks,^12^ including their uptake of PrEP. Studies have shown that assessing the trustworthiness of information sources is vital in risk perception as was observed during the COVID-19 pandemic.^13 14^ The TCC model can be used to explain how trust plays an important role in shaping people’s risk perceptions and responses towards risks.^8^

In this analysis, we used the TCC framework to understand how individuals perceived their risk of HIV infection as it relates to their willingness to use and adhere to PrEP. We further assessed how individuals may perceive health risks and make decisions regarding preventive actions such as using PrEP for HIV prevention.

### Theoretical framework

The TCC framework consists of three core concepts, trust, confidence and cooperation, which are central to understanding and encouraging positive relationships, and interactions within various contexts. Trust is the foundation of the framework and involves the belief or confidence that one party has in the reliability, honesty and competence of another party.^15^ Trust is the willingness to make oneself vulnerable believing in the integrity of another while expecting beneficial outcomes.^15^ It can be interpersonal trust or lack thereof between two individuals or institutional trust where individuals place confidence in an institution.^16^ Trust can be influenced by factors such as past experiences, perceived reliability and the belief that the other party will act in a way that is beneficial or at least not harmful. In this analysis, the concept of trust is used to understand HIV risk perception and the positive changes in health behaviours, including uptake and continued use of PrEP.

Confidence is closely related to trust but focuses on one’s self-assurance and belief in their own abilities or in the abilities of their team or organisation.^8^ When individuals have confidence, they are less likely to perceive themselves at risk. In this analysis, the concept of confidence was used to explain the decreased perception of HIV risk as participants progressed in the clinical trial because of their ongoing participation in the trial and uptake and adherence to PrEP.

Cooperation involves the act of working together with others towards common goals or objectives. It requires mutual effort, coordination and collaboration. Effective cooperation can lead to increased efficiency and improved outcomes. Trust and confidence are often prerequisites for cooperation, as individuals or groups are more likely to engage in cooperative behaviour when they trust and have confidence in the process. This concept was used to understand the gratitude expressed by the clinical trial participants for being part of a study that positively influenced their health behaviours.

## Methods

### Study design and setting

This qualitative methods study was nested within the PrEPVacc trial (NCT04066881). The PrEPVacc trial was conducted at a clinical research site (CRS) located in Durban, South Africa, an area with high HIV prevalence. KwaZulu-Natal Province bears the greatest HIV burden in South Africa, with 60% of this burden concentrated in the eThekwini municipality, centred around Durban.^17^ The PrEPVacc trial is a phase IIb three-arm, two-stage HIV prophylactic vaccine trial with a second randomisation to compare tenofovir alafenamide (TAF) plus emtricitabine (FTC) (TAF/FTC) with tenofovir desoproxil fumarate/emtricitabine (TDF/FTC) as PrEP.^18^ The objectives of this phase IIb clinical trial were: (1) to determine the vaccine efficacy of each combination HIV vaccine regimen compared with placebo, in preventing the acquisition of HIV infection; (2) to determine the effectiveness of TAF/FTC PrEP as a function of the effectiveness of TDF/FTC PrEP and hypothetical placebo; (3) to determine the safety of (i) each combination HIV vaccine regimen compared with placebo and (ii) TAF/FTC PrEP in comparison with TDF/FTC PrEP and (4) to understand the trial results in the context of risk and adherence behaviours, knowledge attitudes, and perceptions of participants, study staff and the wider community (the trial protocol can be found at online supplemental file 1). Ethical approval for the study was obtained from the South Africa Human Research Ethics Committee (protocol ID EC020-11/2019).

In the randomised vaccine trial, each participant was scheduled to receive injections of either vaccine A or B or a saline placebo over a 48-week schedule at four separate time points. In the second randomisation, oral PrEP was provided as a study drug to be taken daily until 2 weeks after the third vaccination.^19^ Thereafter, participants who chose to continue with oral PrEP were offered locally approved PrEP (TDF/FTC) at the trial site as a ‘non-study’ drug.

### Patient and public involvement

Patients or the public were not involved in the design, or conduct or reporting of our research. For dissemination, study public engagement staff have worked closely with Community Advisory Board members and other local leaders to plan and share findings with participants and study communities.

### Participants, sample selection and data collection

Individuals who were HIV negative (18–40 years) and considered to be at high risk of HIV acquisition as per the baseline risk assessment criteria were recruited to join the PrEPVacc study. Every participant was encouraged to take oral study PrEP once they were enrolled in the study.

Thirty participants, 9% of the total enrolled in Durban, were purposively selected to be representative of those randomised to TAF/ FTC (Descovy) or TDF/FTC (Truvada), age groups (18–40 years), gender and socioeconomic characteristics. The choice of sample size was based on our experience and that of others^20^ to enable us to gather sufficient data on our areas of focus. The type of oral PrEP given was known to participants in the clinical trial (participants were not blinded to the type of PrEP).

We conducted longitudinal in-depth interviews (IDIs) between February 2022 and October 2023 (online supplemental file 2). IDIs were conducted with the same participants in three phases throughout the study. Twenty-five participants completed three interviews assessing PrEP adherence and facilitators/barriers to persistence. The first interviews were conducted after 2 months of participation in the clinical trial. The second interviews occurred 4 months after the week 26 visit within the PrEP study period and the third interviews occurred in the non-study PrEP period, after 1 year of participation in the clinical trial. The interviews were done longitudinally to assess whether participants continued with PrEP, their HIV risk perception, the facilitators and barriers to PrEP uptake when offered as a non-study drug, and their experiences with the vaccine and clinical trial in general.

A semistructured topic guide was used to guide the interview and participant responses to questions on HIV risk perceptions before enrolling in the clinical trial and at the time the interview was conducted. Three female social science researchers conducted the IDIs in IsiZulu (the main local language that participants were most comfortable with) face-to-face at the CRS. Each interview lasted between 45 and 60 min. Informed consent was sought from each participant after the purpose of the substudy was explained. Participants were also given time to review the participant information documentation prior to giving their written informed consent to be engaged in the interview process. Participants were not involved in the design, conduct, reporting or dissemination plans of our research.

Data were digitally recorded, transcribed and translated into English and stored on a password-protected data server. Interview summaries were written by the social science researchers immediately after each interview. These summaries provided an overview of the conduct of the interview and the main points raised in order to complement the transcription after processing. Debriefings were conducted after each interview between the three interviewers and the lead author of this study to agree on probes for future interviews, provide clarity on emerging themes and refine the topic guides where necessary. These debriefs were beneficial for the analysis and interpretation of the data.

### Data analysis and interpretation

Data analysis was done manually, led by the first author (RSC), and the research assistants (LS, PK and SZ) who collected the data. The coding framework was developed by RSC using the TCC model and from the data that the entire team reviewed and used to identify emerging codes. The main codes were reviewed, discussed and agreed on by consensus for validity, and similar codes were merged to avoid duplication. In this study, major themes were identified using the theoretical framework: trust, confidence and cooperation. Thereafter, for this analysis, a matrix coding framework was developed in MS Excel on which coded data were manually inserted by pasting information and illustrative quotes from the interviews against the matching themes. All data were anonymised, and participants were identified only by their sex, age and interview round in this study.

## Results

A total of 30 participants, 22 females and 8 males, were recruited and interviewed during the first interviews. Ten participants from the sample indicated that they were employed or self-employed. Four participants stated that they were single with no partners, and 26 indicated that they were in a relationship. Five participants were lost to follow-up on the second and third interviews. We present the findings against each theme derived from the TCC framework below using illustrative quotes.

### Trust

Our results have shown that participants’ trust in the clinical trial was shaped by their past experiences of engaging in high-risk behaviours with multiple partners, having condomless sex with partners whose HIV status they did not know and a lack of trust in their partners. This motivated them to engage in the PrEPVacc trial and trust that the information they received regarding HIV prevention methods and that oral PrEP would protect them from infection.

In the early stages of the clinical trial, we used the first IDI with each person to understand their perception of risk prior to enrollment in the study. Participants indicated that they perceived HIV as a risk or threat before enrolling in the clinical trial with great fear of acquiring HIV. The results from interview one show that participants engaged in high-HIV-risk behaviours that included having multiple partners and engaging in condomless sex with multiple partners, many of whom did not know their HIV status. “Yes, I was at risk… It is having many girlfriends and I have fun without a condom. You see…” Male, young adult (IDI 1). Similarly, another participant stated, “I would say cheating, cheating for the mother of my child without using a condom. I would see that I can be infected at any time.” Male, young adult (IDI 1). Females, on the contrary, expressed that they felt at risk because of their sexual partners, “You know partners, they love women. I would be at risk of being infected…” Female, young adult (IDI 1). Other participants expressed a lack of trust, especially when engaging with multiple partners:

Ey, I don’t know. I don’t know because my partners are HIV negative although I don’t go with them on their daily activities. I cannot be sure about it. I can say that I trust them only to find that I don’t know what they do in my absentia, and claim that I don’t trust them, yet they behave, you see… Female, adult (IDI 2)

The decision to take PrEP and adhere to the daily regimen was seen as a preventive action based on the trust that it will reduce their susceptibility to HIV infection:

You cannot just enrol in this study just because you have seen your friend do so but, you agree to participate because you know that you may be at risk of HIV being transmitted to you and so you agree to use measures to prevent that from happening, you want to protect yourself from entering in such a situation… Female, young adult (IDI 1)

Others trusted PrEP, expressing confidence that they were not susceptible to HIV risk:

I think I am not at risk because I believe the pills that they are giving us are for the prevention of HIV. Female, adult (IDI 1)

Other participants perceived themselves as being at high risk, particularly when engaging in condomless sex with a partner who had not undergone HIV testing:

Before, I don’t want to lie, I used to see myself at risk, I’ll be honest with you. It happened especially when you were with your partner and had sex without protection; men in most of the times they don’t want to or they’re lazy to do HIV testing… So, I used to see myself at a huge risk. Female, young adult (IDI 2)

Some participants perceived themselves to be at risk of HIV infection because they lacked trust in their partners. They believed that their partners were engaging in risky behaviours in their absence, “Mh, I can say that I was at risk because I do not know the status of the person and I will never know because sometimes even if I ask, he will tell me something else that is not true…” Female, late adolescent (IDI 1).

The participants’ confidence in the health services that were provided through the clinical trial and the provision of PrEP as an intervention significantly influenced their perception of low HIV risk.

It was because, every time I always check my blood, but I thought I will get to the point where I get scared to. What I like here is that… what actually brought me here was I heard that there is PrEP, the pills to prevent HIV infection, that is what motivated me to come here. Also, that we check blood whenever we come here*…* Like I have said, there are a lot of things here. They can show us things, even the counsellors here they know how to talk with you*…* Female, young adult (IDI 3)

Other participants expressed positive sentiments in their perception of HIV risk as the clinical trial progressed:

There is a difference now. I feel differently even though I sometimes feel nervous but my participation in the study has uplifted my hopes of not acquiring HIV. No matter what can happen, but I sometimes feel slightly nervous of being infected with the disease. Female, young adult (IDI 1)

The client-centred counselling at every visit helped build trust and reinforce adherence and motivation. The participants were motivated to keep coming and recognise the HIV risk they had previously exposed themselves to.

By using trust as a cornerstone within this framework, we identified the factors that shape the HIV risk perception of participants involved in this clinical trial. Our results reveal that the presence or absence of trust played a pivotal role in guiding participants as they made decisions about their health behaviours. Participants were motivated to join the clinical trial and engage with HIV prevention methods such as PrEP. Additionally, male participants acknowledged a heightened perception of HIV risk, primarily due to engaging in risky sexual behaviours that could lead to potential infection. Conversely, females attributed their HIV risk to their partners, expressing concern about the risk posed by partners who engaged in infidelity with multiple individuals.

### Confidence

In this analysis, we used both the second and third interviews to explore participants’ expressed confidence in participating in the clinical trial and using PrEP, emphasising the significance of engaging with the trial in shaping their health behaviours related to HIV. Using the concept of confidence, we looked at how participants continued following the study procedures and the advice given by the health personnel, adherence to PrEP and, as a result, they started to perceive themselves at low risk of getting infected with HIV. Regular communication and counselling between clinical trial staff and participants contributed to confidence and assurance.

But it helped being here because last year she was doing things that I didn’t understand. It was with different people, so can you imagine if I was not on the study… I don’t want to lie I no longer make any mistakes, since you told me I do not. I make sure that I use a condom because you never know, you never know if you are using the real vaccine. Male, young adult (IDI 2)

The enrollment of this participant in the clinical trial increased their awareness of the seriousness of the consequences of their previous actions. In interview 2, they acknowledged that they did not fully understand the risks they were taking in the past. However, ongoing participation in the trial increased their confidence and changed their perception, leading to positive health behaviours.

I only perceived risk after joining the study as I am a grown up, I now see things differently. I now see that I used to put myself at risk that’s why I thought that there should be something to help myself prevent from acquiring HIV because life is rough outside. Male, young adult (IDI 2)

A growing self-confidence in the knowledge of their own HIV status was also noticed among the participants in the clinical trial. They highlighted that the quality of care they received gave them the confidence that they had not acquired HIV.

Er, first of all what makes me to continue with the study is that I will always know that I am not sick. I am always here, you see that thing, ja. That is what I like about here also the treatment here there are no problems, they all right. Male young adult (IDI 2)

The belief and trust in the competence of the clinical trial team built the participants’ confidence.

Individuals are more likely to adopt health-related behaviours when they have confidence that those behaviours will effectively prevent or reduce a health threat—in this case, acquiring HIV as indicated below, “I only know that PrEP is one thing, it’s all right when you are about to have sex with a woman, you take it. This is so that you don’t get HIV. That is what it’s all right about it, they explained to me like that here.” Male, young adult (IDI 3). Participants’ confidence seems to come from the clear and concise explanation provided by the study staff, indicating that comprehensive and accessible information plays a crucial role in building confidence in preventive measures such as PrEP. In the second phase of the interviews, participants reflected a positive change in behaviour influenced by their involvement in the intervention:

I am behaving well, and it is nice because I know that I have a date to come here. I have stopped cheating; I am no longer involved with women because I saw that I will be infected so it best I move away from them*…* Male, young adult (IDI 2)

Participants also showed some confidence attributed to taking oral PrEP consistently every day. This confidence contributed to them having a low HIV risk perception**,** “Yes, at I was at high risk. Ay, the boys are not trustworthy, they’re really not to be trusted!*…* Yes, but it’s no longer the same way than before. Maybe taking pills every day,… it is no longer the same as before.” Female, late adolescent (IDI 2).

The participant acknowledged that they were at high risk in the past. As a result of trial participation, they now take PrEP pills daily as a preventive measure.

Lack of confidence in the ability of public health facilities to provide PrEP was highlighted by participants. Participants reflected on the importance of access to PrEP as important in adherence. They expressed concern at the prospect of the study ending and the potential unavailability of and access to PrEP through public clinics.

I do not want to lie; I would really feel sad if the study were to come to an end and there is no other way of continuing with PrEP because when you ask, most of the public clinics do not offer PrEP and even though they have done the training for PrEP but they haven’t started to receive it or to distribute it and so it will really be difficult for us who are really relying on it and as we have gotten used to it. Female, young adult (IDI 1)

Our results indicate that the trust put in the health services provided by the clinical trial encouraged cooperation among participants and instilled confidence in the support provided.

### Cooperation

Cooperation involves the act of working together with others towards common goals or objectives.^15^ Our results showed participants cooperated with the trial by taking PrEP, attending counselling sessions and adhering to the study procedures. They recognised the potential seriousness of HIV transmission and the implications of not knowing one’s HIV status. Consequently, as the study progressed, participants adjusted their behaviour by emphasising moral responsibility and cooperation:

Currently because I am part of this research study, I have to conduct myself in a good moral manner because at the end of the day, you cannot know or tell any of the persons you meet their HIV status and you cannot convince them either for them to know their HIV status by having their test done. Female, young adult (IDI 2)

Results also showed that participants reported a reduced risk of HIV infection due to their committed participation in the study and access to preventive measures such as oral PrEP and participation in the HIV vaccine trial, which influenced their behaviour and choices. The individuals believed that they were not infected and that their daily pill regimen protected them, indicating a lower perception of their susceptibility to the virus. Their actions of committing themselves to the trial and adherence to PrEP indicated a level of cooperation built on trusting PrEP and engaging in the trial was good for them and their health, “I must perceive the possibilities because I don’t know what he is doing, or what the girl is doing, where and with who. So, I can say that it’s one of the things motivating me to keep on taking PrEP for prevention.” Female, late adolescent (IDI 2). Participants trusted PrEP when they were taking it during the clinical trial.

The perception that their risk of HIV had reduced was indicated by participants altering their behaviour to avoid situations that could lead to further HIV transmission. Their recognition of the implications of being infected influenced their actions, leading them to distance themselves from behaviours that might increase their HIV risk. This aligns with the TCC principle that trust and confidence lead to cooperation and positive health-related behaviours. “It makes me happy to know that I am still behaving appropriate; yes, it is good to know your status.” Male, late adolescent (IDI 3). Their perception of individual HIV risk became less because they believed that there was a protective role played by joining the clinical trial study, testing regularly and taking oral PrEP.

Since I joined the study, everything has been okay, when was it… I was here on Wednesday, I got tested again and they found that I am okay (HIV negative) and my blood is clean. It’s just fine and nice, there is no challenge. Male, young adult (ID1 2)

The individual’s engagement in the study, evidenced by their regular attendance and adherence to testing protocols, reflects a willingness to cooperate with the research.

However, in the study, there were people who discontinued PrEP who said that they had to stop taking PrEP because of the side effects they experienced.

Whenever I took PrEP tablets, if I didn’t take them, I won’t have anything, but once I take it at 20:00, I would know that I will not sleep… Ey, I took them for the first three days, then maybe two weeks passed by. I tried them again, wanting to see the actual cause of my sickness. As I have had both the tablets and the injection when I started, I wasn’t sure which one was making me sick. So, I continued taking the tablets for two days for that following two weeks. Still, the something happened, that’s when I stopped taking them, I never tried them again. Female, young adult (IDI2)

Even though these participants had the intention of cooperating with the clinical trial, these side effects posed a barrier to participation.

## Discussion

Using the TCC framework, we were able to highlight the links between perceived HIV risk and PrEP uptake in an HIV prevention clinical trial. Findings from this nested qualitative study revealed that the ongoing participation in the clinical trial influenced participants’ decision to continue PrEP as a preventive action based on the trust that it would reduce their susceptibility to HIV and HIV infection. This was achieved by enhancing their awareness of their vulnerability to HIV risk, offering HIV prevention education within a clinical trial setting facilitated by counselling and providing access to various HIV prevention methods. By increasing their knowledge about risk, participants were able to exercise caution and manage it through, for example, reducing the number of sexual partners they had, adhering to PrEP and following the clinical procedures of regular counselling and HIV testing as observed in other studies.^1121^,^23^

Second, people tend to trust information from sources they believe to be reliable and credible. Furthermore, when individuals trust the sources of risk information, for example, research scientists, clinical trials or government agencies, they are more likely to accept and act on the information provided, even if the risk is uncertain or complex. In this study, acquiring information about risk factors and HIV prevention methods helped participants to positively change their sexual behaviours leading to a low-HIV-risk perception. This was shown in other studies that focused on high-HIV-risk population and PrEP uptake such as men who have sex with men.^11 24^ The critical role of trust in shaping healthcare practices and preferences was also observed in an ethnographic study conducted by Birungi^25^, where trust in medical interventions facilitated engagement with care and use of injectables in the Ugandan health system.^25^

Participants in this study enrolled in the clinical trial and followed HIV prevention methods because they were uncertain about their partner’s HIV status and lacked trust in their partner. Similar patterns were found in qualitative and mixed methods studies conducted among participants of clinical trials in South Africa, where participants’ perspectives on trust in relationships and its impact on adopting preventive measures were examined. A lack of trust in sexual partners was observed as the main motivator to take oral PrEP which was viewed positively as an empowering option that afforded choice and control over sexual health.^9 24^

Gendered perceptions of HIV risk were observed in our study. Males expressed awareness of the risk they were taking and the consequences on their partners. On the contrary, women perceived themselves as at risk of HIV acquisition as a result of their partners’ risky behaviours. An increased level of trust in the clinical trial and PrEP was evident among male participants, who acknowledged how their involvement in the trial positively influenced their behaviour. A similar study was conducted to assess ‘gendered’ perceptions of HIV risk among young women and men in a high-HIV-prevalence community in South Africa, revealing differing HIV risk perceptions.^21^

Participants further emphasised a level of trust in the clinical trial staff, acknowledging that the assistance provided by the staff members has been instrumental in helping them adhere to PrEP. Trust was also cultivated by the clinical trial community and public engagement teams during recruitment and during the ongoing trial who engaged with the communities sharing HIV prevention information on the services provided by the clinical trial. Community engagement teams offered pre-screening workshops prior to trial participation. In addition, the clinical trial site was known in the community as a place where several studies had been conducted. The contribution of trust in health sector relationships can improve collaboration and demand for health services. Other studies conducted in low- to middle-income countries have also indicated that patient-provider trust leads to healthcare responsiveness.^26^,^28^

Our results highlighted concern over post-trial access to PrEP. This has been highlighted in other studies with participants stating several health system barriers such as long queues at public health facilities and transport costs associated with travel to clinics. A study conducted among adolescent girls in Kenya and South Africa showed that barriers to oral PrEP included being unable to collect PrEP refills due to school commitments and lacking transport to fetch PrEP from clinics.^29^ Differentiated models that include mobile and community-based platforms for oral PrEP delivery can be considered as these might address facility-based barriers to oral PrEP access.^30 31^

Barriers to uptake of PrEP have been recorded in our study, including people reporting side effects from PrEP leading to discontinued use. Interpersonal barriers due to, for example, resistance from family members to PrEP uptake, were also reported, which corroborates the findings from other studies.^32^ Such barriers persisted in the clinical trial despite supportive services such as counselling and constant health monitoring.

The PrEPVacc clinical trial required participants to undergo regular HIV testing and health check-ups, and this monitoring ensured that individuals remained engaged with their health and aware of their HIV status. Providing education, access to preventive measures and counselling was beneficial because it promoted cooperation from participants and positive health behaviours. This consistent health-seeking behaviour over time reinforced the trust and willingness to continue engaging in PrEP and HIV prevention measures such as regular condom use among participants. A literature review on the role of trust in healthcare systems in sub-Saharan Africa by Østergaard also showed that trust goes beyond the medical expertise and communication skills of healthcare providers or the reputation of a facility. It is built over time through consistent experiences that give people reasons to trust the provider and the institution.^26^

### Strengths and limitations of this study

The study’s long-term design and our case analysis approach helped us thoroughly explore each person’s stories and assess their unique situations regarding HIV risk and PrEP adherence. However, our sample size was limited to the clinical trial population which may not be generalisable to a broader population. The social science team made an effort to ensure that the sample would capture a variety of experiences within the trial, and we were able to see common themes across the coded data indicating that we were able to gather sufficient data on our areas of focus. It would be an interesting/constructive exercise to investigate further the broader population, outside of clinical trial settings, to provide a more comprehensive understanding of PrEP uptake in diverse contexts. Another limitation was our analysis of HIV risk perception based on the TCC framework which is mainly cognitive. A broader analysis could be adopted which also looks at other factors that influence HIV risk perception and PrEP adherence, such as societal and environmental factors.

## Conclusion

In this study, we employed the TCC framework which has shed light on the relationship between perceived HIV risk and PrEP adherence within the context of the PrEPVacc clinical trial. The findings have demonstrated that ongoing participation in the clinical trial coupled with trust significantly influenced individuals’ decisions to continue PrEP as a preventive measure. This decision was rooted in their trust that PrEP would reduce their vulnerability to HIV infection. Clinical trial information on HIV risk factors and PrEP contributed to trust and reduced HIV risk. Clear and concise communication when explaining HIV preventive measures like PrEP enhances participants’ understanding, trust and confidence in its effectiveness.

## Supplementary material

10.1136/bmjopen-2024-086742 online supplemental file 1

10.1136/bmjopen-2024-086742 online supplemental file 2

## Data Availability

Data are available upon reasonable request.
