# Supplementary material for: HIV risk perception, trust and PrEP adherence among participants in an HIV prevention trial: a qualitative longitudinal study, South Africa
Source: BMJ Open. 2025 Apr 23;15(4):e086742. doi: 10.1136/bmjopen-2024-086742 (PMC12020760; doi:10.1136/bmjopen-2024-086742)
Supplement: online supplemental file 1 [file bmjopen-15-4-s001.pdf]

PrEPVacc
A Phase IIb three-arm, two-stage HIV prophylactic vaccine trial with a second randomisation to compare TAF/FTC to TDF/FTC as pre-exposure prophylaxis.

Version: 5.0
Date: 29 September 2021

MRC/UVRI and LSHTM ID: PV1
NCT #: NCT04066881

Authorised by:
Name: Pontiano Kaleebu
Role: Coordinating Investigator
Signature:
Date:

Name: Christian Hansen
Role: Trial Statistician
Signature:
Date:

Name
Role: National Principal Investigator
Signature:
Date

This protocol has been produced using MRC CTU at UCL Protocol Template version 6.0. The template, but not any study-specific content, is licensed under a Creative Commons Attribution 4.0 International License (https://creativecommons.org/licenses/by/4.0/). Use of the template in production of other protocols is allowed**,** but MRC CTU at UCL must be credited.

GENERAL INFORMATION

This document was constructed using the MRC CTU at UCL Protocol Template Version 6.0 which endorses the Standard Protocol Items: Recommendations for Interventional Trials (SPIRIT) initiative. It describes the PrEPVacc trial, coordinated by the Medical Research Council (MRC) Uganda Virus Research Institute (MRC/UVRI) and London School of Hygiene and Tropical Medicine (LSHTM) Uganda Research Unit, and provides information about procedures for entering participants into the trial. The protocol should not be used as an aide-memoire or guide for the treatment of others. Every care has been taken in drafting this protocol, but corrections or amendments may be necessary. These will be circulated to the registered investigators in the trial, but sites entering participants for the first time are advised to contact MRC/UVRI and LSHTM Uganda Research Unit to confirm they have the most up-to-date version.

COMPLIANCE

The trial will be conducted in compliance with the approved protocol, the Declaration of Helsinki 1996 **fourth revision** the principles of GCP as laid down by the ICH topic E6 (Note for Guidance on GCP) and applicable national regulations.

SPONSOR

Imperial College, London is the trial Sponsor and has delegated responsibility for the overall management of the trial to the MRC/UVRI and LSHTM Uganda Research Unit. Queries relating to sponsorship of this trial should be addressed to jrcocoordinator@imperial.ac.uk or via the Trial Management Team.

FUNDING

The trial is funded through the second European Developing Country Clinical Trial Partnership (EDCTP2) under grant reference RIA2016V-1644.

Gilead Sciences plc will provide the TDF/FTC and TAF/FTC.

AUTHORISATIONS AND APPROVALS

The study protocol and supporting documents will be submitted to the national regulatory authorities and ethics committees for review. The study will only start to recruit participants when authorisation and approval have been obtained in that country.

TRIAL REGISTRATION

This trial has been registered with the **Clinicaltrials.gov** Clinical Trials Register, where it is identified as **NCT04066881.**

RANDOMISATIONS
To randomise, details to be added when available

EXPEDITED REPORTING
Within 24 hours of becoming aware of an SAE or Notable Event, please send a completed form securely to:
PrEPVacc_trial_safety@mrcuganda.org

TRIAL ADMINISTRATION

Please direct all queries to the **Trial Managers** at PrePVacc-MRC@mrcuganda.org and mrcctu.prepvacc@ucl.ac.uk in the first instance; clinical queries will be passed to the Coordinating Investigator/Trial Physician/another member of the Trial Safety Group (TSG) by the Trial Managers.

COORDINATING INVESTIGATOR

Prof Pontiano Kaleebu<br>Plot 51-59 Nakiwogo Road,<br>PO Box 49,<br>Entebbe,<br>Uganda	Email:	**************
	Tel:	+256 (0) 417 704103

COORDINATING DATA CENTRE (MRC/UVRI AND LSHTM UGANDA RESEARCH UNIT)

Trial Statistician<br>Christian Hansen<br>Plot 51-59 Nakiwogo Road,<br>PO Box 49,<br>Entebbe,<br>Uganda	Email:	**************
	Tel:	+256 (0) 417 704000

INDEPENDENT OVERSIGHT

Chair of Trial Steering Committee	Prof Chris Conlon, Oxford University, Oxford, UK
Chair of the Independent Data Monitoring Committee	Dr Doug Taylor, FHI, Durham, USA

Full details of all trial teams, groups and committees are held in a separate document

SUMMARY OF TRIAL

SUMMARY INFORMATION TYPE	SUMMARY DETAILS
**Short title**	PrEPVacc
**Long Title of Trial**	A Phase IIb three-arm, two-stage HIV prophylactic vaccine trial with a second randomisation to compare TAF/FTC to TDF/FTC as pre-exposure prophylaxis.
**Version**	5.0
**Date**	29 September 2021
**MRC/ UVRI ID**	PV1
**NCT Number**	NCT04066881
**Study Design**	This international, multicentre, double-blind vaccine study will be a three-arm prospective 1:1:1 randomisation comparing each of two experimental combination vaccine regimens with placebo control.<br><br>Pre-screening for risk and HIV status will take place as part of a Registration Cohort which will precede and continue in parallel to PrEPVacc enrolments. This will give HIV negative volunteers time to learn about the PrEPVacc trial and facilitate timely enrolment.<br><br>Clinical screening for the vaccine trial will take place during the 8 weeks prior to randomisation. Eligible participants will be enrolled at week 0 and randomised to one of three vaccine arms:<br>▶ DNA-HIV-PT123 and AIDSVAX® B/E (target wks 0,4,24,48)<br>▶ DNA-HIV-PT123 and CN54gp140+MPLA-L (target wks 0,4), then MVA-CMDR and CN54gp140+MPLA-L (target wks 24,48)<br>▶ Saline placebo (target wks 0,4,24,48)<br>See Table 1 for details of the schedule and windows<br><br>There will be a concurrent 1:1 randomisation comparing two PrEP regimens, open-label:<br>▶ Daily TDF/FTC<br>▶ Daily TAF/FTC<br><br>Participants will continue to receive study PrEP through to 2 weeks after their third immunisation after which access to PrEP will revert to locally registered supply of generic drug.<br><br>The Independent Data Monitoring Committee will review safety data every 6 months and conduct one interim analysis of vaccine efficacy in order to determine whether each active vaccine arm has demonstrated sufficient efficacy to warrant further investigation. This analysis will

SUMMARY INFORMATION TYPE	SUMMARY DETAILS
	only consider new infections arising after the visit 2 weeks beyond the third immunisation and only those in individuals who have completed the first three immunisations. The analysis will take place after approximately 7 of these infections have occurred in the placebo group. If both experimental arms continue as planned the study will recruit approximately 556 participants to each arm, a minimum of 1668, each followed for a minimum of 74 weeks from enrolment.<br><br>The investigators will not be informed of the timing of the interim analysis, unless there is a recommendation to modify the protocol.<br><br>The PrEP analysis will consider new infections up to and including the visit 2 weeks after the third immunisation in individuals who were HIV negative at enrolment. The IDMC will review safety and effectiveness data every 6 months and may recommend an increase in the numbers randomised to the PrEP component alone.
**Setting**	Clinical research centres and their local communities in, South Africa, Tanzania and Uganda
**Type of Participants to be Studied**	The study aims to randomise a minimum of 1668 eligible individuals through collaborating clinical research centres in 3 countries (South Africa, Tanzania and Uganda), according to the following criteria:<br><br>**Inclusion criteria:**<br>1. HIV uninfected adults aged 18-40 years old<br>2. Willing and able to provide informed consent to participate<br>3. Willing and able to comply with the visit schedule and provide blood, urine and other samples<br>4. Available for at least 82 weeks from screening<br>5. Likely to be at risk from exposure to HIV during follow-up<br>6. Willing to undergo HIV testing, receive HIV test results and risk reduction counselling which includes promotion of PrEP and condoms<br>7. If female and of childbearing age and not sterilised, willing to use a highly effective method of contraception from screening until 18 weeks after last injection<br>8. If male and not sterilised, willing to avoid impregnating female partners from screening until 18 weeks after last injection<br><br>**Exclusion criteria:**<br>1. HIV infection or indeterminate HIV result at screening or enrolment<br>2. Hepatitis B surface antigen positive

SUMMARY INFORMATION TYPE	SUMMARY DETAILS
	3. If female, currently pregnant (positive serum or urine pregnancy test), or lactating<br>4. Participating in another biomedical or HIV testing research study, or receipt of a live vaccine within 30 days of randomisation<br>5. Participation in a previous HIV vaccine or HIV immunotherapy trial<br>6. Receipt of blood products or immunoglobulins within 12 weeks of screening<br>7. Known hypersensitivity to any component of the vaccine formulations used in this trial or history of severe or multiple allergies to vaccines, drugs or pharmaceutical agents<br>8. Presence of systemic disease at the time of randomisation or history of chronic illness that in the opinion of the investigator may compromise the volunteer's safety, preclude vaccination or compromise an immune response to vaccine<br>9. Grade 2 and above abnormalities in routine laboratory parameters using the DAIDS toxicity table, version 2.1 July 2017 or estimated glomerular filtration rate less than 60ml/min
**Ancillary Studies/Substudies**	None
**Sponsor**	Imperial College, London
**Sponsor's Legal Representative**	[National Principal Investigator]
**Interventions to be Compared**	**Vaccine Group A:**<br>▶ DNA-HIV-PT123 and AIDSVAX® B/E (target wks 0,4,24,48)<br>**Vaccine Group B:**<br>▶ DNA-HIV-PT123 and CN54gp140+MPLA-L (target wks 0,4), then MVA-CMDR and CN54gp140+MPLA-L (target wks 24,48)<br>**Vaccine Group C:**<br>▶ Saline placebo (target wks 0,4,24,48)<br><br>**Control PrEP:**<br>▶ TDF/FTC once daily (from wk 0 to 2 weeks after the third immunisation)<br>**Experimental PrEP:**<br>▶ TAF/FTC once daily (from wk 0 to 2 weeks after the third immunisation)
**Study Hypothesis**	This trial is designed to detect a reduction in HIV incidence that has public health relevance sufficient to justify implementation of the combination vaccine regimen. In light of the high level of effectiveness demonstrated in the PrEP trials (up to 86% reduction in HIV), this trial is

SUMMARY INFORMATION TYPE	SUMMARY DETAILS
	powered to detect a protective vaccine efficacy of 70% at the final analysis.<br><br>The evidence suggests that TAF/FTC will be equivalent to or better than TDF/FTC at averting HIV infections. The PrEP component aims to show the effectiveness of TAF/FTC is not unacceptably lower than the effectiveness of TDF/FTC, assessed from the observed lower confidence limit for the Averted Infections Ratio (AIR).
**Objectives and Primary Outcome Measure(s)**	**Objectives**<br>▶ To determine the vaccine efficacy of each combination regimen compared to placebo in preventing acquisition of HIV<br>▶ To determine the effectiveness of TAF/FTC PrEP as a function of the effectiveness of TDF/FTC PrEP and hypothetical placebo<br>▶ To determine the safety of (i) each vaccine combination regimen compared to placebo and (ii) TAF/FTC compared to TDF/FTC<br>▶ To understand the trial results in the context of risk and adherence behaviours, knowledge attitudes and perceptions of participants, study staff and the wider community<br><br>**Primary Efficacy Outcome for the vaccine analysis**<br>▶ HIV acquisition by a participant who completed the first three immunisations and was HIV negative at the visit 2 weeks after the third immunisation.<br><br>**Primary Efficacy Outcome for the PrEP analysis**<br>▶ HIV acquisition at or before the visit 2 weeks after the third immunisation by a participant who was HIV negative at enrolment.<br><br>**Primary Safety Outcome for the vaccine and PrEP analyses**<br>▶ A clinical decision to discontinue the vaccine or PrEP regimen for an adverse event that is considered related to product
**Secondary Safety and Other Outcome Measure(s)**	**<u>Vaccine analysis:</u>**<br>**Secondary Safety Outcomes:**<br>▶ Grade 3 and above solicited adverse events which last more than 72 hours<br>▶ Grade 3 and above laboratory adverse events that are confirmed on repeat testing if possible<br>▶ An adverse event leading to a clinical decision to discontinue or interrupt the product schedule, regardless of relationship<br>▶ Serious Adverse Events

SUMMARY INFORMATION TYPE	SUMMARY DETAILS
	▶ Other clinical and laboratory adverse events<br><br>**Secondary Immunological Outcomes:**<br>▶ Binding antibodies to CN54gp140 and AIDSVAX® B/E gp120<br><br>**PrEP analysis:**<br>**Secondary Safety Outcomes**<br>▶ An adverse event leading to a clinical decision to discontinue or interrupt the product schedule, regardless of relationship<br>▶ Serious adverse events<br>▶ Genotypic resistance at HIV seroconversion, focussing on the mutations selected by tenofovir and emtricitabine (codons 65, 70, 184 in reverse transcriptase)<br><br>**Secondary Adherence Outcomes:**<br>▶ Adherence assessed by (a) self-report (b) results of point of care urine tests (c) TFV DP levels measured on dry blood spots (DBS) in red blood cells (d) total number of pills dispensed<br><br>Specimens will be collected as per **Table 1** and processed for secondary analyses or stored for future exploratory analyses, subject to funding (see **Section 9.2.1.C**).
**Randomisation**	Participants will be randomised at each clinical centre through web randomisation after entering the eligibility criteria. Randomisation will be stratified by centre and by gender for vaccines and PrEP.<br><br>Clinic staff and participants will be blind to allocation of active or placebo vaccines, but the pharmacist preparing the vaccines will know.<br><br>As the volume of CN54gp140 in MPLA-L is 0.45ml and given at the same timepoints as products with a volume of 1ml, clinic staff will be able to identify participants allocated to this CN54gp140 in MPLA-L or matched placebo.<br><br>Clinic staff and participants will know which PrEP agent each participant is allocated to.
**Number of Participants to be Studied**	The vaccine component of the trial requires a minimum of 1668 eligible individuals to support the final analysis. They will be drawn from key populations attached to one of the participating clinical research centres (see **Section 3.3** for more details).

SUMMARY INFORMATION TYPE	SUMMARY DETAILS
**Duration and blood volume**	Participants continue from the screening visit (up to 8 weeks before enrolment) to the last visit, a minimum of 74 weeks (around 17 months) after enrolment.<br>679mls of blood will be collected from screening to 74 weeks of follow-up inclusive. Thereafter, 10mls will be collected every 12 weeks for HIV testing, with an additional 4mls for DBS and 5mls every 24 weeks for syphilis serology (if indicated), through to the final visit in the last 3 months of the trial.<br>If repeat safety or HIV tests are needed then up to 14ml and 10 mls respectively will be drawn.<br>If HIV status is confirmed as positive, 61ml of additional blood will be required at this visit for humoral and cellular responses virological analysis, including drug resistance testing.

PREPVACC SCHEMA

Figure 1: Trial Entry, Treatment, and Follow-up

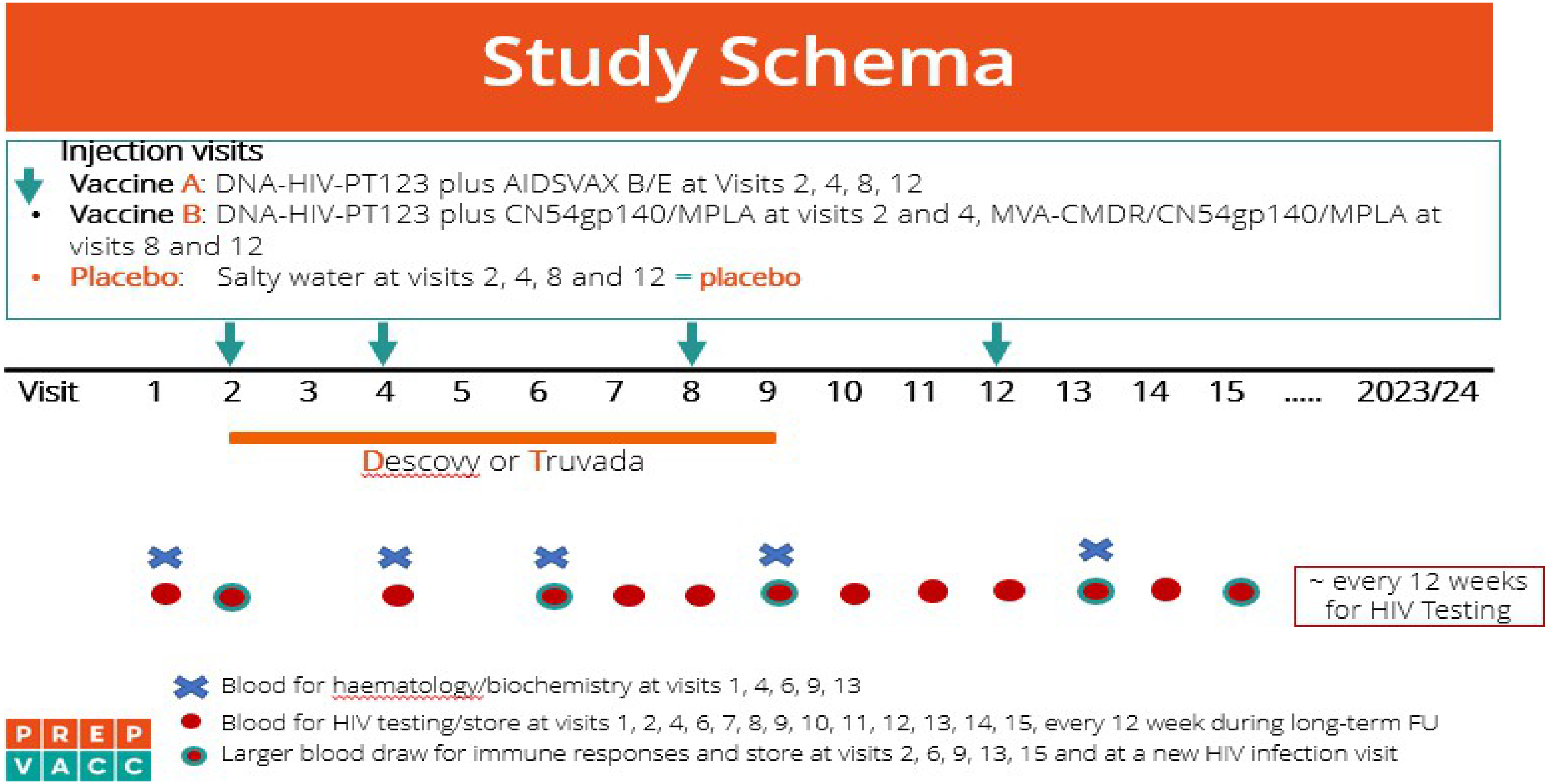

PREPVACC ASSESSMENT SCHEDULE

Table 1: Trial Assessment Schedule for Investigators (the target illustrates the ideal time for the gaps between immunisations)

Visit number (V)	1	2	3	4	5	6	7	8	9	10	11	12	13	14	15	Follow-up after week 74		
Target (wks or Visit +wks)	-8	0	1	4	V4+1	V4+4	V4+12	V4+20	V8+2	V8+6	V8+14	V8+24	V12+2	V12+14	V12+26	Every 12wks	Every 24wks	Final
Upper Target			2	7	V4+2	V4+5	V4+16	V4+22	V8+4	V8+7	V8+18	V8+26	V12+4	V12+18	V12+32	+6	+6	
Earliest timepoint			1-4d	3	V4+3d	V4+3	V4+11	V4+17	V8+1	V8+5	V8+13	V8+19	V12+1	V12+10	V12+22	-6	-6	
Latest timepoint for immunisation/visit		0		End of wk13		V8-1		End of wk40	V12-1			End of wk68			End of trial			
Immunisation		x		x				x				x						
PrEP dispensed		x ←		x			x	(x)	→									
Screening	x																	
Adverse events		x	x	x	x	x	x	x	x	x	x	x	x	x	x	SAEs	SAEs	SAEs
Weight	x	x		x				x				x						
Hepatitis B surface Ag	x																	
Routine labs for safety	x	(x)[1]		x		x	(x)[1]	(x)[1]	x	(x)[1]		(x)[1]	x					
Pregnancy test	x	x		x		x	x	x	(x)[1]	x	x	x	(x)[1]	x	x			
Urine test for TFV	x			x			x					x			x		x	
TFV DP levels in RBCs[2]									x			x			x		x	
Risk and/or adherence	x			x			x	x	x	x		x		x	x		x	
STI screen[3]	x								x			(x)			(x)		(x)	
HIV and store	x	x		x		x	x	x	x	x	x	x	x	x	x	x	x	x
Immunology and store [4]		x				x			x				x		x			
Store for HLA		x																
Total blood volume(ml)[5]	29	133	0	24	0	43	10	10	138	10	10	14	129	10	119	10	14	10

SAE= Serious Adverse Events: information will be collected throughout

[1] (x) additional routine safety or pregnancy tests may be needed to follow-up a side effect or previously abnormal value. Routine safety labs recorded are Hb, creatinine, AST/ALT, alkaline phosphatase, total Bilirubin and glucose.

[2] The level of tenofovir diphosphate can be assessed in red blood cells using dry blood spots.

[3] STI screen will comprise serology for syphilis (TPHA performed if positive), NAATs for GC and CT on urine in men and women, and self-collected rectal swabs in both if indicated. After visit 9 these will be done only if indicated.

[4] Immunology comprises serum for antibodies collected at every timepoint indicated and peripheral blood mononuclear cells and plasma collected at visits 2, 9, 13 and 15.

[5] This does not include any additional blood volume required if a safety lab or HIV test needs to be repeated (up to 14mls and 10mls respectively).

CONTENTS

GENERAL INFORMATION........ ii

SUMMARY OF TRIAL........ iv

PREPVACC SCHEMA........ x

PREPVACC ASSESSMENT SCHEDULE xi

CONTENTS ....

ABBREVIATIONS........

BACKGROUND 1
1.1 THE GLOBAL HIV-1 EPIDEMIC AND THE NEED FOR A PROPHYLACTIC VACCINE........ 1
1.2 PROPHYLACTIC VACCINE EFFICACY TRIALS TO DATE........ 1
1.3 PREP 2
1.4 RESEARCH LEADING UP TO THE PROPOSED TRIAL........ 2
1.4.1 DNA-HIV-PT123........ 2
1.4.2 AIDSVAX® B/E........ 4
1.4.3 CN54gp140 in GLA-AF or MPLA-L........ 5
1.4.4 MVA-CMDR........ 7
1.4.5 Selection Of The Combination Vaccine Regimens........ 8
1.4.6 TAF/FTC As PrEP........ 9
1.5 OBJECTIVES AND RATIONALE FOR PROPOSED TRIAL........ 9
1.5.1 Objectives........ 9
1.5.2 Rationale........ 10

2 SELECTION OF SITES/CLINICIANS........ 12
2.1 SITE/INVESTIGATOR INCLUSION CRITERIA........ 12
2.1.1 Principal Investigator's Qualifications & Agreements........ 12
2.1.2 Adequate Resources........ 12
2.1.3 Site Assessment........ 13
2.2 SITE/INVESTIGATOR EXCLUSION CRITERIA........ 13
2.3 APPROVAL AND ACTIVATION........ 13

3 SELECTION OF PARTICIPANTS........ 14
3.1 PARTICIPANT INCLUSION CRITERIA........ 14
3.2 PARTICIPANT EXCLUSION CRITERIA........ 14
3.3 NUMBER OF PARTICIPANTS........ 15
3.4 CO-ENROLMENT GUIDELINES........ 15
3.5 SCREENING PROCEDURES & PRE-RANDOMISATION INVESTIGATIONS........ 15

4 REGISTRATION & RANDOMISATION........ 16
4.1 RANDOMISATION PRACTICALITIES........ 16
4.2 RANDOMISATION CODES & UNBLINDING........ 16

5 TREATMENT OF PARTICIPANTS 17
5.1 INTRODUCTION 17
5.2 COMBINATION GROUP A 17
5.2.1 DNA-HIV-PT123 17
5.2.2 AIDSVAX® B/E 18
5.2.3 Treatment Schedule 18
5.2.4 Dose and administration 18
5.2.5 Storage 18
5.2.6 Dispensing 18
5.2.7 Dose Modifications & Interruptions 18
5.3 COMBINATION GROUP B 19
5.3.1 DNA-HIV-PT123 19
5.3.2 MVA-CMDR 19
5.3.3 CN54gp140 19
5.3.4 MPLA Liposomes (MLPA-L) 20
5.3.5 Treatment Schedule 20
5.3.6 Dose and administration 20
5.3.7 Storage 20
5.3.8 Dispensing 21
5.3.9 Dose Modifications & Interruptions 21
5.4 GROUP C 21
5.4.1 Normal Saline Placebo 21
5.4.2 Treatment Schedule 21
5.4.3 Dose & administration 21
5.4.4 Storage 21
5.4.5 Dispensing 21
5.4.6 Dose Modifications & Interruptions 21
5.5 PREP DRUGS 22
5.5.1 TDF/FTC 22
5.5.2 TAF/FTC 22
5.5.3 Dose & administration 22
5.5.4 Storage 22
5.5.5 Dispensing and Returns 22
5.5.6 Dose Modifications & Interruptions 22
5.6 UNBLINDING / UNMASKING 23
5.7 PROTOCOL TREATMENT DISCONTINUATION 23
5.8 ACCOUNTABILITY & UNUSED VACCINES AND DRUG 24
5.9 COMPLIANCE & ADHERENCE 24
5.9.1 Injections 24
5.9.2 PrEP 24
5.10 TREATMENT DATA COLLECTION 25
5.11 NON-TRIAL TREATMENT 25
5.11.1 Medications Not Permitted 25
5.11.2 Medications To Be Used With Caution In Conjunction With PrEP 25
5.11.3 Other Vaccinations 25
5.11.4 Treatment After HIV Seroconversion 26
5.12 CO-ENROLMENT GUIDELINES 26

6 ASSESSMENTS & FOLLOW-UP 27
6.1 TRIAL ASSESSMENT SCHEDULE 27
6.2 PROCEDURES DURING THE SCREENING PERIOD 28

6.2.1 Informed Consent ... 28
6.2.2 Eligibility ... 28
6.2.3 Investigations ... 29
6.3 PROCEDURES AT ENROLMENT ... 29
6.3.1 Eligibility ... 29
6.3.2 Randomisation ... 30
6.3.3 Injection ... 30
6.3.4 PrEP ... 30
6.4 TREATMENT PROCEDURES DURING FOLLOW-UP ... 31
6.4.1 Injections ... 31
6.4.2 PrEP ... 31
6.5 PROCEDURES FOR ASSESSING SAFETY OF TRIAL TREATMENTS ... 31
6.5.1 Solicited Adverse event assessment ... 31
6.5.2 Other Adverse event assessment ... 32
6.5.3 PrEP Adverse event assessment ... 33
6.5.4 Pregnancy assessment ... 33
6.6 PROCEDURES FOR ASSESSING ADHERENCE TO PREP ... 33
6.7 PROCEDURES FOR ASSESSING EFFICACY ... 34
6.7.1 Confirmation of HIV infection ... 34
6.7.2 Timing of HIV infection ... 35
6.8 PROCEDURES FOR ASSESSING IMMUNE RESPONSES ... 35
6.8.1 Secondary Immune responses ... 35
6.8.2 Exploratory Immune responses ... 35
6.8.3 Genotyping ... 36
6.9 PROCEDURES FOR ASSESSING CONTEXTUAL DATA ... 36
6.9.1 Self-Reported Sexual and Adherence Behaviours ... 36
6.9.2 Contextual Data In Seroconvertors ... 37
6.10 EARLY STOPPING OF FOLLOW-UP ... 37
6.11 PARTICIPANT TRANSFERS ... 37
6.12 LOSS TO FOLLOW-UP ... 38
6.13 COMPLETION OF PROTOCOL FOLLOW UP ... 38
6.14 POST-TRIAL HIV TESTING ... 38

7 SAFETY REPORTING ... 39
7.1 DEFINITIONS ... 39
7.1.1 Medicinal Products ... 40
7.1.2 Adverse Events ... 40
7.1.3 Events That Are Not Adverse ... 40
7.2 OTHER NOTABLE EVENTS ... 40
7.2.1 Pregnancy ... 40
7.3 INVESTIGATOR RESPONSIBILITIES ... 40
7.3.1 Investigator Assessment ... 41
7.3.2 Notification Procedure ... 42
7.4 MRC/UVRI AND LSHTM RESPONSIBILITIES ... 42

8 QUALITY ASSURANCE & CONTROL ... 43
8.1 RISK ASSESSMENT ... 43
8.2 CENTRAL MONITORING AT MRC/UVRI AND LSHTM ... 43
8.3 ON-SITE MONITORING ... 43
8.3.1 Direct Access to Participant Records ... 43
8.3.2 Confidentiality ... 43

9 STATISTICAL CONSIDERATIONS ... 44
9.1 METHOD OF RANDOMISATION ... 44
9.2 OUTCOME MEASURES ... 44
9.3 SAMPLE SIZE ... 45
9.4 INTERIM MONITORING & ANALYSES ... 47
9.5 ANALYSIS PLAN (BRIEF) ... 47

10 ANCILLARY STUDIES ... 49

11 REGULATORY & ETHICAL ISSUES ... 50
11.1 COMPLIANCE ... 50
11.1.1 Regulatory Compliance ... 50
11.1.2 Site Compliance ... 50
11.1.3 Data Collection & Retention ... 50
11.1.4 Sample Storage and Retention ... 50
11.2 ETHICAL CONDUCT ... 51
11.2.1 Ethical Considerations ... 51
11.2.2 Favourable Ethical Opinion ... 51
11.3 COMPETENT AUTHORITY APPROVALS ... 52
11.4 OTHER APPROVALS ... 52

12 INDEMNITY ... 53

13 FINANCE ... 54

14 OVERSIGHT & TRIAL COMMITTEES ... 55
14.1 TRIAL MANAGEMENT GROUP (TMG) ... 55
14.2 TRIAL SAFETY GROUP (TSG) ... 55
14.3 TRIAL MANAGEMENT TEAM (TMT) ... 56
14.4 TRIAL STEERING COMMITTEE (TSC) ... 56
14.5 INDEPENDENT DATA MONITORING COMMITTEE (IDMC) ... 56
14.6 PARTICIPANT AND PUBLIC INVOLVEMENT ADVISORY GROUPS ... 56
14.7 ROLE OF STUDY SPONSOR ... 56

15 PARTICIPANT AND PUBLIC INVOLVEMENT ... 57
15.1 PPI STRATEGY ... 57
15.2 PPI CONTRIBUTORS ... 57
15.3 REPORTING AND EVALUATING IMPACT OF PPI ... 57

16 PUBLICATION AND DISSEMINATION OF RESULTS ... 58

17 DATA AND/OR SAMPLE SHARING ... 59

18 PROTOCOL AMENDMENTS ... 60

19 REFERENCES ... 63

ABBREVIATIONS

ABBREVIATION	DEFINITION	ABBREVIATION	DEFINITION
AE	Adverse event	HVTN	HIV Vaccine Trials Network
ADCC	antibody-dependent cell-mediated cytotoxicity	IAVI	International AIDS Vaccine Initiative
AIDS	Acquired Immune Deficiency Syndrome	IB	Investigator Brochure
AIR	Averted Infections Ratio	ICH	International Conference on Harmonisation of Technical Requirements for Registration of Pharmaceuticals for Human Use
AR	Adverse reaction	IDMC	Independent Data Monitoring Committee
ART	Antiretroviral therapy	IFN	Interferon
BAMA	Binding antibody multiplex assay	IL	Interleukin
CI	Co-ordinating Investigator	IMP	Investigational Medicinal Product
CRF	Case Report Form	IRB	Institutional Review Board
CT	Chlamydia	ITT	Intention-to-treat
CTU	Clinical Trials Unit	LAP	Laboratory Analytic Plan
DBS	Dry Blood spot	LPS	Lipopolysaccharide
DNA	Deoxyribonucleic acid	LSHTM	London School of Hygiene and Tropical Medicine
ECG	Electrocardiogram	MAMS	Multi Arm Multi Stage
ELISA	Enzyme-linked immunosorbent assay	MPLA	Monophosphoryl lipid A
FDA	(US) Food and Drug Administration	MRC	Medical Research Council
FTC	Emtricitabine	MRC CTU at UCL	Medical Research Council Clinical Trials Unit at University College London
GC	Gonorrhea	MRC/UVRI	Medical Research Council/Uganda Virus Research Institute
GCP	Good Clinical Practice	MVA	Modified vaccinia Ankara
GDPR	General Data Protection Regulation	nAb	Neutralising antibody
GMO	Genetically Modified Organism	NCT	ClinicalTrials.gov database identifier
HCG	Human Chorionic Gonadotropin	NIH	US National Institutes of Health
HIV	Human Immunodeficiency Virus	PBMC	Peripheral Blood Mononuclear Cell
HLA	Human Leukocyte Antigen	PI	Principal Investigator

ABBREVIATION	DEFINITION	ABBREVIATION	DEFINITION
PIS	Participant Information Sheet	SOP	Standard Operating Procedure
PK	Pharmacokinetics	SPC	Summary of Product Characteristics
PPI	Participant and Public Involvement	SUSAR	Suspected Unexpected Serious Adverse Reaction
PrEP	Pre-Exposure Prophylaxis	TAF	Tenofovir alafenamide
QA	Quality Assurance	TDF	Tenofovir Disoproxil Fumarate
QC	Quality Control	TFV	Tenofovir
RBC	Red Blood Cell	TMG	Trial Management Group
SAE	Serious Adverse Event	TMT	Trial Management Team
SAR	Serious Adverse Reaction	TSC	Trial Steering Committee
SMP	Safety Management Plan	TSG	Trial Safety Group
SOM	Study Operations Manual	UAR	Unexpected Adverse Reaction

BACKGROUND

1.1 THE GLOBAL HIV-1 EPIDEMIC AND THE NEED FOR A PROPHYLACTIC VACCINE

According to the 2017 UNAIDS figures, there are approximately 36.9 million people worldwide living with HIV/AIDS, with 1.8 million children currently affected[1] . The vast majority of those infected live in Sub-Saharan Africa. An estimated 1.8 million people were newly infected with HIV in 2017, showing that this epidemic remains a major global health challenge. Significant efforts have been made to reduce the HIV prevalence by scaling up testing, offering immediate treatment or Pre-Exposure Prophylaxis (PrEP), but a prophylactic HIV vaccine is still considered to be necessary for the control of the epidemic.

1.2 PROPHYLACTIC VACCINE EFFICACY TRIALS TO DATE

There have been six HIV prophylactic vaccine efficacy trials to date. Two of the trials[2,3] evaluated a protein subunit vaccine (recombinant monomeric gp120) in populations at risk of infection in Thailand, North America and the Netherlands. The monomeric gp120 induced binding antibodies of high titre but no heterologous neutralizing antibodies (nAb) against primary HIV-1 isolates, and failed to show clinical efficacy. Subsequently, an adenovirus (Ad5) derived vaccine encoding *gag*, *pol* and *nef* with no envelope immunogen was evaluated in the Step and Phambili efficacy trials. In spite of strong cellular immune responses, these two trials were both stopped early for futility and there was a suggestion of increased infection risk in vaccinated males who had not been circumcised[4,5]. More recently, the HVTN505 trial evaluated a regimen of DNA prime and Ad5 boost, a regimen that also elicited robust cellular responses. Both vaccines expressed HIV Clade A, B and C gp140 Env in addition to Gag and Pol (Nef was expressed by the DNA only). This trial was also stopped early for futility[6].

RV144 is the only Phase III trial carried out to date to demonstrate efficacy[7]. The Thai RV144 study was a community-based, randomised, multicentre, double-blind, placebo-controlled trial consisting of two “priming” injections of a recombinant canarypox vector (ALVAC-HIV expressing *gag pro* and *env* genes) followed by two “boosting” injections combining AIDSVAX® B/E Envgp120 protein (formulated with alum) together with the ALVAC vaccine. In the modified intention-to-treat analysis excluding 7 of 16402 subjects found to be HIV positive at enrolment the investigators reported an overall vaccine efficacy over the 3.5 years of the trial of 31.2% (95% CI, 1.1%-52.1%; p=0.04). The modest efficacy observed in RV144 has invigorated the field and further analyses to identify potential immune correlates of infection suggest that IgG binding antibodies to the V1/V2 region of gp120 and ADCC mediated antibody responses are correlated with a decreased risk of acquisition[8.9]. Efforts are now focused on the design of new vaccine candidates and combination regimens that elicit more potent anti-V1V2 responses and improve the durability of the responses.

Building on the RV144 trial, the HIV Vaccine Trial Network (HVTN) launched an efficacy trial, HVTN702 (Uhambo) in Southern Africa, built on the RV144 findings and evaluating the combination of ALVAC with a subtype C protein subunit vaccine which is relevant to the HIV subtype that predominates in southern Africa.The study enrolled 5,407 HIV negative men and women through 14 centres in South Africa. The study team reported early when 60% of participants had completed 18 months of follow-up because the number of infections in the active and placebo groups were 129 and 123 respectively[10].

PrEPVacc is testing different vaccine candidates and vaccination regimens to HVTN 702. The lack of efficacy observed in HVTN 702 is disappointing, but is specific to that trial and cannot be generalised to other HIV vaccine candidates under investigation. The PrEPVacc trial remains important to the HIV vaccine field.

HVTN 705 (Imbokodo)[11] is a Phase IIb efficacy study investigating the efficacy of two administrations of tetravalent Ad26 vaccine (4 recombinant Ad26 vectors expressing mosaic inserts of HIV gag-pol or env genes) at months 0 and 3, followed by 2 administrations of the same tetravalent rAd26 vaccine with soluble trimeric Clade C gp140 formulated in alum at months 6, and 12- a combination previously shown to elicit robust T-cell and B-cell responses in line with those that correlated with robust protection in non-human primates (NHP).

1.3 PREP

TDF/FTC was first approved for use as pre-exposure prophylaxis (PrEP) by the US Food and Drug Administration (FDA) in July 2012 on the basis of two large randomised placebo-controlled trials, one in men who have sex with men (MSM) and transgender women recruited mainly from North and South America[12], and a second in serodiscordant couples recruited from Kenya and Uganda[13]. These two trials provided the pivotal data but they were supported by a third trial conducted in young heterosexual adults in Botswana[14]. Subsequent to these trials two European trials reported even higher levels of effectiveness in MSM, with no evidence of biological failure [15,16].

The post-market authorisation data collected in 10,609 individuals participating in 46 projects globally were presented in October 2018[17]. HIV incidence in these populations would be expected to exceed 3/100 person years (pyrs) without PrEP. There were 91 infections in 9,936 pyrs of follow-up, 27 of which occurred more than 30 days after the last TDF/FTC dose, leaving 64 that could have been acquired during drug exposure (0.64/100 pyrs, 95%CI 0.5-0.82). Incidence was similar for men and women. Drug levels were collected at the visit that seroconversion was diagnosed or the prior visit in some of the projects and were available for 49 individuals. Four of the 49 had drug levels compatible with four or more tablets a week. Eleven of 49 had resistance to FTC and one had resistance to tenofovir (TFV).

The World Health Organisation issued a strong recommendation in 2015 that PrEP be included in a comprehensive prevention package made available to populations with substantial incidence, defined as 3/100 pyrs or more[18]. Inclusion of PrEP in national programmes has been slow but steady with success in Kenya particularly with respect to uptake amongst key populations (MSM and female sex workers), although retention has been disappointing[19]. Globally, coverage of PrEP remains very low in relation to estimated need[20]. Even in the US where PrEP has been available since 2012, it is estimated that less than 150,000 of the 492,000 MSM who would benefit from TDF/FTC PrEP have initiated PrEP, and persistent use ranges from 38-57% after 6 months[21,22]. The only country to exceed the target set for uptake of PrEP is Australia where a 25% reduction in population incidence was reported in 2018[23].

1.4 RESEARCH LEADING UP TO THE PROPOSED TRIAL

1.4.1 DNA-HIV-PT123

The DNA-HIV-PT123 HIV vaccine evaluated in this protocol has a DNA plasmid backbone that was developed by the Dale and Betty Bumpers Vaccine Research Center, NIAID, NIH (Bethesda, MD, USA). The CMV/R promoter consists of the translational enhancer region of the CMV immediate early region 1 enhancer substituted with the 5'-untranslated human T-cell leukemia virus type 1 (HTLV-1) R-U5 region of the long terminal repeat (LTR) to optimize gene expression. Other elements of the plasmid include a bovine growth hormone polyadenylation signal termination sequence (Tbgh) and a kanamycin resistance cassette (Kan). The plasmid and host *E. coli* strain used in the production of the vaccine are characterized in accordance with the relevant sections of the FDA guidance "Points to Consider in the Production and Testing of New Drugs and Biologicals Produced by Recombinant DNA Technology" (1985), the "Supplement: Nucleic Acid Characterization and

Genetic Stability" (1992), "Points to Consider in Human Somatic Cell Therapy and Gene Therapy" (1991, 1998), and "Points to Consider on Plasmid DNA Vaccines for Preventive Infectious Disease Indications" (1996). Additional information and references can be found in the Investigator's Brochure (IB).

The DNA-HIV-PT123 HIV vaccine includes three DNA plasmids. One encodes clade C ZM96 Gag, another clade C ZM96 Env, and the third encodes CN54 Pol-Nef. Enhancements made to the inserts include RNA and codon optimization, RNA secondary structures modulation, splice sites removal, TCF binding sites removal, and increasing the GC content.

Nonclinical safety studies of DNA-HIV-PT123

A number of GLP toxicity studies and non-GLP immunogenicity studies in non-human primates have been performed with DNA-HIV-PT123, in combination with poxvirus based vaccines (NYVAC and ALVAC) and/or gp120 protein based vaccine candidates. Details of these studies are summarised in the investigator's brochure of DNA-HIV-PT123.

Clinical studies with DNA-HIV-PT123

DNA-HIV-PT123 has been evaluated in seven phase I clinical trials, and in combination with AIDSVAX® B/E in four of which were conducted in over 200 HIV-negative healthy volunteers[24-27]. Table 2 provides an overview of these trials.

Table 2: DNA-HIV-PT123 Clinical Experience Summary

Protocol	Candidate Vaccines	Trial Sites	Status	# Received DNA
EV04/HVTN096	DNA-HIV-PT123<br>NYVAC-HIV-PT1<br>NYVAC-HIV-PT4<br>AIDSVAX® B/E	Switzerland	Completed	40
HVTN092	DNA-HIV-PT123<br>NYVAC-HIV-PT1<br>NYVAC-HIV-PT4	US and Switzerland	Terminated early due to NYVAC GMP issue	126
HVTN105	DNA-HIV-PT123<br>AIDSVAX® B/E	US	Completed	104
EV06	DNA-HIV-PT123<br>AIDSVAX® B/E	Uganda	Completed	60
EV07<br>(subset of EV06)	DNA-HIV-PT123<br>AIDSVAX® B/E	Uganda	Completed	(49)<br>(of 60 EV06 ppts)
HVTN108	DNA-HIV-PT123<br>Bi-valent gp120+<br>MF59 or $AS01_B$	US<br>South Africa	Fully Enrolled	260
HVTN111	DNA-HIV-PT123<br>Bi-valent<br>gp120+MF59	South Africa<br>Tanzania<br>Zambia	Completed	120
**Total**				**710**

DNA-HIV-PT123 is safe and well tolerated. As with other similar DNA vaccines studied previously, local reactions (such as pain, redness, induration and erythema) and systemic reactions (such as fever, headache, chills, malaise, nausea, myalgia or arthralgia) have been observed. These adverse events were mostly of mild or moderate intensity and transient.

The evidence derived from clinical studies demonstrated that DNA-HIV-PT123 is immunogenic and plays an important role in priming the response when combined with NYVAC and/or protein.

More details of these studies can be found in the IB for DNA-HIV-PT123.

1.4.2 AIDSVAX® B/E

AIDSVAX® B/E is a bivalent HIV gp120 glycoprotein originally developed and manufactured by Genentech Inc. The development and manufacturing rights were subsequently transferred to VaxGen, Inc. and finally transferred to its current developer, Global Solutions for Infectious Diseases (GSID). It is a purified mixture of gp120 proteins produced by recombinant DNA procedures using Chinese hamster ovary (CHO) cell expression. The sequences of MN gp120/HIV-1 and A244 gp120/HIV-1 are expressed as fusion proteins where a 27 amino acid sequence found in the gD protein of herpes simplex virus type 1 is fused to the amino terminus of each protein. MN and A244 rgp120/HIV-1 are combined to produce the bivalent AIDSVAX® B/E vaccine. AIDSVAX® B/E encompasses both subtype B (MN) and subtype E (A244) proteins that are adsorbed onto 600mcg of aluminium hydroxide gel suspension as adjuvant.

Nonclinical safety studies of AIDSVAX® B/E

This product is well characterized pre-clinically and clinically ; please refer to the AIDSVAX®B/E IB for nonclinical safety data.

Clinical studies with AIDSVAX® B/E

AIDSVAX® B/E has been tested in more than ten thousand volunteers, including several phase I/II trials in the US, Switzerland, South Africa, Uganda and Thailand and two phase III trials: VAX003 and RV144 in Thailand[2,7]. Table 3 summarizes the clinical experience of AIDSVAX® B/E in human trials.

AIDSVAX® B/E is safe and well tolerated. In the VAX003 trial, the most common reactogenicity event reported was pain and tenderness at the injection site. Other common reactogenicity events included fatigue, malaise, limited arm movement, myalgia, headache, and injection site oedema. Upper respiratory infection was the most commonly reported adverse event.

Only one severe hypersensitivity reaction has been reported, occurring in RV328, a Phase I/II trial in Thailand, in a participant shortly after receiving the 4th and final dose of AIDSVAX® B/E[28]. Within three minutes of receiving the vaccine, the participant developed a generalized pruritic rash, facial swelling, and malaise, but had no difficulty breathing nor hypotension. This individual had received 3 prior vaccinations with AIDSVAX®B/E with no concomitant symptoms. The participant was treated and recovered completely within a few hours.

In the prime-boost trial, RV144, pain and tenderness, headache, fatigue, arthralgia, and myalgia were the most frequently reported local and systemic reactions. All reactogenicity symptoms resolved rapidly and spontaneously, in the vast majority of cases within three days.

AIDSVAX® B/E is also immunogenic. In the phase III VAX003 study, all volunteers had an immune response, with substantial increases in Antibody titres after each dose given.

For more details, please refer to the AIDSVAX® B/E Investigator's Brochure.

Table 3: AIDSVAX®B/E Clinical Experience Summary

Protocol	Candidate Vaccine(s)	Study Sites	Status	# Received AIDSVAX®B/E
VAX001	AIDSVAX® B/E	Thailand	Completed	92
VAX003	AIDSVAX®B/E	Thailand	Completed	1,250
RV135	AIDSVAX® B/E<br>ALVAC-HIV	Thailand	Completed	97
RV144	AIDSVAX® B/E<br>ALVAC-HIV	Thailand	Completed	8,197

RV305 (RV144 subset)	AIDSVAX® B/E ALVAC-HIV	Thailand	Completed	(90)
RV306	AIDSVAX®B/E ALVAC-HIV	Thailand	Completed	327
RV328	AIDSVAX®B/E	Thailand	Completed	30
HVTN096	AIDSVAX®B/E DNA-HIV-PT123 NYVACHIV-PT1&4	Switzerland	Completed	80
HVTN097	AIDSVAX® B/E ALVAC-HIV	South Africa	Completed	80
HVTN105	AIDSVAX® B/E DNA-HIV	US	Completed	104
HVTN110	AIDSVAX® B/E AD4-ENVC150 AD4-MGAG	US	Completed	20
PXVX-HIV-100-001	AIDSVAX® B/E AD4-ENVC150 AD4-MGAG	US	Ongoing	18
EV06	AIDSVAX®B/E DNA-HIV-PT123	Uganda	Completed	60
EV07 (EV06 subset)	AIDSVAX® B/E DNA-HIV-PT123	Uganda	Completed, Analyses pending	(49)
HVTN114	AIDSVAX® B/E MVA/HIV62B	US Peru	Completed, Analyses pending	27
HVTN121	AIDSVAX® B/E	US	ongoing	16
**Total**				**10,398**

1.4.3 CN54gp140 IN GLA-AF OR MPLA-L

Recombinant CN54gp140 is a HIV-1 envelope protein from the clade C strain 97/CN/54 isolate, which comprises a sequence of 634 amino acids. The C-clade HIV-1 subtype is believed to cause more than 50% of worldwide HIV-1 infections and is predominant in southern and eastern Africa and India. Previously reported nonclinical studies have shown that CN54gp140 is immunogenic in mice, rabbits and macaques when given via the IM, ID or IVAG routes.

MPLA is a non-toxic version of LipoPolySaccharide (LPS), which is isolated from the LPS lipid A region of Salmonella Minnesota R595 and retains the immune-stimulatory properties of LPS, but exhibits low toxicity. The adjuvant effect initiated by MPLA is due to the recognition and binding with Toll-like receptor 4 (TLR4) a family of pattern-recognition receptors, found to be present on the surface of antigen presenting cells such as dendritic cells, monocytes, macrophages and B-cells. TLRs recognise a limited number of conserved elements in pathogens and play a crucial role in the immune response to infection and vaccination. TLR4 agonists induce acute inflammatory responses

including the production of chemokines and cytokines that mediate cell infiltration, as well as stimulating dendritic cell maturation and the induction of adaptive immune responses.

Nonclinical safety studies of CN54gp140 in GLA

A number of GLP toxicity studies and non-GLP immunogenicity studies in rabbits, mice and guinea pigs have been performed with CN54gp140 with or without GLA. Details of each study can be found in the IB for CN54gp140 and MPLA-L.

Clinical safety studies of CN54gp140 in GLA-AF or MPLA-L

CN54gp140 alone or adjuvanted with GLA/MPLA-L (5µ) has been tested at a dose of 100mcg in over 350 volunteers in 6 Phase I clinical studies in Sweden, the UK and in Africa[29-33]. The vaccine has been shown to be safe, non-toxic, and any inflammation that occurred was localised at the site of vaccine administration and similar to local reactions seen with licensed vaccines. Almost all the systemic reactions reported were considered mild or moderate in nature (fever, malaise, myalgia, headache, nausea and vomiting).

There has been one serious adverse event of note. A female participant enrolled in Spoke 3[31] presented to the emergency room within 2 days of vaccination with abdominal pain. She was managed with non-opiate pain killers but kept in overnight due to suspected appendicitis. However it is likely that this was due to a lymphadenitis following immunization.

Evidence from clinical studies has shown that adjuvanted CN54gp140 can boost binding antibody responses and enhance Env specific responses following a DNA prime.

More details of the studies outlined in **Table 4** can be found in the IB for CN54gp140/MPLA-L

Table 4: CN54gp140 adjuvanted with GLA-AF or MPLA-L Clinical Experience Summary

Protocol	Candidate Vaccines	Study Sites	Status	# Received CN54gp140 with GLA-AF/MPLA-L
Mucovac 02	CN54gp140<br>GLA-AF<br>CN54gp140<br>without GLA-AF	UK	Completed	36
CUTHIVAC 02	DNA-CN54-Env<br>CN54gp140<br>GLA-AF	UK	Completed	24
X001	CN54gp140<br>GLA-AF	UK	Completed	12
TaMoVac 01<br>With AfreVacc boost	HIVIS-DNA<br>MVA-CMDR<br>CN54gp140<br>GLA-AF	Tanzania	Completed	35
TaMoVac II	HIVIS-DNA<br>MVA-CMDR<br>CN54gp140<br>GLA-AF	Tanzania<br>Mozambique	Completed	68
HIVIS 07	HIVIS-DNA<br>MVA-CMDR<br>CN54gp140<br>GLA-AF	Sweden	Completed	25
Spoke 3	DNA-CN54 Env<br>MVA-C<br>CN54gp140<br>GLA-AF	UK	Completed	40
AD4HIV	AD4-HIV-<br>CN54Env	UK	Ongoing	>36

	MVA-C CN54gp140-MPLA liposomes			
**Total**				276

1.4.4 MVA-CMDR

Modified Vaccinia Ankara-Chiang Mai Double Recombinant (MVA-CMDR) is a non-replicating, highly attenuated strain of Vaccinia virus that has been genetically engineered to express the HIV-1 genes envgp160 CM235 Subtype E and gag and pol CM240 Subtype A (integrase-deleted and reverse transcriptase non-functional). MVA was generated following 570 passages through chicken embryo fibroblast (CEF), which resulted in Vaccinia losing a significant portion of its DNA, including the genes involved in immune evasion and virulence.[34] Although the virus loses its ability to produce infectious virions, the ability to express foreign sequences is retained. Thus, while the safety profile of MVA is enhanced, it retains its ability to stimulate effective immune responses to foreign antigens. Since its use as a vaccine for small pox in the 1960s, it is now widely used as a vaccine vector for a wide range of infectious diseases.

Nonclinical safety studies of MVA-CMDR

A number of GLP toxicity studies and non-GLP immunogenicity studies in rabbits have been performed with MVA-CMDR. Details of each study can be found in the IB for MVA-CMDR.

Clinical safety studies of MVA-CMDR

MVA-CMDR is safe and well tolerated and has been tested in over 600 healthy HIV negative volunteers in Europe, the US, Asia and Africa[32,35-41]. The most frequent reactogenicity symptoms reported were pain and/or tenderness at the injection site with the majority being mild. Systemic events included fever (axillary temperature >37.5°C), malaise, chills, arthralgia, myalgia, headache, nausea and vomiting. Adverse events were mainly mild or moderate with the exception of three participants in the TaMoVac II trial[32], one of whom reported severe pain, itching and warmth as well as severe chills/rigor, arthralgia, myalgia, headache and nausea following the second MVA-CMDR immunization. The other two severe events in this trial were transient elevated temperature (HIV-MVA/rgp140/GLA-AF) and nausea (HIV-MVA alone).

Evidence from clinical studies has shown that boosting with MVA-CMDR following a DNA prime leads to an increase in immunogenicity. More details of these studies, outlined in **Table 5**, can be found in the IB for MVA-CMDR

Table 5: MVA-CMDR Clinical Experience Summary

Protocol	Candidate Vaccines	Study Sites	Status	# Received MVA-CMDR
RV 158	MVA-CMDR	US<br>Thailand	Completed	40
HIVIS 04	MVA-CMDR	Sweden	Completed	10
HIVIS 02	HIVIS DNA<br>MVA-CMDR	Sweden	Completed	40
HIVIS 05 (HIVIS 02 subset)	MVA-CMDR Given 3 years after the previous dose MVA-CMDR in HIVIS 02	Sweden	Completed	(24)
HIVIS 03	HIVIS DNA<br>MVA-CMDR	Tanzania	Completed	42

HIVIS 06 (HIVIS 03 subset)	Additional boost of MVA-CMDR in participants previously immunized with HIVIS DNA and MVA-CMDR	Tanzania	Completed	(20)
**HIVIS 07**	HIVIS DNA MVA-CMDR	Sweden	Completed	25
**TaMoVac 01**	HIVIS DNA MVA-CMDR	Tanzania	Completed	108
**TaMoVac 01**	HIVIS DNA MVA-CMDR	Mozambique	Completed	20
**TaMoVac II**	HIVIS DNA MVA-CMDR CN54gp140 GLA-AF	Tanzania	Completed	145
**RV262**	PENNYVAX™G DNA MVA-CMDR	Kenya Uganda Tanzania	Completed	74
**RV365**	MVA-CMDR in participants previously immunized with DCVax 001 or poly- ICLC	US	Completed	14
**HVTN 106**	HIV-1 DNA (Nat-B, CONS-S env, Mosiac env) MVA-CMDR	US Switzerland	Completed	90
**Total**				608

1.4.5 SELECTION OF THE COMBINATION VACCINE REGIMENS

The selection of combination regimens for the PrEPVacc trial is based on data from previous clinical studies described above and practical considerations.

The combination of DNA-HIV-PT123 and AIDSVAX® B/E to be studied in PrEPVacc has been evaluated in four phase I/II trials in US, Europe and Uganda. More than 200 healthy volunteers (see **Table 2** above) have received this combination and have shown that the combination is safe and well tolerated. The combination is also shown to be highly immunogenic [24-26]

The TaMoVac 01 trials and TaMoVac II explored the safety and immunogenicity of combining MVA-CMDR and CN54gp140 in GLA-AF to boost HIV negative participants in Tanzania and Mozambique who were primed with a multi-clade heterologous multi-component DNA (HIVIS DNA). This regimen led to potent, broad, balanced T cell and anti-Env antibody responses. HIVIS DNA is not available and so participants in the second active vaccine regimen in PrEPVacc will be primed with the DNA-PT123 in combination with the CN54gp140+MPLA-L.

Both active regimens will follow the same schedule with the two immunisations close together at weeks 0 and 4, followed by a long gap before the third at week 24. Peak cellular responses are not expected until after the third immunisation and based on previous trials such as RV144 and the TaMoVac trials, these will wane over the course of 6 months but resurge after a boost at week 48 or later.

1.4.6 TAF/FTC As PrEP

Emtricitabine (FTC) and Tenofovir alafenamide (TAF) is approved for use as treatment in several jurisdictions, including in at least two of the participating countries. It is also approved for PrEP by FDA except in persons who have receptive vaginal sex. TAF is a pro-drug of tenofovir with rapid and sustained cellular loading, but lower genital tract tissue concentrations compared to TDF/FTC.

Nonclinical and clinical safety studies of TAF/FTC

Oral TAF/FTC has been shown to protect monkeys from vaginal and rectal SHIV challenge, and a large phase III clinical trial assessing the effectiveness as PrEP in HIV negative MSM and transgender women, reported in 2019. There were 7 and 15 new infections in 4370 and 4386 pyrs of follow-up in the TAF/FTC and TDF/FTC groups respectively (incidence rate ratio 0.47, 95% CI 0.19-1.15). This excludes the non-inferiority margin of 1.62, demonstrating that TAF/FTC is at least as effective as TDF/FTC [42].

Tenofovir alafenamide (TAF), a prodrug of tenofovir (TFV), produces higher intracellular concentrations of TFV-diphosphate (TFV-DP), the active metabolite than tenofovir disoproxil fumarate (TDF). As such, TAF is more potent than TDF and is likely to lead to higher antiviral activity in target tissue with less toxicity in others, particularly kidney and bone[43]. Given this improved safety and potency, and that TAF/FTC is already approved for a treatment indication, it is a good candidate for oral HIV PrEP and could provide an effective prevention option.

A phase I pilot pharmacokinetic (PK) study of a single oral dose of TAF (5, 10, or 25 mg) was conducted in 24 healthy seronegative women to characterize the dose-proportionality of TAF and TFV in multiple compartments. Consistent with previous reports in HIV-infected individuals, lower circulating plasma concentrations of TFV and higher intracellular concentrations (PBMCs) of TFV-DP, relative to TDF were observed. After a single dose, however, TFV-DP was unquantifiable in most genital and rectal tissue[44].

A study of 14 days oral dosing of TAF/FTC compared to TDF/FTC was conducted in 72 healthy, HIV seronegative women to determine safety and PK profiles after multiple doses. Initial data were presented at the HIV Research for prevention (R4P) 2018 conference and found fewer gastrointestinal side effects, lower plasma TFV concentrations and higher TFV-DP in PBMCs with TAF/FTC compared to TDF/FTC. Of note, although TFV-DP was unquantifiable in many genital tissue samples, there were higher cervico-vaginal tissue TFV-DP concentrations at 4hrs with TAF/FTC compared to TDF/FTC and protection against HIV was demonstrated in an ex-vivo tissue infection model[45].

Please refer to the Summary of Product Characteristics (SPC) section 4.8 for more information on the safety profile of this drug.

1.5 OBJECTIVES AND RATIONALE FOR PROPOSED TRIAL

1.5.1 Objectives

The objectives of this Phase IIb clinical trial are:

1) To determine the vaccine efficacy of each combination HIV vaccine regimen compared to placebo, in preventing acquisition of HIV infection
2) To determine the effectiveness of TAF/FTC PrEP as a function of the effectiveness of TDF/FTC PrEP and hypothetical placebo
3) To determine the safety of (i) each combination HIV vaccine regimen compared to placebo and (ii) TAF/FTC PrEP in comparison to TDF/FTC PrEP
4) To understand the trial results in the context of risk and adherence behaviours, knowledge attitudes and perceptions of participants, study staff and the wider community

1.5.2 RATIONALE

PrEPVacc is a phase IIb, three arm, two stage HIV-1 prophylactic vaccine trial with a concurrent randomisation to compare TAF/FTC to TDF/FTC as PrEP. The design is efficient as two different combination vaccine regimens will be evaluated, as well as an experimental PrEP agent, and pragmatic in that it mirrors the logical way that PrEP would be used if these regimens were to proceed into a public health programme, supporting the vaccine schedule through to optimal immunogenicity 2 weeks after the third immunisation.

The trial is designed to detect vaccine efficacy of ***public health relevance***. In light of the high level of effectiveness demonstrated in the PrEP trials (up to 86% reduction in HIV incidence) and in programmes (near perfect protection), the investigators consider that an HIV vaccine regimen would have to observe a reduction in HIV incidence of around 70% to justify implementation. Although this is higher than seen previously, the reduction in HIV in RV144 during the first 12 months of follow-up was 60% suggesting higher vaccine efficacy is possible. With a target vaccine efficacy of 70% reduction HIV, an observed reduction of 47% would be statistically significant in the final trial analysis.

The assumptions underlying the sample size calculation for the vaccine trial include an HIV incidence of 4/100 person years. Although there is evidence from previous studies and surveillance data to support this high incidence across the totality of the participating centres, there is considerable uncertainty. Incidence is generally declining in the region as testing expands, treatment is started promptly and access to PrEP increases. In order to determine the sample size for each centre, to refine the recruitment strategy and facilitate timely enrolment in the trial, enrolment in a Registration Cohort began in June 2018 under separate protocols in each centre.

Rationale for the proposed vaccination schedule

The vaccination schedule in the original RV144 trial included ALVAC®HIV vaccine vCP1521 administration at time 0 and Month 1 and ALVAC® HIV vaccine vCP1521+ AIDSVAX® B/E at Months 3 and 6. The vaccine efficacy (VE) observed in the first year approached 60%, and waned over the 3.5 years of follow-up to an overall VE of 31%.

Building on the RV144 schedule and results, we have altered the PrEPVacc vaccination schedule with the aim of improving the magnitude and durability of vaccine-elicited immune responses beyond those observed in RV144. These changes have been made under the scientific assumption that improvements of immune responses will translate into improvements in vaccine efficacy. These alterations include:

1) Introduction of a protein vaccine from the beginning as antibody responses were not detected for the first 12 weeks in the RV144 trial which did not include the combination of ALVAC/AIDSVAX® B/E until week 12. Several phase I trials[24,25,46] have clearly shown that co-administration is well-tolerated and results in earlier induction and higher magnitude and durability of protective V1V2 antibody responses over 18 months[24]. The EV06 trial evaluated co-administration of DNA-HIV-PT123 with AIDSVAX® B/E protein at week 0, 4 and 24, as proposed in PrEPVacc, and showed the induction of potent binding and neutralizing antibody responses[25]. A more recent analysis of the EV06 IgG binding antibody responses against a multi-clade panel of V1V2 constructs performed by G. Tomaras group from Duke University, revealed potent cross clade responses (G. Tomaras, personal communication).
2) Addition of the boost at week 48 is expected to prolong the durability of the peak immunogenicity compared to RV144.

Rationale for the evaluation of TAF/FTC as PrEP

TDF/FTC is very safe, but renal and bone toxicity are concerns with long term use, especially as individuals age and acquire co-morbidities that could aggravate these toxicities. TAF/FTC requires a

smaller concentration of tenofovir than TDF/FTC with two advantages: reduced toxicity and reduced manufacturing costs. In light of the reduced costs, it is likely that TAF/FTC will replace TDF/FTC in settings where generic drugs are used. The DISCOVER trial compared TAF/FTC to TDF/FTC in MSM and transgender women in North and South America and Europe observing only 7 infections in the 2,694 TAF/FTC participants compared to 15 in the 2,693 TDF/FTC participants[42]. Although regulatory authorities may feel able to extrapolate the results to heterosexual men, this is unlikely to be the case for women due to the differences in tissue penetration. The lack of clinical effectiveness data for TAF/FTC when used as PrEP in heterosexual populations, particularly women, could be a critical gap if TAF/FTC replaced TDF/FTC as the main drug in Sub-Saharan Africa and other settings where the dominant transmission route is heterosexual sex.

Ethical considerations in the provision of PrEP in prevention trials

TDF/FTC PrEP has been strongly recommended by the World Health Organisation as a risk reduction strategy to be provided to populations with substantial risk of acquiring HIV since 2015. Countries continue to explore how to integrate PrEP through demonstration projects and national programmes, including , South Africa, Tanzania and Uganda, and have included PrEP in their national policies. Kenya is one of the most advanced countries in the world with respect to implementation of PrEP, with between 33-44% of eligible individuals from key populations (men who have sex with men and female sex workers respectively) initiating PrEP in a national programme. However, much smaller proportions return for more PrEP at three months[19] and it is not yet entirely clear why although concerns about side-effects and stigma, and low perceived need of additional risk reduction strategies, which are potentially life-long, all contribute. The greatest efforts to generate market demand are in the US, where PrEP is widely advertised on social media and even television. Nonetheless penetration is less than 10% of those estimated to be in need according to the US Centre for Disease Control, and again retention in PrEP programmes is low with a median of 8 months in a recently reported study in San Francisco. This is explained in part by the co-payments required by PrEP users, even when the drug itself is free. In England, PrEP has been available free of charge since March 2020.

Guidelines are now clear regarding the provision of TDF/FTC or TDF/3TC (collectively referred to as TD/XTC) PrEP in prevention trials[47], with two recommended approaches to ensure provision of PrEP:

1) TD/XTCX can be the control arm
2) TD/XTCX is provided to all participants for the duration of the trial in the study clinic or through referral to locally available sources

We have chosen to evaluate vaccines and PrEP in a way that a national HIV vaccine programme would be likely to combine the two with daily study PrEP to support the time to peak immune responses and thereafter available to all who wish to continue. We recognise the potential for a conflict of interest for staff when promoting PrEP in a vaccine trial and hope including PrEP as a study drug will address this, and that uptake and adherence will be higher as a consequence, potentially overcoming misgivings that participants may have had about taking PrEP when they enrolled. The data gathered in the preceding Registration Cohort has assisted in mobilising PrEP services, and therefore it is anticipated that investigators will be able to support participants motivated to continue PrEP after they complete the first three immunisations through access in the community or direct provision to a sustainable supply. The Registration Cohort has confirmed that new HIV infections occur even when PrEP is available, particularly in female participants, underscoring the need for other interventions to prevent HIV.

2 SELECTION OF SITES/CLINICIANS

The trial Sponsor has overall responsibility for site and investigator selection, advised by the Trial Steering Committee.

2.1 SITE/INVESTIGATOR INCLUSION CRITERIA

To participate in the PrEPVacc trial, investigators and clinical trial sites must fulfil a set of basic criteria that have been agreed by the PrEPVacc Trial Management Group (TMG) and are defined below. Only sites that have participated in the Registration Cohort and built sufficient capacity can participate in the PrEPVacc trial.

2.1.1 Principal Investigator's Qualifications & Agreements

1. The investigators should be qualified by education, training, and experience to assume responsibility for the proper conduct of the trial at their site and should provide evidence of such qualifications through an up-to-date curriculum vitae and/or other relevant documentation requested by the Sponsor, the REC, the IRB, and/or the regulatory authority(ies).
2. The investigators should be thoroughly familiar with the appropriate use of the investigational products as described in the protocol, in the current Investigator Brochure, in the product information and in other information sources provided by the Sponsor.
3. The investigators should be aware of, and should comply with, the principles of GCP and the applicable regulatory requirements. A record of GCP training should be accessible for all investigators.
4. The investigators/site should permit monitoring and auditing by the Sponsor, and inspection by the appropriate regulatory authorities
5. The investigators should maintain a delegation log of appropriately-qualified persons to whom the Principal Investigator has delegated significant trial-related duties.
6. The Principal Investigator should sign a Principal Investigator statement, which verifies that the investigators at their site are willing and able to comply with the requirements of the trial.

2.1.2 Adequate Resources

1. The investigators should be able to demonstrate a potential for recruiting the required number of suitable subjects within the agreed recruitment period.
2. The investigators should have sufficient time to properly conduct and complete the trial within the agreed trial period.
3. There should be available an adequate number of qualified staff and adequate facilities for the foreseen duration of the trial to conduct the trial properly and safely.
4. The Principal Investigator should ensure that all persons assisting with the trial are adequately informed about the protocol, the investigational product(s), and their trial-related duties and functions.
5. The site should have sufficient data management resources to allow prompt data entry and return of resolved queries to MRC/UVRI and LSHTM Uganda Research Unit (refer to the Data Management Plan for timelines).

2.1.3 Site Assessment

Each selected clinical trial site must complete the PrEPVacc Evaluation Form. The Investigator Statement verifies that the site is willing, and able to comply with the requirements of the trial. This will be signed by the Principal Investigator at the site. In addition and in compliance with the principles of GCP, all site staff participating in the trial must complete the Signature and Delegation of Responsibilities Log and forward this to MRC/UVRI and LSHTM Uganda Resesarch Unit. MRC/UVRI and LSHTM Uganda Research Unit must be notified of any changes to trial personnel and/or their responsibilities.

2.2 SITE/INVESTIGATOR EXCLUSION CRITERIA

It is necessary to recruit a trial population with an HIV incidence in the region of 4/100 person years. Participation will be determined by the PrEPVacc Trial Steering Committee on review of the incidence in the Registration Cohort and sites with low incidence will not be able to take part in the PrEPVacc trial. The target sample size for each site will be determined by the incidence observed in the Registration Cohort or equivalent studies.

2.3 APPROVAL AND ACTIVATION

On receipt of the above essential documents, written confirmation will be sent from MRC/UVRI and LSHTM Uganda Research Unit to the Principal Investigator (PI), and study specific training will be organised. This will include training on the database, randomisation and unblinding procedures as well as the management and reporting of adverse events. The coordinating team will also visit the site pharmacist to go through the pharmacy procedures and documents.

When everything is in place, including a completed Delegation Log the site will be activated and vaccines and drugs dispatched to the named pharmacist in the Accreditation form documents.

1. The site should conduct the trial in compliance with the protocol as agreed by the Sponsor, by the regulatory authority (ies), and by the REC and institutional IRB that gave a favourable opinion.
2. The PI or delegate should document and explain any deviation from the approved protocol, and communicate this with the trial team at MRC/UVRI and LSHTM Uganda Research Unit

3 SELECTION OF PARTICIPANTS

There will be **no exceptions** to eligibility requirements at the time of randomisation. Questions about eligibility criteria should be addressed prior to attempting to randomise the participant. Additional guidance can be found in the Study Operations Manual (SOM) and CRF, for a list of highly effective contraception and what would put volunteers who have systemic disease at screening or enrolment at risk, preclude vaccination or compromise immune responses.

The eligibility criteria are the standards used to ensure that only medically appropriate participants are considered for this study. Participants not meeting the criteria should not join the study. For the safety of the participants, as well as to ensure that the results of this study can be useful for making decisions regarding other participants with similar needs, it is important that no exceptions be made to these criteria for admission to the study.

Participants will be considered eligible for enrolment in this trial if they fulfil all the inclusion criteria and none of the exclusion criteria as defined below.

3.1 PARTICIPANT INCLUSION CRITERIA

1. HIV uninfected adults aged between 18 and 40 years on the day of screening
2. Willing and able to provide informed consent prior to participation
3. Willing and able to comply with the visit schedule and provide blood, urine and other samples at the required time points
4. Home address accessible for visiting and intending to remain within the recruitment area for at least 82 weeks from screening
5. Likely to be at risk from exposure to HIV during follow up
6. Willing to undergo HIV testing, receive HIV test results and risk reduction counselling which includes promotion of PrEP and condoms
7. If female, of child-bearing age and not sterilised, willing to use a highly effective method of contraception from screening until 18 weeks after the last injection
8. If male and not sterilised, willing to avoid impregnating female partners from screening until 18 weeks after the last injection

3.2 PARTICIPANT EXCLUSION CRITERIA

1. HIV infection or indeterminate HIV result at screening or enrolment
2. Hepatitis B surface antigen positive
3. If female, currently pregnant (evidence from positive serum or urine pregnancy test), or lactating
4. Participating in another biomedical research study or in receipt of a live vaccine within 30 days prior to randomisation
5. Participation in a previous HIV vaccine or HIV immunotherapy trial
6. Receiving blood products or immunoglobulins within 12 weeks of screening
7. Known hypersensitivity to any component of the vaccine formulations used in this trial or history of severe or multiple allergies to vaccines, drugs or pharmaceutical agents
8. Presence of a systemic disease at the time of randomisation or history of chronic illness that in the opinion of the investigator may compromise the participant's safety, preclude vaccination or compromise an immune response to vaccine

9. Abnormalities in routine laboratory parameters (Hb, creatinine, AST/ALT, alkaline phosphatase, total Bilirubin and glucose) of Grade 2 and above using the DAIDS toxicity table, version 2.1 July 2017 or estimated glomerular filtration rate less than 60ml/min

3.3 NUMBER OF PARTICIPANTS

The target accrual for the final vaccine analysis is a minimum of 1668 HIV uninfected adults aged 18 – 40 years from participating centres in sub-Saharan Africa, but this is an endpoint driven MAMS trial design and therefore the target may be adjusted following a recommendation from the IDMC (**Section 9.4**). Also, if a participant stops trial follow-up before they have completed the first 3 injections, another participant will be randomised whilst there is sufficient product and provided enrolment is ongoing.

3.4 CO-ENROLMENT GUIDELINES

Participants should not be participating in any other biomedical research study within 30 days prior to randomisation, nor should they co-enrol into any other interventional studies that might impact on the primary or secondary outcomes for the duration of their participation in the PrEPVacc trial.

3.5 SCREENING PROCEDURES & PRE-RANDOMISATION INVESTIGATIONS

Written or witnessed informed consent to enter into the trial and be randomised must be obtained from participants, after explanation of the aims, methods, benefits and potential hazards of the trial and BEFORE any trial-specific procedures are performed or any blood is taken for the trial (see Template Participant Information Sheet and Consent Form). Consent will be taken for participation in any sub -studies (including additional sample storage).

It must be made completely and unambiguously clear that the participant is free to refuse to participate in all or any aspect of the trial, at any time and for any reason, without incurring any penalty or affecting their treatment in future.

Signed consent forms must be kept by the investigator and documented in the case report form (CRF) and a copy offered to the participant. If the participant does not wish to take the copy, this should be documented in their clinic notes and the copy should be filed with the investigator copy.

See **section 6.2** for details of screening procedures.

4 REGISTRATION & RANDOMISATION

Before randomisation, the participant's eligibility must be confirmed by completing the screening CRFs. All blood tests must be available to confirm that the participant is eligible in terms of laboratory criteria.

4.1 RANDOMISATION PRACTICALITIES

Further details on the process of randomisation can be found in **Sections 6.3 and 9.1**.

RANDOMISATIONS
To randomise,
Details to be added when available

Randomisation will be web-based with a manual randomisation process to cover any instances when access to the internet is not possible. This will be detailed in the SOM. In brief, a single code per participant will be generated electronically to inform pharmacy which vaccine group the participant is allocated to, and which PrEP allocation (TAF/FTC or TDF/FTC).

4.2 RANDOMISATION CODES & UNBLINDING

Randomisation codes and unblinding are considered in **Section 5.6.**

Study staff and participants will not know who has been allocated vaccine and who has been allocated placebo, but it is not possible to blind staff to the difference in volume as they will be administering 1ml in one arm and 0.45ml in the other at the same timepoint on four occasions (see section 5.3.6). Study staff will therefore be able to identify who has been randomised to the schedule that includes gp140 in MLPA-L or its matched placebo.

Allocation to the vaccine regimens will be in the ratio of 1:1:1 for groups A:B:C respectively. As the volume of the recombinant proteins differs between the two active arms, the placebo injection will be further divided 1:1 to 1ml: 0.45ml in order to ensure that staff administering the injection cannot differentiate between active and placebo.

As the trial is adaptive, the ratio may change to 1:1 during the trial if one of the vaccine regimens is discontinued or temporarily paused due to an issue with one of the products. If this is necessary, a new randomisation database will be implemented with the new list and the previous database archived after completing the quality checks.

The pharmacist at each centre will know the precise allocation throughout the trial as they will have a list that is mapped to each randomisation list

The randomisation to control PrEP:experimental PrEP is 1:1 and all study staff and participants will know the allocation after randomisation as this is is open-label.

5 TREATMENT OF PARTICIPANTS

5.1 INTRODUCTION

The trial treatments in this trial are two different active combination HIV vaccine regimens, using products that are not licensed, saline placebo, and two different oral PrEP drugs both of which are licensed for treatment of HIV infection, and for prevention in some populations in some countries.

The vaccine products will need to be drawn up by the pharmacist who will have access to a list matching the trial identifier to the allocated regimen. Two vaccine products will be administered at each of four timepoints. They will be injected intramuscularly into the deltoid muscle of each upper arm, with DNA, MVA or matched placebo in the left arm and recombinant protein or matched placebo in the right arm.

The PrEP tablets will be dispensed by study staff in an open-label manner at three to four timepoints, and additional timepoint to top up supply if required.

The treatment schedule is outlined in table 6 below:

Table 6: Vaccine and PrEP candidates, placebo and PrEP control, and dispensing Schedule

**Visit Schedule**<br>**Target week**	**Visit 2**<br>**Week 0**	**Visit 4**<br>**Week 4**	**Visit 7**<br>**Visit 4 +12wks**	**Visit 8**<br>**Visit 4 + 20wks**	**Visit 12**<br>**Visit 8 + 24 wks**
**Group A**<br>Vaccine combination	DNA-HIV-PT123<br>AIDSVAX® B/E	DNA-HIV-PT123<br>AIDSVAX® B/E		DNA-HIV-PT123<br>AIDSVAX® B/E	DNA-HIV-PT123<br>AIDSVAX® B/E
**Group B**<br>Vaccine combination	DNA-HIV-PT123<br>CN54gp140 +MPLA-L	DNA-HIV-PT123<br>CN54gp140 +MPLA-L		MVA<br>CN54gp140 +MPLA-L	MVA<br>CN54gp140 +MPLA-L
**Group C**<br>Placebo control	N/saline	N/saline		N/saline	N/saline
**PrEP Candidate**<br>TAF/FTC	60 tablets	90 tablets	+/-30 tablets	If required	
**PrEP Control**<br>TDF/FTC	60 tablets	90 tablets	+/-30 tablets	If required	

Additional detail regarding the treatments to be given in this trial is provided in the IBs for unlicensed products or SPC for licensed products, the study SOM and Local Pharmacy Working Instructions.

5.2 COMBINATION GROUP A

5.2.1 DNA-HIV-PT123

DNA-HIV-PT123 candidate HIV vaccine is released by Ajinomoto Althea Inc, San Diego, US in accordance with GMP and provided by the EuroVacc Foundation, Lausanne, Switzerland.

DNA-HIV-PT123 is supplied as a 4 mg/mL DNA solution in a 2 mL sterile glass vial containing a volume to deliver 1 ml of a clear, colourless, sterile isotonic solution. Each vial is in an individual secondary packaging. Vials and packaging are labelled according to GMP.

5.2.2 AIDSVAX® B/E

The AIDSVAX®B/E vaccine was originally developed by Genentech, Inc., and is now being provided by Global Solutions for Infectious Diseases.

AIDSVAX® B/E is supplied as a sterile suspension in single-use glass vials containing a volume to deliver 1mL of MN rgp120 and A244 rgp120/HIV protein (300mcg each) adsorbed onto a total of 600 mcg aluminium as aluminium hydroxide gel suspension as adjuvant. Vials and packaging are labelled according to GMP.

5.2.3 Treatment Schedule

The target is a minimum of 556 individuals who complete the schedule, and so participants who drop out before the third injection timepoint will be replaced by randomising another eligible volunteer. Therefore up to 620 individuals could receive this combination.

Participants randomised to Schedule A will receive DNA-HIV-PT123 vaccine and AIDSVAX® B/E protein as described in **Table 6** at visits 2, 4, 8 and 12.

5.2.4 Dose and Administration

1ml of DNA-HIV-PT123 will be injected into the deltoid muscle of the left upper arm using the appropriate needle and in accordance with the SOM. Study staff should inject the same arm on each of the four occasions.

1ml of AIDSVAX® B/E will be injected into the deltoid muscle of the right arm using the appropriate needle and in accordance with the SOM. Study staff should inject the same arm on each of the four occasions.

In the event that study staff administer the product into the opposite muscle to the one intended a deviation should be noted (see SOM for details) and revert to the correct arm for any remaining vaccinations.

5.2.5 Storage

The vaccines will be stored at the following conditions according to the SOM and Local Pharmacy Working Instructions that will be reviewed at site initiation. They will be stored in a secure, limited access storage area under the specified storage requirements:

- DNA-HIV-PT123 must be stored at -20°C±5°C.
- AIDSVAX® B/E must be kept refrigerated (2° to 8°C).

5.2.6 Dispensing

The PI will ensure that the vaccines are dispensed in accordance with the protocol, the study specific SOM and Local Pharmacy Working Instructions as appropriate. The study pharmacist of the respective site will be responsible for preparing the injection syringes in accordance to the SOM. The syringe will be labelled with “Name of Study Product/placebo” with an overlay to maintain blinding (detailed label design will be defined in the SOM). Local Pharmacy Working Instructions will be reviewed at site initiation.

Accountability Logs will be kept to record the identification of the participant to whom the vaccine was dispensed and the date. Any damaged or unused vials will also be documented. The log will be checked during monitoring visits and at the end of the trial.

5.2.7 Dose Modifications & Interruptions

There are no planned modifications to dose, other than interruption and discontinuation (see **sections 5.7 and 9.4** for details of discontinuation). This applies to all of the vaccine products.

The schedule may be interrupted if a participant has symptoms or signs on the day of scheduled injection, and the investigator considers it best to defer the injection. Such a decision should be taken in consultation with the PI. The participant will be asked to return for review within the window period

of the scheduled injection. Provided the injection is administered during the window period outlined in the paragraph below for missed visits, this will not be a protocol deviation.

The participant may not be well enough within the ideal window period, or may not be able to attend in this timeline for reasons outside their control. Study staff should consult the SOM to find out whether they can proceed with the injection or need to contact the TSG by email (PrEPVacc_trial_safety@mrcuganda.org). The site PI should consult with the TSG (see **section 14.2**) regarding further injections and the schedule to follow thereafter. Protocol deviations will be recorded in the appropriate CRF.

In the event that there is a confirmed (1) grade 3 or worse solicited adverse event that has persisted for more than 72 hours, regardless of relationship or (2) other grade 3 or worse adverse event that is possibly, probably or definitely related to vaccine, the PI or deputy should interrupt the vaccine schedule and inform MRC/UVRI and LSTHM within 24 hours using the TSG email (see **section 7**). The event will be reviewed by the TSG for an opinion regarding resuming the vaccine schedule, pending further investigation of the event. It may be necessary to consult an independent clinical expert with expertise in the disease area relevant to the adverse event. Such an interruption will be considered a protocol-planned interruption.

As this is a trial with combination vaccine regimens, one of the active regimens may need to be interrupted temporarily if there is a quality issue with any one product that requires further investigation. Enrolled participants will not receive any further injections with this product, and the matched placebos will be managed in the same way in order to maintain the blind between active/placebo. Participants will be informed verbally at the next visit, and in writing after approval from the appropriate ethics committee. If the product under investigation is required at enrolment in one combination arm only, a second randomisation database and list will be implemented to allow enrolments to continue to the other combination arm and placebo in a 1:1 ratio. Once resolved and the other combination arm and matched placebo can be resumed, a third randomisation database and list will be implemented to return to a 1:1:1 ratio. If the product under investigation is DNA-HIV-PT123, all enrolments will need to be paused.

5.3 COMBINATION GROUP B

5.3.1 DNA-HIV-PT123

See **section 5.2.1**.

5.3.2 MVA-CMDR

MVA-CMDR is developed by MHRP (United States Military HIV Research Program), and manufactured by Vibalogics GmbH in Germany according the European Union regulations for GMP.

MVA-CMDR is presented as a frozen liquid formulation, which is thawed prior to injection and appears as a white to opaque, slightly to moderately cloudy liquid.

The vaccine is provided in sealed glass vials containing $1x10^8$ pfu/mL of product. Vials and packaging are labelled according to GMP.

This vaccine candidate is classified as a genetically modified organism (GMO) in South Africa and will be used in compliance with the applicable national regulations.

5.3.3 CN54gp140

CN54gp140 is a recombinant envelope protein derived from a clade C HIV-1 isolate. CN54gp140 is manufactured by Polymun Scientific Immunbiologische Forschung GmbH, Klosterneuburg, Austria according to European GMP.

CN54gp140 drug product is a colourless soluble glycoprotein with no visible particulates. The vaccine is provided in Type I glass vials (3 ml) with a nominal concentration of 500µg/ml and containing a volume of 0.35ml. Vials and packaging are labelled according to GMP.

5.3.4 MPLA Liposomes (MLPA-L)

Monophosphoryl Lipid A (MPLA), a widely used adjuvant, is a non-toxic version of LipoPolySaccharide (LPS), which is isolated from the LPS lipid A region of Salmonella Minnesota R595. In this study, MPLA is co-formulated with liposomes.

MPLA is purchased from Avanti Polar Lipids and formulated by Polymun in liposomes using cholesterol, DMPC and DMPG according to GMP. The MPLA-L formulation is presented as a milky white to opalescent suspension.

MPLA-L drug product is filled at a a nominal concentration of 25µg/ml into Type I glass vials (3 ml)with fluoropolymer coated chlorobutyl rubber stoppers and flip-off plain aluminium overseals. Vials and packaging are labelled according to GMP.

5.3.5 Treatment Schedule

The target is a minimum of 556 individuals who complete the schedule, and so participants who drop out before the third injection timepoint will be replaced by randomising another eligible volunteer. Therefore up to 620 individuals could receive this combination.

Participants randomised to Schedule B will receive DNA-HIV-PT123 vaccine and CN54gp140 combined with MPLA-L as described in **Table 6** at visits 2 and 4 and MVA vaccine and CN54rgp140 combined with MPLA-L at visits 8 and 12.

5.3.6 Dose and administration

1ml of DNA-HIV-PT123will be injected into the deltoid muscle of the left upper arm using the appropriate needle and in accordance with the SOM. Study staff should inject the same arm on each of the two occasions.

1ml ($1x10^8$ pfu) of MVA will be injected into the deltoid muscle of the left upper arm using the appropriate needle and in accordance with the SOM. Study staff should inject the same arm on each of the two occasions, and this should be the arm in which DNA-HIV-PT123 was administered.

In the event that study staff administer the product into the opposite muscle to the one intended a deviation should be noted (see SOM for details) and revert to the correct arm for any remaining vaccinations..

The pharmacist will withdraw 0.4mL of MPLA-L from the 3ml vial containing 0.55mL of MPLA-L and add this to the 3ml vial containing 0.35mL of CN54gp140. The vial contents will be mixed by gentle agitation and 0.45mL of will be withdrawn from the vial to make a concentration of 100µg CN54gp140 and 5µg MPLA-L and provided to study staff in a syringe in accordance with the SOM. The combined products will be injected into the deltoid muscle of the right arm using the appropriate needle and in accordance with the SOM. Study staff should inject the same arm on each of the four occasions.

As the volume of the CN54gp140 in MPLA-L is only 0.45ml and this differs from the volume being injected into the other arm(1ml), clinic staff will be aware that participants have been randomised to this arm or matched placebo.

5.3.7 Storage

The vaccines will be stored according to the SOM and Local Working Instructions that will be reviewed at site initiation. They will be stored in a secure, limited access storage area under the specified storage requirements:

- DNA-HIV-PT123 must be stored at -20°C ±5°C.

- MVA-CMDR: must be stored at -80°C ±10°C
- CN54gp140: must be stored at 2-8°C.
- MPLA-L: must be stored at 2-8°C

5.3.8 DISPENSING

See **section 5.2.6**.

5.3.9 DOSE MODIFICATIONS & INTERRUPTIONS

See **section 5.2.7**.

5.4 GROUP C

5.4.1 NORMAL SALINE PLACEBO

Sodium Chloride (NaCl) for injection, 0.9% will be used to draw up the placebo for each vaccine product. The volume will be matched to the vaccine at 1ml for DNA-HIV-PT123, MVA-CMDR and AIDSVAX® B/E, but 0.45ml for CN54gp140 in MPLA-L.

5.4.2 TREATMENT SCHEDULE

The target is a minimum of 556 individuals who complete the schedule, and so participants who drop out before the third injection timepoint will be replaced by randomising another eligible volunteer. Therefore up to 620 individuals could be randomised to receive placebo. As the volume of the CN54gp140 in MPLA-L is only 0.45ml and this differs from the other active vaccine volumes of 1ml, pharmacists will cover the syringe with the label so that although the individual administering the injection can determine the volume by size of syringe or weight they cannot tell if it is active or placebo.

Participants randomised to Schedule C will receive two placebo vaccines as described in **Table 6** at Visits 2, 4, 8 and 12.

5.4.3 DOSE & ADMINISTRATION

The volume of normal saline drawn up will match the vaccine. The 556 participants in Group C will be randomly divided in a 1:1 ratio to receive 1ml in each arm at the four timepoints or 1ml in the left arm and 0.45ml in the right arm at the four timepoints.

In the event that study staff administer the product into the opposite muscle to the one intended a deviation should be noted (see SOM for details) and revert to the correct arm for any remaining vaccinations.

5.4.4 STORAGE

Normal saline will be stored according to the SOM and Local Working Instructions in a secure, limited access storage area under the specified storage requirements:

- Placebo (NaCl): ambient temperature (15-25°C)

5.4.5 DISPENSING

See **section 5.2.6**.

5.4.6 DOSE MODIFICATIONS & INTERRUPTIONS

See **section 5.2.7**.

5.5 PREP DRUGS

5.5.1 TDF/FTC

The drug to be used as PrEP control is TDF/FTC, which is approved for the treatment of HIV infection in all participating countries and for PrEP in a smaller number. Each tablet contains 245mg of tenofovir disoproxil (TDF) and 200mg of emtricitabine (FTC), both of which are nucleot/side analogue HIV-1 reverse transcriptase inhibitors. The drug will be manufactured and supplied by Gilead Sciences, Inc from clinical trial supply. It will be labelled for clinical trial use.

5.5.2 TAF/FTC

The drug to be used as experimental PrEP is TAF/FTC, which is approved for the treatment of HIV in Tanzania and Uganda. Each tablet contains 25mg of tenofovir alfenamide (TAF) and 200mg of emtricitabine (FTC), both of which are nucleot/side analogue HIV-1 reverse transcriptase inhibitors. The drug will be manufactured and supplied by Gilead Sciences, Inc from clinical trial supply. It will be labelled for clinical trial use.

5.5.3 Dose & administration

The dose is one tablet daily and administration is oral.

5.5.4 Storage

The product should be stored in the original packaging at ambient temperature and protected from moisture.

5.5.5 Dispensing and Returns

The PI will ensure that the tablets are dispensed in accordance with the protocol, the study specific SOM and Local Pharmacy Working Instructions as appropriate. Local Working Instructions will be reviewed at site initiation.

PrEP will be dispensed at visits 2, 4 and 7 and interim visits if required to maintain the supply through to 2 weeks after the third immunisation. Participants will be provided with 60 and 90 tablets respectively at visits 2 and 4 as they may not be able to attend the next dispensing visits for reasons outside their control. At visit 7 there will be a reconciliation of tablets and sufficient to last until visit 9 provided (see **Table 6**).

If visit 8 takes place later than 24 weeks from enrolment, participants may require an additional 14 days of PrEP to be dispensed at this visit to cover the period to peak immunogenicity.

A PrEP Accountability Log will be kept to record the identification of the participant to whom the PrEP control and the experimental PrEP was given, the dates they received drug and the amount dispensed. Any damaged or unused tablets that are returned will also be documented. The log will be checked during monitoring visits and at the end of the trial.

5.5.6 Dose Modifications & Interruptions

PrEP must be interrupted if a participant suffers a serious adverse event that could be a drug reaction (see **section 7.1** and **Table 8** for definitions), or if their estimated glomerular filtration rate is confirmed to be <50ml/min using the Cockroft-Gault formula with no alternative clinical explanation, pending further investigation or specialist referral.

The risk of renal toxicity from the drug needs to be weighed against the risk of acquiring HIV and study staff should seek advice from the TSG (see section 14.2) and have an informed discussion with the participant to enable them to decide whether PrEP can be reintroduced, potentially at a modified schedule of alternate days or 4 days a week.

TDF/FTC PrEP does not need to be interrupted during pregnancy, but the benefit of preventing HIV transmission in pregnancy needs to be weighed against the risk to the foetus and study staff should have an informed discussion with the participant to enable her to decide whether to continue PrEP.

There is insufficient information on TAF/FTC use in pregnancy and therefore this drug would need to be switched to TDF/FTC in the event of pregnancy.

5.6 UNBLINDING / UNMASKING

The vaccine component of PrEPVacc is placebo-controlled. The allocated treatment group will be blinded by the randomisation code. Neither the participant nor the investigator will know whether the participant has been allocated the active vaccine or the matched placebo, although staff administering the injection will be able to differentiate between 1ml and 0.45ml which would reveal those allocated to Group B or matched placebo. They will not know whether the product was active or placebo. Information on the allocation will be held centrally and locally as the vaccines and placebo are prepared locally by the pharmacist.

It may be necessary to unblind the allocation for a clinical reason during the working day or in an emergency. Participants will carry a contact card with the name and contact details for clinical staff at their centre, and an emergency number. Information on emergency unblinding can be found in the Safety Management Plan (SMP), held locally.

There may be a need to unblind the monitor for the purpose of ensuring the integrity of the randomisation. Unblinded data for this purpose should only be accessible to the monitor and provided by those who are preparing the reports by allocation for the Independent Data Monitoring Committee (see **section 9.4**). The blind shall be maintained for persons responsible for the ongoing conduct of the study.

Every effort will be made to inform participants which vaccine group they were allocated to at the end of the study.

The PrEP component is open-label and participants and investigators will know which of the two PrEP agents they have been allocated.

5.7 PROTOCOL TREATMENT DISCONTINUATION

In consenting to the trial, participants are consenting to receive vaccines, an offer of PrEP, to attend the visits for trial follow-up and to provide information that supports data collection.

Participants are not obliged to start PrEP or receive any vaccines.

An individual participant may stop injections early or be stopped early for any of the following reasons:

- Pregnancy in the participant
- HIV infection, confirmed by the local laboratory
- Unacceptable toxicity that precludes further injections
- Intercurrent illness that prevents further injections including emergent conditions that meet the exclusion criteria
- Withdrawal of consent for injections by the participant

A decision to discontinue further injections should be taken in consultation with the local PI and TSG.

An individual participant may stop PrEP early or be stopped early for any of the following reasons:

- HIV infection, confirmed by the local laboratory (see **section 6.7.1**)
- Unacceptable toxicity that precludes further PrEP including an estimated glomerular filtration rate that is confirmed to be below 50ml/min using the Cockroft-Gault formula
- Intercurrent illness that prevents further PrEP
- Withdrawal of consent for PrEP by the participant

As the participant's participation in the trial is entirely voluntary, they may choose to discontinue the trial treatment at any time without penalty or loss of benefits to which they are otherwise entitled.

Although the participant is not required to give a reason for discontinuing their trial treatment, a reasonable effort should be made to establish this reason while fully respecting the participant's rights.

Participants who have received at least one injection or one PrEP tablet will be asked to remain in follow-up. The participant may withdraw their consent to further visits at any stage of the trial. If a participant is withdrawn from follow-up, refer to **Section 6.10**.

Data that are already collected from participants who stop follow-up early will be included in the analysis.

A decision to discontinue injections in participants may follow a recommendation by the IDMC and or TSC following the interim analysis (see **Section 9.4**) or following a temporary interruption (see **Section 5.2.7**) if the issue with a vaccine product cannot be resolved. Enrolled particpants allocated to a discontinued regimen will not receive any further injections and will be informed verbally, and once approved in writing at the next visit. They will continue in follow-up according to the recommendation of the IDMC and TSC.

5.8 ACCOUNTABILITY & UNUSED VACCINES AND DRUG

The Pharmacist will ensure that all products are dispensed in accordance with the protocol, the study specific SOM, and Local Pharmacy Working Instructions, and that records are maintained of receipt, dispensing and destruction of all supplies.

At the end of the trial, accountability will be checked by the designated member of staff responsible for the inventory and by the trial monitors. The Sponsor and the PI will retain copies of the complete accountability records and copies will be provided to the providers of the vaccines for the relevant product, and Gilead Sciences plc for the PrEP agents.

All used vials of injections and matched placebos will be kept at the respective site pharmacy for the duration required by the in-country regulatory authorities and will be destroyed in accordance with institutional rules and national regulations. Destruction of MVA vials will comply with the regulations on GMO products where these are considered GMO. The associated packaging will be retained at the pharmacy in compliance with the applicable national regulations and at least until monitoring is completed and monitors give instruction for their destruction.

For unused vaccine and PrEP products, the Sponsor will provide instructions for their return/destruction in agreement with the supplier of each product.

Following IMP destruction, the pharmacist at each site must complete a certificate of IMP destruction and send it to the MRC/UVRI and LSHTM with copies to the Sponsor, who will subsequently provide a copy to the respective product providers.

5.9 COMPLIANCE & ADHERENCE

5.9.1 INJECTIONS

All injections will be administered by site staff and recorded in the CRF according to the SOM. If an injection is not given within the ideal window (see **section 5.2.7**), this will be recorded in the CRF together with the reason. The data will be entered in the study database and compliance with the vaccine schedule will be reported to the TMG monthly by the MRC/UVRI and LSHTM coordinating team.

5.9.2 PREP

Information about how to take PrEP will be provided to all participants.

Participants will be asked if they are taking PrEP, and if so how many days ago they last last took PrEP, at screening and at visits 2, 4, 6-10, 12, 14, 15 and every 24 weeks thereafter. At visits 4, 6-10, 12, 14, 15 and every 24 weeks thereafter, participants will be asked if they have had sex without a condom and if so, the number of tablets they have taken in the two days before and after this sex act. This will be recorded in the CRF and study database.

Participants will be asked to provide a urine specimen at visits 4, 6, 7, 12, 15 and every 24 weeks thereafter according to Table 1. This will be tested in the clinic for the presence of tenofovir. Staff will explore the possible reasons for any discrepancies between the participant self-report and the result of the urine test with participants.

Blood will be collected at visits 2, 6, 9, 12 and 15 and every 24 weeks thereafter in order to prepare a dry blood spot (DBS) for later analysis of intracellular drug levels which can be categorised according to the number of tablets consumed per week as follows:

- 4 or more tablets a week
- 3 or less tablets a week
- no tablets

This information will be available for the analysis (see **section 9**) but not in clinic.

5.10 TREATMENT DATA COLLECTION

Study staff will collect information on the CRF and enter this in the study database, and Pharmacy staff will maintain the Injection and PrEP Accountability Logs which will be stored securely in the pharmacy according to the Local Pharmacy Working Instructions.

5.11 NON-TRIAL TREATMENT

5.11.1 Medications Not Permitted

The following treatments cannot be given concomitantly but would take priority over trial treatment if required to manage an emergent cancer or other serious condition:

- Chemotherapeutic drugs
- Immunomodulators and systemic immunosuppressive treatments
  - Glucocorticoids
  - Cyclosporine
  - Methotrexate
  - Interleukins and/or cytokines
  - Immunotherapies (including TNF blockers)

If it is necessary for a participant to receive treatment with any of these products, the PI or Medical Delegate should consult the TSG to discuss the planned treatment and plans for stopping the participant's vaccination schedule.

5.11.2 Medications To Be Used With Caution In Conjunction With PrEP

Medications that can impair renal function should be used with caution in conjunction with either PrEP agent, particularly if the estimated glomerular filtration rate is <90 ml/min.

Co-administration of PrEP with drugs that are eliminated by active tubular secretion may increase concentrations of emtricitabine, tenofovir, or the co-administered drug.

5.11.3 Other Vaccinations

Vaccination with licensed vaccines should be avoided during the study because they may impact on the immune response. If needed for clinical reasons, PIs or Medical Delegate should consult the TSG

for advice with regard to timing, which will be up to 28 days later. Every effort will be made to vaccinate individuals after the key time points for collecting specimens for the immunological assays.

5.11.4 Treatment After HIV Seroconversion

Participants diagnosed HIV positive will be referred to local clinical centres for HIV treatment. Participants who are on PrEP when they have a confirmed HIV infection need a third drug to control viral replication. Treatment will be offered according to local and national guidelines, and organised through referral pathways according to local practice. If participants are taking PrEP at the time of diagnosis, the guidelines may allow them to continue the drug and add in a third agent.

5.12 CO-ENROLMENT GUIDELINES

Co-enrolment in previous or future trials is considered in **Section 3.4**.

6 ASSESSMENTS & FOLLOW-UP

Potentially eligible participants will be identified largely from the Registration Cohort and informed about the trial at a routine visit or through contact with the study staff which might happen at a recruitment event. If interested, they will be provided with written information or provided with information using visual aids which may include a video. Regardless of whether or not they can read, they will be given the written information to take away and advised to go through this with a friend or family member. They will be encouraged to discuss participation with their partners, friends and family, and invited to make an appointment for a screening visit. Individuals who attend a recruitment event who are not enrolled in the Registration Cohort will be screened and may be enrolled in the cohort first.

The Delegation Log will determine which members of the study team are authorised to conduct the assessments and procedures described in this section. In order to ensure the study staff responsible for assessing adverse events remain blind to the vaccine allocation, pharmacists who are independent of the study team will be delegated with the responsibility of drawing up the vaccine or placebo.

Additional details can be found in the SOM, SMP and Laboratory Analytic Plan (LAP).

6.1 TRIAL ASSESSMENT SCHEDULE

The trial assessment schedule consists of a screening visit (visit 1), an enrolment visit which must take place within 8 weeks of the screening visit and which defines week 0 (visit 2).

The first injection will take place at week 0 (visit 2) and further injection visits are at visits 4, 8 and 12. The latest timepoint that the second, third and fourth injection visit can take place is indicated in Table 1 and is the end of the 13th, 40th and 68th week from enrolment respectively. Visits 3, 5, 6, 9, 10 and 13 have specific activities and procedures that relate to the collection of key safety and efficacy variables following the injection visits and the ideal timing relates to each injection. The earliest timepoint and upper target for visits is also given in Table 1. Provided visits occur in the period between the earliest timepoint and upper target there is no need to complete a protocol deviation form. If the participant has passed the upper target for the visit, a protocol deviation form should be completed.

Safety follow-up visits (or calls) to ask about all adverse events are at visits 3,5-7,9-11,13-15. After visit 15, follow-up visits will continue every 12 weeks for HIV testing through to the end of the trial, and participants will be asked about any serious illness or admission to hospital at these visits. These visits will continue to the last 3 months of the trial when everyone will have a final study visit. Additional visits may be undertaken if indicated, for example to investigate or follow-up an adverse event or abnormal laboratory result.

The clinic assessments, samples and volumes to be collected are outlined in **Table 1 Trial Assessment Schedule for Investigators**. Most of the visits during the trial require 10mls to be drawn for HIV tests and store. Larger volumes are required at visits when blood is collected for serology and PBMCs at visits 2 (133mls), 6 (43mls), 9 (138mls), 13 (129mls) and 15 (119mls). The maximum blood volume drawn for a participant who completes the schedule through to visit 15 is 679mL and will not exceed 200mL in any 3 month period, the equivalent of half a unit of blood. This does not include any additional blood volume that would be required if a safety or HIV test needs to be repeated. After visit 15, 10mL of blood will be collected every 12 weeks for HIV testing, and 5ml every 24 weeks for syphilis serology (if indicated). Amounts are specified in **Table 1**. The required volumes per visit together with sample collection and processing guidelines are described in detail in the LAP
.

The visit windows are defined in the in **Table 1**.

6.2 PROCEDURES DURING THE SCREENING PERIOD

6.2.1 INFORMED CONSENT

At the screening visit, participants will have the opportunity to ask questions and have a full discussion of the information provided in writing and/or visually and verbally. This will include the following key messages:

- That there is a 1 in 3 chance that they are assigned to a placebo injection, but this may change during the trial to 1 in 2 if one of the trial arms is discontinued or interrupted
- That we do not know if the active vaccine combination regimens will prevent HIV
- That condoms and PrEP are known to reduce the risk of acquiring HIV when used or taken consistently
- That pregnancy is to be avoided until 18 weeks after last vaccine as the safety of vaccines is not known
- That the immune responses made by their bodies to the vaccines could show up in future HIV tests

Participants will be shown images of the PrEP pills. They will be asked if they discussed their participation with anyone and if any problems arose because of this. If they wish to proceed, they will be given an informed consent form to read and sign prior to any investigations. It will be dated and countersigned by the investigator or delegated person, who explained the study to them. If they are unable to read and write, they will be asked to place their thumbprint on the informed consent in the presence of an independent witness who has been present for the discussion. The witness cannot be a member of the site study staff.

A copy of the consent form will be provided to the participant and one copy kept in the study file according to local procedures. If they have not already done so, they will be encouraged to discuss their participation with their partners, friends and family.

6.2.2 ELIGIBILITY

After informed consent has been collected, a unique trial identifier will be assigned by the study staff.

To assess eligibility demographic information, a past and current medical history, details of current and recent medication including use of PrEP and herbal remedies will be collected during a structured interview. The interview will be conducted by a member of the study team and recorded on the appropriate CRF, which will be identified by the unique study number. The number will be allocated to the participant after consent has been collected. Details of contraception and sexual history will be collected to assess the risk of pregnancy arising in the participant or their partner, and the risk of HIV acquisition respectively . Participants who are using condoms or taking PrEP consistently are not considered at risk of acquiring HIV. Therefore they would not comply with eligibility criteria 5. Details of smoking, use of alcohol and recreational drugs will also be collected as these can impact on the risk of HIV, the strength of immune responses and lead to abnormal routine laboratory parameters.

The screening examination will include weight (kg), height (cm), , blood pressure, pulse, respiratory rate, inspection of the skin at the injection site, respiratory, cardio-vascular, abdominal, and neurological examination. An assessment of cervical, axillary and inguinal lymph nodes will also be undertaken.

6.2.3 INVESTIGATIONS

Blood (as per **Table 1**) will be collected for point of care HIV testing. A sample will also be sent to the local laboratory for testing in accordance with the HIV testing algorithm in the LAP. Residual from the sample will be stored in case it is necessary to conduct additional HIV tests to assist with the statistical analyses.

Blood will be collected for analysis of the following routine laboratory parameters in the local laboratories and recorded in the appropriate CRF:

- Haemoglobin
- Alanine aminotransferase, aspartate aminotransferase, alkaline phosphatase, bilirubin
- Glucose
- Creatinine and estimated glomerular filtration rate using the Cockroft-Gault formula
- hepatitis B surface antigen (screening only)
- RPR (and TPHA if required)

A first pass urine specimen will be collected from men and from women, and sent to the local laboratory to screen for gonorrhoea and chlamydia. If indicated by the sexual history participants may be asked to provide a rectal swab. Staff will explain how to collect the swab and participants will be able to do so in private.

Urine will be collected for a point of care test to detect tenofovir. Any discrepancies between the result of the test for tenofovir and self-report will be explored with the participant and recorded on the CRF.

For female participants of childbearing potential a pregnancy test will be performed by analysis of a urine sample for Human Chorionic Gonadotrophin (HCG).

6.3 PROCEDURES AT ENROLMENT

This visit should take place within 8 weeks following the screening visit. If more than 8 weeks have elapsed study staff should consult the SOM to find out which screening assessments need to be repeated.

6.3.1 ELIGIBILITY

Study staff will check that participants understand the key messages contained in the information sheet and listed in **section 6.2.1.**

Study staff will review the routine laboratory parameters and screen for STIs collected at screening and determine whether there are any clinically relevant abnormalities that require further investigation prior to randomisation. STIs will be treated appropriately, but the participant can proceed to randomisation on the day, if otherwise eligible.

Participants will review any new adverse events or new medications since the screening visit and for female participants, any changes in contraception. All this information will be recorded on the appropriate CRF according to the SOM. The Risk assessment CRF will also be administered.

Study staff will review the risk of the participant acquiring HIV again to see if anything has changed since screening, and conduct a point of care HIV test in accordance with the LAP.

For female participants of childbearing potential a pregnancy test will be performed by analysis of a urine sample for Human Chorionic Gonadotrophin (HCG) and a negative result will be confirmed before each injection.

The review of Eligibility will be completed using the appropriate CRF.

Prior to randomisation, blood will be collected for 4th generation ELISAs, and to store for future immunological assays, HIV RNA, and intracellular tenofovir diphosphate levels.

6.3.2 Randomisation

Eligible participants will be randomised in a concurrent 3 x 2 factorial design to

- one of three injection regimens in a 1:1:1 ratio (the placebo group will be randomised 1:1 to match the volume of the protein injection in the combination arms)
- TDF/FTC or TAF/FTC

Study staff will be able to generate the randomisation code electronically and add the unique randomisation identifier to the prescription together with the unique trial identifier to take to pharmacy to prepare the appropriate products for injection and dispense PrEP. In the event that the database is not available for example due to internet problems, study staff will write the prescription using the trial identifier only and take this to pharmacy. The pharmacist will be able to access the code manually or via telephone, and add this to the prescription, providing study staff with the appropriate PrEP agent to give to the participant and two products for injection, one in each arm.

6.3.3 Injection

Prior to injection, study staff will conduct an assessment relevant to the solicited adverse events described in section 6.5, and record the details on the appropriate CRF. This will include vital signs, a measurement of temperature, and inspection of the site where injections will be administered in each arm.

The injection will be labelled so that study staff can identify which arm the product is to be injected into. They will not know whether the product is active or placebo.

Study staff should administer the injection into the deltoid muscle of the designated upper arm and record this in the appropriate CRF including the time of injection.

The DNA/placebo vaccine is 1ml in volume and should be injected into the deltoid muscle of the left arm. The MVA/placebo is 1ml in volume and is slightly opaque and should be injected into the deltoid muscle of the left arm. The two protein/placebo vaccines are of 1ml for AIDSVAX® B/E/placebo and 0.45ml for CN54gp140 in MPLA-L/placebo and should be injected into the deltoid muscle of the right arm. All syringes will be covered throughout the trial with a label to obscure the contents but the volume will be revealed to clinic staff because of the position of the plunger. It will therefore be possible for staff to differentiate between 1 and 0.45ml but they will not be able to differentiate between active and placebo injection.

In the event that study staff administer the product into the opposite muscle to the one intended a deviation should be noted (see SOM for details).

Following injection participants should remain in clinic for up to 60 minutes, in order to assess solicited adverse events at 25-60 minutes following injection. Study staff will go through the **Vaccine diary cards** with participants and explain how and when to complete these over the following 7 days.

6.3.4 PrEP

Participants should be given 60 tablets at weeks 0 (visit 2) in order to have sufficient supply to last 30 days beyond their next scheduled dispensing visit at week 4

Study staff will review the **PrEP diary cards** with participants. They will ensure that participants understand when they are at risk of exposure to HIV and know how long it takes for PrEP to become effective.

They will review any concerns the participant has about side-effects from PrEP and medicines in general. They will advise the participant how to remember to take a tablet every day by linking this to a daily routine.

Participants will be asked whether they have informed partners, friends and family since the screening visit, and whether any issues arose. If they have not informed the other people they live

with, they will be asked how they plan to store the drug and whether they have concerns about others finding it.

6.4 TREATMENT PROCEDURES DURING FOLLOW-UP

Injections will be administered at visits 4, 8 and 12 from randomisation according to the schedule in **Table 1**. PrEP will be dispensed at visits 4 and 7. Additional PrEP may be required at other weeks or at an unscheduled visit to ensure drug continues for two weeks after the third injection.

6.4.1 INJECTIONS

Study staff should follow the procedures outlined in **section 6.3.3** with respect to assessments prior to injections, administration of the injections, and assessments after the injections in clinic. The information should be recorded on the appropriate CRF.

Study staff should endeavour to administer the injections in the period between the earliest timepoint and upper target, as defined in **table 1**. Provided the visit is within the latest timepoint window outlined the injections may proceed without further consultation. Study staff should complete a protocol deviation form if the injection occurs after the upper target window..

.

6.4.2 PREP

Study staff should follow the procedures outlined in **section 6.3.4** with respect to an assessment of understanding of risk and discussion of concerns.

Participants should be given 60 and 90 tablets at visits 2 and 4 in order to have sufficient supply to last 30 days beyond their next scheduled dispensing visit at visits 4 and 7. Additional drug may need to be dispensed at other visits including an unscheduled visit to ensure the participant has enough to last for 2 weeks after the third injection. See **section 5.5.5**.

For safety and adherence assessments, see **sections 6.5.4 and 6.6** respectively.

6.5 PROCEDURES FOR ASSESSING SAFETY OF TRIAL TREATMENTS

6.5.1 SOLICITED ADVERSE EVENT ASSESSMENT

Vaccines are associated with a number of well-characterised local, systemic and laboratory reactions referred to as solicited adverse events (**Table 7**). These adverse events will be purposively collected during the structured interview, examination and laboratory testing.

Local and systemic assessments will take place on the day of each injection at the following time points: before the injection, and 25- 60 minutes after the injection. Participants will receive two injections at every time point. The local adverse events will be specific to each arm, but the systemic ones will only be recorded once and will be timed against the second injection.

Participants will be provided with **Vaccine diary cards** to assist collection and grading of adverse events that start within 7 days of the injection.

Follow-up of solicited adverse events may occur at a clinic visit or through a phone-call one week after the injections at visits 3 and 5 respectively and at a visit 2 weeks after 3rd and 4th vaccines (visits 9 and 13 respectively). Study staff will go through the list of solicited adverse events in a structured interview and record these together with grade on the appropriate CRF. If any of the events reported on the phone are still moderately severe (grade 2) or worse, participants will be invited to the clinic for review.

Blood (14mls) for routine safety parameters will be collected 4 weeks after the 1st and 2nd injections at visits 4 and 6, and 2 weeks after the 3rd and 4th injections at visits 9 and 13 and at any other study

visit if required to confirm or follow-up an abnormal result for one of the listed parameters in **Table 7.** If the total bilirubin is elevated, study staff will request a result for conjugated bilirubin in order to grade the abnormality and determine any action to be taken with respect to further investigation and interruption to the vaccine schedule.

Table 7: Solicited Adverse Events

TYPE	EVENT	
Local AEs within 7 days<br>(injection site)	Pain or tenderness<br>Itching<br>Redness<br>Swelling (soft)<br>Induration (hard)	
Systemic Clinical AEs within 7 days of injections	Temperature<br>Chills<br>Myalgia/flu-like general muscle aches<br>Arthralgia<br>Malaise (excess fatigue)<br>Headache<br>Nausea<br>Vomiting<br>Generalised rash<br>Generalised itching	
Systemic Laboratory AEs<br>4wks after 1st & 2nd injections<br>2wks after 3rd & 4th injections	Creatinine<br>ALT/AST<br>Alkaline phosphatase<br>Total bilirubin<br>Glucose	Haemoglobin

6.5.2 OTHER ADVERSE EVENT ASSESSMENT

Information on other adverse events will be collected through an open question about health at study visits 2-15. Study staff will record the diagnosis or the signs/symptoms if a diagnosis is not apparent, the date of onset and the date of resolution if appropriate. If the event is ongoing, it may be appropriate to conduct a symptoms directed examination. After visit 15, staff will only collect and record information on serious adverse events at every 12 weekly visit.

Events should be graded according to the Division of AIDS (DAIDS) Table for Grading the Severity of Adult and Paediatric Adverse Events version 2.1, July 2017. (https://rsc.niaid.nih.gov/sites/default/files/daidsgradingcorrectedv21.pdf).

The relationship to injection or PrEP will be determined by the investigator according to the definitions provided in **Section 7**.

All of this information will be recorded on the appropriate CRF.

In the event that the participant is unable to attend a safety follow-up visit for personal reasons, the interview may be conducted on the telephone or at a home visit if the participant is agreeable.

Study staff will make every effort to follow adverse events to resolution or stabilisation in order to report the information on the appropriate CRF during the trial.

After the trial has closed and the database has been locked, additional information regarding serious adverse events that comes to the attention of the study staff should be reported by email to the CI and MRC/UVRI and LSHTM using the TSG email.

6.5.3 PrEP Adverse event assessment

TDF/FTC and TAF/FTC have similar adverse event profiles in terms of associated adverse events although the frequency and severity of some events are lower with TAF/FTC because the tenofovir levels in the plasma are lower.

Study staff will use the reference safety information provided in section 4.8 of the SPC for each drug to assist in determining the relationship of adverse events to injection, PrEP or other causes according to the definitions provided in **Section 7.3.1.C** and **Table 9.**

Elevations in serum creatinine have been associated with tenofovir in HIV negative individuals taking the drug as PrEP. Serum creatinine will be measured 4 weeks after the first and second injection and 2 weeks after the third. Additional serum creatinine measurements is needed at other visits through to visit 9 if estimated creatinine clearance falls to <60 ml/min to monitor renal function more closely. If serum creatinine suggests that the estimated glomerular filtration rate has fallen below <50ml/min using the Cockroft-Gault equation, this will be repeated in order to inform any action to be taken with respect to discontinuation of PrEP (see **Section 5.5.7)** . This information will be recorded on the appropriate CRF.

6.5.4 Pregnancy assessment

Female participants of child-bearing age who are not sterilised will be asked in detail about contraception at screening and enrolment (see **sections 6.2** and **6.3**), at each injection visit and at visit 14, 14 weeks after the last injection) and whether there have been any changes at the other study visits. Answers will be recorded on the appropriate CRF. A pregnancy test will be performed at most visits to visit 15 other than visits 9 and 13 by analysis of a urine sample for Human Chorionic Gonadotrophin (HCG) and recorded on the CRF. A pregnancy test may be conducted at visits 9 and 13 if there have been any changes and the participant or study staff have reason to believe the participant could be pregnant. Injections cannot be administered if the pregnancy test is equivocal and must be discontinued if pregnancy is confirmed and continued(see **section 5.7**). If the pregnancy arises during the period of follow-up when PrEP is being provided, study staff will discuss the risk and benefits of continuing PrEP during pregnancy (see **section 5.5.6**). Participants who are on TAF/FTC will need to switch to TDF/FTC if they continue PrEP.

Male participants will be asked whether any of their partners has become pregnant at each visit and answer recorded on the CRF.

Information on the outcome of pregnancies will be collected on the appropriate CRF directly from the participant or indirectly through their partner (see **section 7.2.1**). A consent form will be required from the female partner of a participant to collect details of the outcome/baby .

6.6 PROCEDURES FOR ASSESSING ADHERENCE TO PREP

Adherence to PrEP will be assessed at visits 4, 6, 7 and 9 based on self-report, dispensed pills and one or both of two laboratory based methods. Participants will be provided with a simple **PrEP diary card** to record pill-taking and sex acts to assist with self-report.

- Urine will be collected at visits 4, 6, 7, 12 and 15 and every 24 weeks thereafter in all participants and tested in the clinic for the presence of tenofovir
- Blood will be collected to prepare dry blood spots for later analysis at visits 6, 9, 12, and 15 and every 24 weeks thereafter()

Self-report and the result of the urine test in clinic will be collected on the appropriate CRF, and any inconsistencies probed for a reason. Dispensed pills will be collected on the PrEP Accountability Log

as well as the CRF and used to calculate the medicine possession ratio (number of pills dispensed as a proportion of the number of days from enrolment). The results of the dry blood spots will not be provided directly to the participants or study staff, but to the statistical team for the analysis.

A urine test for tenofovir may be conducted at other weeks if indicated according to the SOM.

6.7 PROCEDURES FOR ASSESSING EFFICACY

For the vaccine analysis, the efficacy endpoint is HIV infection acquired after visit 9 by a participant who has received 3 vaccinations and is HIV negative at that visit. For the PrEP analysis, the efficacy endpoint is HIV infection acquired up to and including visit 9 by a participant who was HIV negative at week 0 (visit 2).

There are two aspects to consider: firstly the need to distinguish between HIV infection and a vaccine induced response, and secondly the need to determine the timing for the analysis.

6.7.1 CONFIRMATION OF HIV INFECTION

HIV-uninfected individuals who participate in prophylactic HIV vaccine studies may develop HIV-specific antibodies because of an immune response to the candidate HIV vaccine, referred to as vaccine induced seropositivity (VISP). These antibodies may be detected in common HIV assays, causing the test to appear positive even in the absence of actual HIV infection.

Point of care tests will not be used during follow-up in order to ensure that staff remain blind to the allocation as responses to the recombinant protein vaccine may elicit reactions in the point of care tests.

Study staff will collect a sample in the appropriate tube for HIV ELISA assays and plasma for RNA viral load in case this is required. They will send the samples to the local laboratory at visits 2, 4, 6-15. Follow-up after visit 15 will continue for all participants with collection of a sample for HIV ELISA and RNA viral load every 12 weeks up to and including the final visit which will be take place in the last 3 months of the study (2023-2024). The samples will be processed according to the LAP and HIV testing algorithm and any residual stored for future tests including assessment of resistance mutations. Samples collected at visits 2, 8 and 12 will not be processed in real-time as the next timepoint is within 2-4 weeks.

After determining HIV status in the local laboratory, the result will be returned to the clinic as:

- No evidence of HIV infection
- HIV infected

Samples will continue to be collected until it is possible to determine negative or positive status. Trial treatments can continue during this time.

Participants who are HIV positive will be counselled and given a letter of referral to their preferred provider of care and assisted with transport if necessary.

At the visit at which HIV status is confirmed as positive and the referral organised, 61ml of additional blood will be collected for humoral and cellular responses (see **sections 6.8.1** and **6.8.2**), virological analysis including resistance testing and drug levels if appropriate to the period of follow-up.

The 61ml of additional blood will be collected and stored for:

- 5ml CD4 T-cell count,
- 5ml plasma HIV RNA
- 4ml PrEP drug level testing
- 37ml cellular and humoral immune responses
- 10ml viral isolation and sequencing.

Although additional blood will be required at this visit, the overall volume collected will be reduced as subsequent collections will be modified, unless the seroconversion occurs after week 74.

Follow-up will continue for at least 18 weeks after the last injection in order to determine whether any pregnancies occur in the participant or their partner, for safety, and to ensure linkage to care. The visit schedule will be modified to reduce the burden on the participant for seroconversions that occur before week 74.

6.7.2 Timing of HIV infection

An Endpoint Committee will review the results from the local laboratory and may request additional testing of stored blood in order to determine the timing of the first HIV positive result which will be categorised and managed as follows:

- HIV positive at enrolment visit — excluded from all analyses
- HIV positive at visits 4-9 — PrEP analysis
- HIV positive at visits 10 on — vaccine analysis

Although participants who test positive for HIV will have been infected at a date prior to the test, for the purpose of the analysis the timing of infection is taken to be the time of the first HIV positive sample.

See **section 6.14** for post-trial testing of participants who develop vaccine induced seropositivity (VISP).

6.8 PROCEDURES FOR ASSESSING IMMUNE RESPONSES

Samples will be collected at visits 2, 6, 9, 13 and 15, and if they contract HIV at the visit a participant is informed that they have HIV infection. Further details on the timing of these samples and the window within which they are to be collected can be found in the SOM and LAP. Specimens will be collected, transferred to the local research laboratory, aliquoted and stored according to LAP. The aliquots will be used for the secondary and exploratory humoral responses.

Samples may be used for other testing and research related to furthering the understanding of HIV and the immune system. In addition, cryopreserved samples may be used to perform additional assays to support standardization and validation of existing or newly developed methods.

6.8.1 Secondary Immune responses

The secondary immunological outcome is the quantitative binding antibody responses against CN54gp140 and AIDSVAX gp120 measured by ELISA.

Quantitative binding antibody ELISA assays will be performed in a single laboratory for the purpose of the trial analyses to minimise the risk of variability, but these assays can also be conducted locally as part of the capacity building where funding permits.

6.8.2 Exploratory Immune responses

The following exploratory immunological assays may be performed based on the initial results of the trial and the amount of funding available. These exploratory assays will provide an in-depth profiling of the quality of the immune responses, and enable the evaluation of immunological correlates of risks associated with HIV acquisition and ultimately identification of immune correlates predictive of vaccine efficacy.

- **Neutralizing antibody (nAb) responses**: HIV-1–specific nAb assays may be performed on serum samples from study participants. Tier 1 assays will test neutralization of HIV-1 strains represented in the highly neutralization sensitive tier 1 viruses. The tier 2 assays will test neutralization of a panel of heterologous primary isolates.
- **Binding antibody responses** measured by binding antibody multiplex assay (BAMA): Total binding IgG antibodies to HIV-1 Env proteins (including V1/V2 regions of interest) will be

measured on serum samples from study participants. Specimens from other HIV antigens and Ab isotypes may also be assayed based on the results of the initial assay.

- **ADCC and other functional antibody responses**: antibody-dependent cell-mediated cytotoxic activity and other antibody functional assays may be performed
- **Frequency of CD4 and CD8 T cell producing cytokines** will be assessed as an exploratory immunological outcome by multiparametric flow cytometry after in vitro stimulation of thawed PBMC with HIV-specific antigens using intracellular cytokine staining (ICS) including, but not limited to IFNψ, IL-2, and TNFα. Additional assays may be performed to further investigate the immune responses elicited by the study vaccine regimens. Examples include, but are not limited to additional binding antibody assays, and supplemental cellular assays (T cells and B cells).

A single laboratory will conduct each of the assays for the purpose of the trial analyses, but assays can also be conducted locally as part of the capacity building where funding permits.

6.8.3 GENOTYPING

Molecular human leukocyte antigen (HLA) typing may be performed on enrolled participants using cryopreserved PBMC collected at baseline, initially on specimens from participants who demonstrate vaccine-induced immune responses following vaccination. Other participants (including placebo recipients) may be HLA-typed to support future immunological studies. The genetic testing will be limited to genes related to HIV and the immune system.

6.9 PROCEDURES FOR ASSESSING CONTEXTUAL DATA

6.9.1 SELF-REPORTED SEXUAL AND ADHERENCE BEHAVIOURS

Self-reported sexual and adherence behaviours will be collected in the CRF and supported by the PrEP diary card.

The **PrEP diary card** will be a simple record for noting pill taking and acts of sexual intercourse when a condom was used or not, in order to determine pill use in the 2 days before and following sex acts without a condom. The facility to enter this information directly into an application on a smartphone may be available for participants that have secure access to a mobile phone with capacity to host the application. Participants will be invited to share their record with staff during a study visit to assist in accurate completion of the CRF. Self-report will be checked against the result of the point of care urine test. Study staff will be prompted to ask an open question about discrepancies between reported pill use and objective measures of drug,. The answers will be recorded on the CRF and may be entered into software designed to analyse free text.

Participants will be asked about pill taking in the 2 days prior to the visit, and the 2 days before and after the last sex act without a condom when they attend the study visits to collect drug,

Questions to capture risk behaviours will be completed at screening, visits 2, 4, 6, 7, 8-10 , 12, 14, and 15 and every 24 weeks thereafter until the end of the study.

In depth interviews (one to one discussions) will be conducted in up to 10% of participants purposively selected to represent the patterns of risk and adherence behaviours emerging from the quantitative data and these interviews will provide insights into the motivations and barriers that facilitate and obstruct behavior change respectively. The one to one discussions will be explained to the participants using the Supplementary Participant Information Sheet and written informed consent will be collected prior to participation by an individual trained in interview techniques. The interviewer will be provided with a summary of the information collected on the CRF. They will interview participants about sexual and adherence behaviours, study and PrEP acceptability using the one to one discussion guide.

Group discussions may be conducted with a subset of participants (with approximately 8 participants per group) by staff trained in the technique who are independent of the clinic team. Some participants will be purposefully selected for participation in group discussions and others will be invited on an ad hoc basis. Selection will be directed after the social science group review of the summary quantitative data. The group discussion will be explained to participants using the Supplementary Participant Information Sheet and written consent collected on the Consent Form .

Structured debriefs of community and counselling staff will be conducted by a social scientist during the course of the study. Typically, these will note conversations observed or initiated by staff with and between participants, non-participants, clinic staff and community workers. The notes will be anonymous and recorded using a template.

6.9.2 Contextual Data In Seroconvertors

Participants who seroconvert at any time during follow-up will also be invited to take part in an in-depth interview as described above. Information about sex acts without condoms or PrEP since the previous negative result, the number and characteristics of new partners will be collected.

6.10 EARLY STOPPING OF FOLLOW-UP

If a participant chooses to discontinue their trial treatment, they should continue to be followed up. If they do not wish to attend any further visits then their decision must be respected. The MRC/UVRI and LSHTM should be informed of this in writing according to the SOM.

If the participant is willing to complete interviews and provide samples at the time of their last visit then study staff should follow the schedule for visit 9 for participants who leave the study prior to this visit , or visit 15 for those leaving later.

The data already collected in the trial will have been extracted from the study database for monitoring and analysis purposes, and cannot easily be redacted. The data will not contain name and date of birth. The consent provided at screening will be assumed to apply for future use of these data in analyses for public health benefit.

Where possible study staff will check that the participant still consents to the future use of stored samples already collected, and if this is refused the samples will be located using the unique trial identifier and destroyed. If it is not possible to check consent, then the consent provided at screening will be assumed to apply. The samples will not contain any personal identifiers such as name and date of birth.

Participants may change their mind about stopping trial follow-up at any time and re-consent to participation in the trial, if this is practical in relation to the time they enrolled and the time since they decided to stop follow-up.

The IDMC and TSC may make a recommendation to amend follow-up following the interruption or discontinuation of product.

6.11 PARTICIPANT TRANSFERS

It is extremely unlikely that a participant will move from the area of one trial centre to another, but not impossible, especially in Tanzania where there are two sites. In the event that a participant does transfer, a copy of the participant’s CRF should be provided to the new site and the participant will need to sign a new consent form. Once this has been done, the new site will take over responsibility for the participant; until this has been done, responsibility for the participant lies with the original site.

6.12 LOSS TO FOLLOW-UP

Study staff will make every effort to contact participants who do not attend their scheduled visits according to the SOM and local procedures. At least three attempts will be made to contact the participant during the period of protocol-planned deviation described in section 5.2.7 for injection visits and 5.5.5 for PrEP dispensing visits.

Participants will not be considered lost to follow-up until the trial has ended, unless they have left the country with no plans to return, and with no means to ascertain HIV status. Additional efforts will be made at the time of key timepoints (weeks 26 and 74) and in the final months of the trial, at the very least to ascertain HIV status.

6.13 COMPLETION OF PROTOCOL FOLLOW UP

The trial will be closed when all participants have made their final follow-up visit and assessments are completed including those to determine resolution of any adverse events, the data entered into the database and checked and the safety database locked.

6.14 POST-TRIAL HIV TESTING

The institutional partners will continue to provide a clinical service for HIV testing through to the point of unblinding participants. After unblinding, a clinical service will continue to be provided to individuals who have a vaccine induced seropositive response in the local assays used for routine HIV testing. Arrangements will be made for participants who relocate.

7 SAFETY REPORTING

The principles of GCP require that both investigators and Sponsors follow specific procedures when notifying and reporting adverse events or reactions in clinical trials. These procedures are described in this section of the protocol. Section 7.1 lists definitions, Section 7.3 gives details of the investigator responsibilities and Section 7.4 provides information on MRC/UVRI and LSHTM responsibilities.

7.1 DEFINITIONS

The definitions of the EU Directive 2001/20/EC Article 2 based on the principles of GCP apply to this trial protocol. These definitions are given in Table 8.

Table 8: Definitions

TERM	DEFINITION
Adverse Event (AE)	Any untoward medical occurrence in a participant or clinical trial subject to whom a medicinal product has been administered including occurrences that are not necessarily caused by or related to that product.
Adverse Reaction (AR)	Any untoward and unintended response to an investigational medicinal product related to any dose administered.
Unexpected Adverse Reaction (UAR)	An adverse reaction, the nature or severity of which is not consistent with the reference safety information about the medicinal product set out in the Summary of Product Characteristics (SPC) or Investigator Brochure (IB) for that product.
Serious Adverse Event (SAE) or Serious Adverse Reaction (SAR) or Suspected Unexpected Serious Adverse Reaction (SUSAR)	Respectively any adverse event, adverse reaction or unexpected adverse reaction that:<br>▪ Results in death<br>▪ Is life-threatening*<br>▪ Requires hospitalisation or prolongation of existing hospitalisation**<br>▪ Results in persistent or significant disability or incapacity<br>▪ Consists of a congenital anomaly or birth defect<br>▪ Is another important medical condition***

*The term life-threatening in the definition of a serious event refers to an event in which the participant is at risk of death at the time of the event; it does not refer to an event that hypothetically might cause death if it were more severe, for example, a silent myocardial infarction.

Hospitalisation is defined as an admission, regardless of length of stay, even if the hospitalisation is a precautionary measure for continued observation. Hospitalisations for a pre-existing condition, that has not worsened or for an elective procedure do not constitute an SAE (see **section 7.1.3)

*** Medical judgement should be exercised in deciding whether an AE or AR is serious in other situations. The following should also be considered serious: important AEs or ARs that are not immediately life-threatening or do not result in death or hospitalisation but may jeopardise the subject or may require intervention to prevent one of the other outcomes listed in the definition above; for example, a secondary malignancy, an allergic bronchospasm requiring intensive emergency treatment, seizures or blood dyscrasias that do not result in hospitalisation or development of drug dependency.

7.1.1 Medicinal Products

An investigational medicinal product is defined as the tested investigational medicinal product (IMP) and the comparators used in the study.

7.1.2 Adverse Events

Adverse Events include:

- An exacerbation of a pre-existing illness
- An increase in frequency or intensity of a pre-existing episodic event or condition
- A condition (even though it may have been present prior to the start of the trial) detected after trial drug administration
- Continuous persistent disease or a symptom present at baseline that worsens following administration of the study treatment

7.1.3 Events That Are Not Adverse

The following Events are not considered to be adverse:

- Medical or surgical procedures; the condition that leads to the procedure is the adverse event
- Pre-existing disease or a condition present before treatment that does not worsen
- Hospitalisations where no untoward or unintended response has occurred, eg, elective cosmetic surgery, for delivery of a baby, or to facilitate an investigation that could be conducted as an outpatient

7.2 OTHER NOTABLE EVENTS

Notable adverse events which impact on the treatment schedules and therefore require expedited (within 24 hours of the investigator becoming aware of the event) reporting whether or not they meet the serious criteria include:

- Grade 3 and above solicited adverse events which last more than 72 hours
- Grade 3 and above laboratory adverse events that are confirmed on repeat testing if possible
- Any adverse event leading to a clinical decision to interrupt or discontinue the injection or PrEP schedule
- Significant social harm as a result of participating in the study
- Pregnancy within 18 weeks of an injection or whilst taking TAF/FTC (see **section 7.2.1**)

7.2.1 Pregnancy

Pregnancy is not an adverse event in itself, unless it results in a condition that meets the seriousness criteria defining SAEs (e.g. septic abortion). Pregnancy is a notable event as there are no safety data for the vaccine candidates, and insufficient data for TAF/FTC. All pregnancies in female participants will be followed-up until their outcome is known. Every effort will be made to determine the outcome of pregnancies in female partners of male participants.

7.3 INVESTIGATOR RESPONSIBILITIES

All non-serious AEs and ARs, whether expected or not, should be recorded in the participant's source notes and reported in the appropriate CRF and entered in the database within the agreed timescale as detailed in the SOM. SAEs, regardless of relationship SARs and notable events should be notified to MRC/UVRI and LSHTM within 24 hours of the investigator becoming aware of the event.

7.3.1 INVESTIGATOR ASSESSMENT

7.3.1.A Seriousness

When an AE or AR occurs, the investigator responsible for the care of the participant must first assess whether or not the event is serious using the definition given in Table 8. If the event is serious, then an SAE Form must be completed and MRC/UVRI and LSHTM notified within 24 hours.

7.3.1.B Severity or Grading of Adverse Events

The severity of all AEs and/or ARs (serious and non-serious) in this trial should be graded using the toxicity gradings in 2017 DAIDS toxicity grading table v2.1 July 2017 .

7.3.1.C Causality

The investigator must assess the causality of all events or reactions in relation to the trial therapy using the definitions in Table 9. There are five categories: unrelated, unlikely, possible, probable, and definite. If the causality assessment is unrelated or unlikely to be related, the event is classified as an S/AE. If the causality is assessed as possible, probable or definitely related, then the event is classified as an S/AR.

Table 9: Assigning Type of S/AE Through Causality

RELATIONSHIP	DESCRIPTION	S/AE TYPE
Definite	There is clear evidence to suggest a causal relationship and other possible contributing factors can be ruled out.	S/AR
Probable	There is evidence to suggest a causal relationship and the influence of other factors is unlikely.	S/AR
Possible	There is some evidence to suggest a causal relationship (for example, because the event occurs within a reasonable time after administration of the trial medication). However, the influence of other factors may have contributed to the event (for example, the participant's clinical condition, other concomitant treatments).	S/AR
Unlikely	There is little evidence to suggest that there is a causal relationship (for example, the event did not occur within a reasonable time after administration of the trial medication). There is another reasonable explanation for the event (for example, the participant's clinical condition, other concomitant treatment).	Unrelated S/AE
Unrelated	There is no evidence of any causal relationship	Unrelated S/AE

7.3.1.D Expectedness

If there is at least a possible involvement of the trial treatment or comparator, the investigator should make an initial assessment of the expectedness of the event, although the Sponsor has the final responsibility for determination of expectedness. An unexpected adverse reaction is one that is not listed in the reference safety information providedin the approved IB or SPC or one that is more severe than previously reported. The definition of an unexpected adverse reaction (UAR) is given in Table 8.

7.3.1.E Notification

MRC/UVRI and LSHTM must be notified of all SAEs regardless of relationship, and notable events, within 24 hours of the investigator confirming the event meets the criteria.

Investigators must notify MRC/UVRI and LSHTM of all SAEs occurring from the time of randomisation until trial closure.

The investigator will notify local and national ethics committees and regulatory authorities as per local and national guidelines

7.3.2 Notification Procedure

The SAE and Notable Event Forms must be completed by an investigator named on the Signature List and Delegation of Responsibilities Log, who is responsible for the participant's care; this will be either the Principal Investigator or another clinically qualified person with delegated authority for SAE reporting and review of notable events. Due care should be paid to the grading, causality and expectedness of the event, as outlined above. In the absence of the responsible investigator, the form should be completed and signed by a member of the site trial team and emailed securely as appropriate. The responsible investigator should subsequently check the form, make changes as appropriate, sign and then re-send to MRC/UVRI and LSHTM as soon as possible.

The minimum criteria required for reporting are the participant's trial number name of investigator reporting, the event, and why it is considered serious or notable.

The Form must be securely emailed to MRC/UVRI and LSHTM to **PrEPVacc_trial_safety@mrcuganda.org**

Follow-up: the initial report must be followed up with details when these are available; participants must be followed up until clinical recovery is complete and laboratory results have returned to normal or baseline, or until the event has stabilised. A further Form, indicated as 'Follow-up' should be completed and securely emailed to MRC/UVRI and LSHTM as information becomes available. Extra, annotated information and/or copies of test results may be provided separately, with personal identifiers removed.

Staff should follow their institution's procedure for local notification requirements.

EXPEDITED REPORTING
Within 24 hours of becoming aware of an SAE or Notable Event, please email a completed form to the MRC/UVRI and LSHTM on:
PrEPVacc_trial_safety@mrcuganda.org

7.4 MRC/UVRI AND LSHTM RESPONSIBILITIES

Medically-qualified staff at MRC/UVRI and LSHTM and/or the Coordinating Investigator (or a medically-qualified delegate) will review all SAE reports received and will coordinate the review by the TSG (see **section 14.2**). The causality assessment given by the local investigator can be queried, but not overruled; in the case of disagreement, both opinions will be provided in any subsequent reports.

MRC/UVRI and LSHTM is undertaking the duties of trial Sponsor and is responsible for assisting national PIs to inform their national regulatory authorities and research ethics committees, within the stipulated timelines.

MRC/UVRI and LSHTM will also keep all investigators informed of any safety issues that arise during the course of the trial, including updates to the reference safety information, once approved by the authorities.

MRC/UVRI and LSHTM, as Sponsor delegate, will oversee submission of Annual Safety Reports to Competent Authorities (Regulatory Authority and Ethics Committee).

Product providers involved will also be notified of all SAEs. MRC/UVRI and LSHTM will also provide product providers with a copy of the Annual Safety Report in the required format.

8 QUALITY ASSURANCE & CONTROL

8.1 RISK ASSESSMENT

The Quality Assurance (QA) and Quality Control (QC) considerations have been based on a formal Risk Assessment, which acknowledges the risks associated with the conduct of the trial and how to address them with QA and QC processes. QA includes all the planned and systematic actions established to ensure the trial is performed and data generated, documented and/or recorded and reported in compliance with the protocol and the principles of GCP and applicable regulatory requirements. QC includes the operational techniques and activities done within the QA system to verify that the checks are run and action taken when necessary. This Risk Assessment is reviewed by the MRC CTU's Research Governance Committee and has informed the development of a Data Management Plan, a SMP and Monitoring Plan which is separately reviewed by the MRC CTU Quality Management Advisory Group.

8.2 CENTRAL MONITORING AT MRC/UVRI AND LSHTM

Each site will be responsible for its own data entry into the central PrEPVacc database and local trial management. The site will retain the original CRF. Data stored on the central database will be checked by personnel authorised by MRC/UVRI and LSHTM, MRC CTU at UCL and IAVI for missing or unusual values (range checks) and checked for consistency within participants over time. If any such problems are identified, the site will be contacted and asked to verify or correct the entry. Changes will be made on the original CRF and entered into the database at the site. MRC/UVRI and LSHTM will also send reminders for any overdue and/or missing data with the regular inconsistency reports of errors.

Other essential trial issues, events and outputs will be detailed in the Monitoring Plan that is based on the trial-specific Risk Assessment.

8.3 ON-SITE MONITORING

Staff from IAVI and MRC/UVRI and LSHTM or MRC CTU at UCL will visit clinical sites to validate and monitor data and an independent local monitor will make regular visits to the trial sites. The frequency, type and intensity for routine monitoring and the requirements for triggered monitoring will be detailed in the Monitoring Plan. This plan will also detail the procedures for review and sign-off.

8.3.1 DIRECT ACCESS TO PARTICIPANT RECORDS

Participating investigators should agree to allow trial-related monitoring, including audits, ethics committee review and regulatory inspections by providing direct access to source data and documents as required. Participants' consent for this must be obtained.

8.3.2 CONFIDENTIALITY

We plan to follow the principles of the European General Data Protection Regulation 2016/679 (GDPR) and comply with national regulations in the countries where the trial is being conducted.

9 STATISTICAL CONSIDERATIONS

9.1 METHOD OF RANDOMISATION

There are two separate randomisations applied concurrently in PrEPVacc:

For the vaccine part of the trial participants will be randomised 1:1:1 to either

- A: DNA-HIV-PT123 and AIDSVAX® B/E (target wks 0,4,24,48)
- B: DNA-HIV-PT123 and CN54gp140+MPLA-L (target wks 0,4), then MVA-CMDR and CN54gp140+MPLA-L (target wks 24,48)
- C: Saline placebo (target wks 0,4,24,48)

For the PrEP part of the trial participants will be randomised 1:1 to either

- A: TDF/FTC once daily from week 0 until visit 9 (CONTROL)
- B: TAF/FTC once daily from week 0 until visit 9 (EXPERIMENTAL)

Both randomisations will be stratified by centre and gender using a web-based system controlled through an authorised user name and password, at each site. Before each allocation, the participant's eligibility will be confirmed and the stratification factor entered into the database. Randomisation lists for both randomisations will be generated by an independent statistician not involved in the day to day running of the study.

In the event the ratio need to change to 1:1 during the trial, a second randomisation list will be implemented and the first randomisation database archived (see **Section 4.2**). A third list will be prepared in order to be ready for a return to 1:1:1.

9.2 OUTCOME MEASURES

9.2.1 VACCINE TRIAL:

9.2.1.A Primary

- HIV acquisition by a participant who completed first three immunisations and was HIV negative 2 weeks after the third immunisation
- A clinical decision to discontinue the vaccine regimen for an adverse event that is considered related to product

9.2.1.B Secondary

- Grade 3 and above solicited adverse events which last more than 72 hours
- Grade 3 and above laboratory adverse events that are confirmed on repeat testing if possible
- An adverse event leading to a clinical decision to discontinue or interrupt the product schedule, regardless of relationship
- Serious Adverse Events (SAEs)
- Other clinical and laboratory adverse events
- Binding antibodies to CN54gp140 and AIDSVAX® B/E gp120

9.2.1.C Exploratory

- CD4 and CD8 T-cell responses measured by multiparameter flow cytometry
- Neutralizing antibody responses
- Binding antibody responses (including V1/V2 regions) measured by binding antibody multiplex assay (BAMA)

- ADCC and other functional antibody responses

9.2.2 PrEP TRIAL:

9.2.2.A Primary

- HIV acquisition at or before visit 9 by a participant who was HIV negative at enrolment
- A clinical decision to discontinue the PrEP regimen for an adverse event that is considered related to product

9.2.2.B Secondary

- An adverse event leading to a clinical decision to discontinue or interrupt the product schedule, regardless of relationship
- Serious adverse events (SAEs)
- Genotypic resistance at HIV seroconversion, focussing on the mutations selected by tenofovir and emtricitabine (codons 65, 70, 184 in reverse transcriptase).
- Adherence assessed by (a) self-report (b) results of point of care urine tests (c) TFV-DP levels measured on DBS in red blood cells (d) total number of pills dispensed

9.3 SAMPLE SIZE

9.3.1 VACCINE EFFICACY

The vaccine trial is powered for vaccine efficacy and numbers calculated using nstage software for MAMS design[47] based on 3 arms and 2 stages. Assuming an incidence rate of HIV-1 infection of 4/100pyrs and a loss to follow up of 10%, then 1668 participants (556 per group) would generate sufficient end-points to detect a protective efficacy of 70% relative to placebo at the final analysis. Should the incidence be higher than 4/100pyrs and depending on accrual rate it is possible that fewer than 556 per group would be required. Should incidence be lower, a longer period of follow-up may be required to accumulate the necessary number of endpoints.

There is one planned formal interim analysis to assess vaccine efficacy. This will be conducted when there are approximately 7 events (HIV infections) in the control (placebo) arm, occurring in individuals that have completed their first three immunisations and are HIV negative 2 weeks after the third immunisation . Results of this interim analysis will be reviewed by the IDMC to consider which arms have demonstrated sufficient efficacy to warrant further investigation. Full details will be provided in the Statistical Analysis Plan (SAP).

Table 10: Maximum numbers of patients per comparison group for approximately 90% power, 5% 2-sided significance overall

Efficacy (%) (E)	*Incidence /100py*	n events observed in control arm for interim analysis	N participants per group	Assuming 10% loss of PY
**50**	*3.0*	18	919	1021
	4.0	18	805	894
	5.0	18	713	792
**70**	*3.0*	7	561	623
	4.0	7	500	556
	5.0	7	434	482
**80**	*3.0*	4	461	512
	4.0	4	399	443
	5.0	4	357	397

9.3.2 PrEP COMPARISON

The PrEP study aims to show the effectiveness of TAF/FTC is not unacceptably lower than the effectiveness of TDF/FTC, assessed from the observed lower confidence limit for the Averted Infections Ratio (AIR).[48] Table 11 shows the probability (power) that the 5% lower confidence limit for the AIR will exceed either 50%, 60%, or 70% as a function of (a) the effectiveness of TDF/FTC, and (b) the hypothetical placebo incidence. The calculations assume (a) that TAF/FTC and TDF/FTC are equally effective (the standard assumption in PrEP non-inferiority trials), and (b) 10% loss of PYRS follow-up (as in Section 9.3.1.).

Table 11: Probability (%) that lower 5% confidence limit for AIR exceeds certain critical non-inferiority margins

Non-inferiority margin (%)	Effectiveness (%) of TDF/FTC	Placebo incidence (per 100 PY)		
		**3.0**	**4.0**	**5.0**
**50**	**50**	31	38	44
**50**	**60**	47	56	64
**50**	**70**	68	78	86
**50**	**80**	90	96	98
**60**	**50**	22	26	30
**60**	**60**	31	38	44
**60**	**70**	46	56	64
**60**	**80**	70	80	88
**70**	**50**	15	17	19
**70**	**60**	20	24	27
**70**	**70**	29	35	40
**70**	**80**	45	54	62

Table 11 shows there is reasonable power of showing at least 50% preservation of effect provided that TDF/FTC effectiveness is at least 70%, underlinng the importance of optimising adherence to PrEP. Demonstrating at least 60% preservation of effect is more challenging, while demonstrating at least 70% preservation of effect is very unlikely.

9.4 INTERIM MONITORING & ANALYSES

An IDMC Charter will be drawn up that describes the membership of the IDMC, relationships with other committees, terms of reference, decision-making processes, and the timing and frequency of interim analyses (with a description of stopping rules and/or guidelines, if any).

The IDMC will regularly review safety data and incident HIV data by randomised arm as described in the charter (for both randomisations).

In addition for the vaccine trial, there is one formal planned interim analyses to be performed when approximately 7 HIV incident cases are observed in the control arm. Recommendation for possible discontinuation of further enrolments and vaccines for those already enrolled to intervention arm(s) will be made from the results of this analysis.

9.5 ANALYSIS PLAN (BRIEF)

The analyses will be described in detail in a full SAP. This section summarises the main issues.

9.5.1 VACCINE TRIAL EFFICACY ANALYSES

For the Primary efficacy endpoint analysis, a modified intent-to-treat (mITT) approach will be adopted whereby participants randomised but later found to be HIV positive at or before the timepoint 2 weeks after the third immunisation will be excluded from the primary analysis.

A time-to event analysis will be adopted to estimate a hazard ratio for each pairwise comparisons (i.e. each active vaccine arm against placebo). Participants who are lost to follow-up will contribute person-years up to their last clinical follow-up. HIV incidence rates will be calculated for each allocation and the effect on HIV incidence will be determined using Cox Proportional Hazards models adjusting for site. Product efficacy will be expressed as 1 minus the hazard ratio for each active vaccine arm when compared to placebo.

9.5.2 PREP STUDY ANALYSES

The primary analysis will be mITT i.e. including all randomised participants but excluding those found to be HIV positive at enrolment. The incidence rate of new HIV infections, up to and including visit 9, will be computed for each randomised arm. The two rates will be directly compared using the rate difference and the rate ratio, and confidence intervals derived using the Poisson distribution. In addition, the Averted Infections Ratio (AIR) will be calculated based on: (a) an estimate of the hypothetical placebo incidence from the registration cohort (b) an estimate of the hypothetical placebo incidence based on follow-up of trial participants after visit 9 (c) the estimated effectiveness of TDF/FTC based on adherence data (d) a synthesis of (a), (b) , and (c). The lower 5% confidence limit will provide a lower bound estimate of the proportion of infections averted by TAF/FTC relative to TDF/FTC. No formal significance tests of non-inferiority will be conducted since the definition of the non-inferiority margin is largely arbitrary.

If there are sufficient HIV endpoints, an analysis stratified by gender will also be conducted.

If it transpires that neither vaccine arm confers any protection, then the effect of vaccination can be ignored in the PrEP analysis. However, if some level of protection is demonstrated then statistical models will be developed to model jointly the effects of PrEP and the vaccine regimens. This is

pertinent to the calculation of the AIR since the estimate of the hypothetical placebo incidence from the registration cohort would be biased upwards.

9.5.3 ANALYSIS OF QUALITATIVE DATA

Qualitative data analysis will be done using a Framework analysis approach. Following identification of themes, indexing (coding) and charting (copying and pasting data according to thematic areas) will be undertaken. Mapping (visual display of data) will then be done to allow researchers to identify patterns, associations, and concepts.

10 ANCILLARY STUDIES

There are no planned ancillary studies.

11 REGULATORY & ETHICAL ISSUES

All regulatory requirements (including safety reporting, **Section 7** and below) will be met by the sponsor or their delegated authorities. The sites will adhere to the requirements for collection of informed consent as set out in their national legislation

11.1 COMPLIANCE

11.1.1 Regulatory Compliance

The trial complies with the principles of the 1996 version of the Declaration of Helsinki.

This clinical trial involves Investigational Medicinal Products (IMP) and will be conducted in compliance with the approved protocol, the principles of GCP as laid down by the ICH topic E6 (Note for Guidance on GCP), by the European General Data Protection Regulation (GDPR) 2016/679, and national legislation in the participating countries relevant to clinical trials and protection of data collected for the purpose of research for public benefit.

11.1.2 Site Compliance

The site will comply with the above and an agreement will be in place between the site and the Sponsor, setting out respective roles and responsibilities (see **Section 13 - Finance**).

The site will inform MRC/UVRI and LSHTM as soon as they are aware of a possible serious breach of compliance, so that the Trials Coordinating Centre can report this breach within the timelines required by the regulatory authorities. For the purposes of this, a 'serious breach' is one that is likely to affect to a significant degree:

- The safety or physical or mental integrity of the subjects in the trial, or
- The scientific value of the trial

11.1.3 Data Collection & Retention

The central study database will be set up in OpenClinica, a web-based data management system, and hosted on a secure server at MRC/UVRI and LSHTM, Entebbe, Uganda. The database will be backed-up automatically on a daily basis. The database will be password protected and access will be limited to relevant study staff.

CRFs, clinical notes and administrative documentation should be kept in a secure location (for example, locked filing cabinets in a room with restricted access) and held for a minimum of 15 years after the end of the trial. During this period, all data should be accessible, with suitable notice, to the competent or equivalent authorities, the Sponsor, and other relevant parties in accordance with the applicable regulations. The data may be subject to an audit by the competent authorities.

Qualitative data will be kept on password protected computers. The dataset from the study will be kept on a secure computer server at each site. Transcripts will be pseudo-anonymized at the first stage of analysis with all identifying material removed from the transcripts and pseudonyms will be used. Age in years, gender and pattern of risk and adherence behaviours will be retained. Only authorised study staff will have access to the data. The hard copies of all forms used to write down information will be destroyed after entry on the computer.

11.1.4 Sample Storage and Retention

Samples for which participants have consented will be stored and analysed for the purposes outlined in the relevant sections of the PrEPVacc protocol. Destruction of left over or unused sample will be carried out as per the local country requirements and regulations relating to the disposal of biological research samples.

Samples will be managed as stipulated in the PrEPVacc Laboratory Analytical Plan. Samples will be stored securely at the local laboratory/biorepository and some will be transferred to the study central biorepository at MRC/UVRI and LSHTM Uganda Research Unit in Entebbe, Uganda. Stored samples may be shipped to other laboratories or institutions for testing. Any additional research that will be performed using the stored/remaining samples will be reviewed and approved by the relevant Institutional Review Board or Ethics Committee.Stored samples for future use may be stored indefinitely.

11.2 ETHICAL CONDUCT

11.2.1 ETHICAL CONSIDERATIONS

PrEPVacc is assessing interventions to reduce the risk of HIV acquisition and therefore sites will be recruiting individuals at risk of acquiring HIV. Populations at risk of HIV are frequently vulnerable because of the intersecting social syndemics of poverty, violence, drug and alcohol addiction, and depression. In PrEPVacc participants will be provided with condoms and PrEP, both of which are known to prevent HIV, but the study supply of PrEP will cease once they have completed three injections plus two weeks follow-up. Study staff will inform participants where they can access PrEP locally and support them to do so where this is possible, and continue to supply condoms. Please see section 1.5 for context and rationale.

The vaccines are not licensed and some have only been administered to a few hundred individuals so there may be adverse events that we have not anticipated.

Participants who receive active vaccine may develop antibodies that lead to a positive point of care HIV test in future, including after the trial has finished. Study staff will provide participants with an identity card verifying their participation in a HIV vaccine trial and continued access to laboratory testing and certification of status.

The materials to support informed consent will be developed to ensure that participants understand the risks and benefits of participation, in particular that the design includes a placebo vaccine, that no-one knows whether the active vaccines can reduce the risk of HIV, that they may require additional tests after the study to distinguish between a vaccine reaction and HIV infection, and that study will only supply PrEP for 26 weeks. The materials developed will take account of the level of literacy in each centre. Participants who are not able to read will be asked to provide their thumbprint. They will be accompanied by a witness of their choice who can read and who will be asked to sign the consent form.

11.2.2 FAVOURABLE ETHICAL OPINION

Before initiation of the trial at each clinical site, the protocol, all informed consent forms, and information materials to be given to the prospective participant will be submitted to the local and national ethics committees in each country for review. Approval of the protocol and key messages in the informed consent materials will also be sought from the Imperial College Ethics Committee. The opinion of the national ethics committee in each country will determine the content of the informed consent materials given to participants in that country.

The study has been developed with Participant and Public Involvement (PPI) to ensure that its design is feasible and acceptable to potential participants, and to ensure its outcomes and potential impact are relevant to the population who may benefit from its results. Each study site has a Community Advisory Board and will seek their assistance to actively involve participants when the trial has begun to help to ensure transparency and accountability throughout the research. Prior to the trial starting, participants in the Registration Cohort will be encouraged to provide feedback on the design, the study procedures and the draft materials for informed consent. A Community

representative has agreed to join the Trial Steering Committee (see **section 14.2**). PPI activity will continue for the duration of the study, including dissemination of study results.

The rights of the participant to refuse to participate in the trial without giving a reason will be respected.

11.3 COMPETENT AUTHORITY APPROVALS

This protocol and supporting documents will be reviewed by the national regulatory authorities and ethics committees in each participating country prior to implementation of the trial.

The progress of the trial and safety issues will be reported to the competent authorities and ethics committees that have provided approval in accordance with national requirements and practices in a timely manner.

Interim updates on the products will be provided if there are emergent safety data. In the scenario of any quality issue with any one product that requires further investigation, further injections of this product will be paused (see **Section 5.2.7** Dose Modifications & Interruptions) and the competent authorities will be informed. Injections of the product in question will only be resumed if the outcome of the investigation supports the further use of the product and upon approval from the competent authorities.

Safety reports, including expedited reporting and SUSARS will be submitted to the competent authority in accordance with each authority’s requirements in a timely manner.

11.4 OTHER APPROVALS

The protocol will be submitted by those delegated to do so to the relevant institutional review boards of each participating site or to other local stakeholders as required and in accordance with usual practice.

12 INDEMNITY

Imperial College, London will provide global clinical trial insurance for the study. However as this policy is governed by UK law, an additional policy complying with in-country requirements will be put into place by Imperial College in each of the countries prior to the start of recruitment.

13 FINANCE

This trial has been funded by the European and Developing Countries Clinical Trial Partnership (EDCTP2) under grant reference RIA2016V-1644.

Imperial College, London is the sponsor for the trial which is being co-ordinated by MRC/UVRI and LSHTM Uganda Research Unit. A Clinical Trial Collaboration Agreement between all parties will be in place designating roles and responsibilities.

14 OVERSIGHT & TRIAL COMMITTEES

There are a number of committees involved with the oversight of the trial. These committees are detailed below, and the relationship between them expressed in the figure below.

Figure 2 Trial teams and Oversight Committees and Relationships

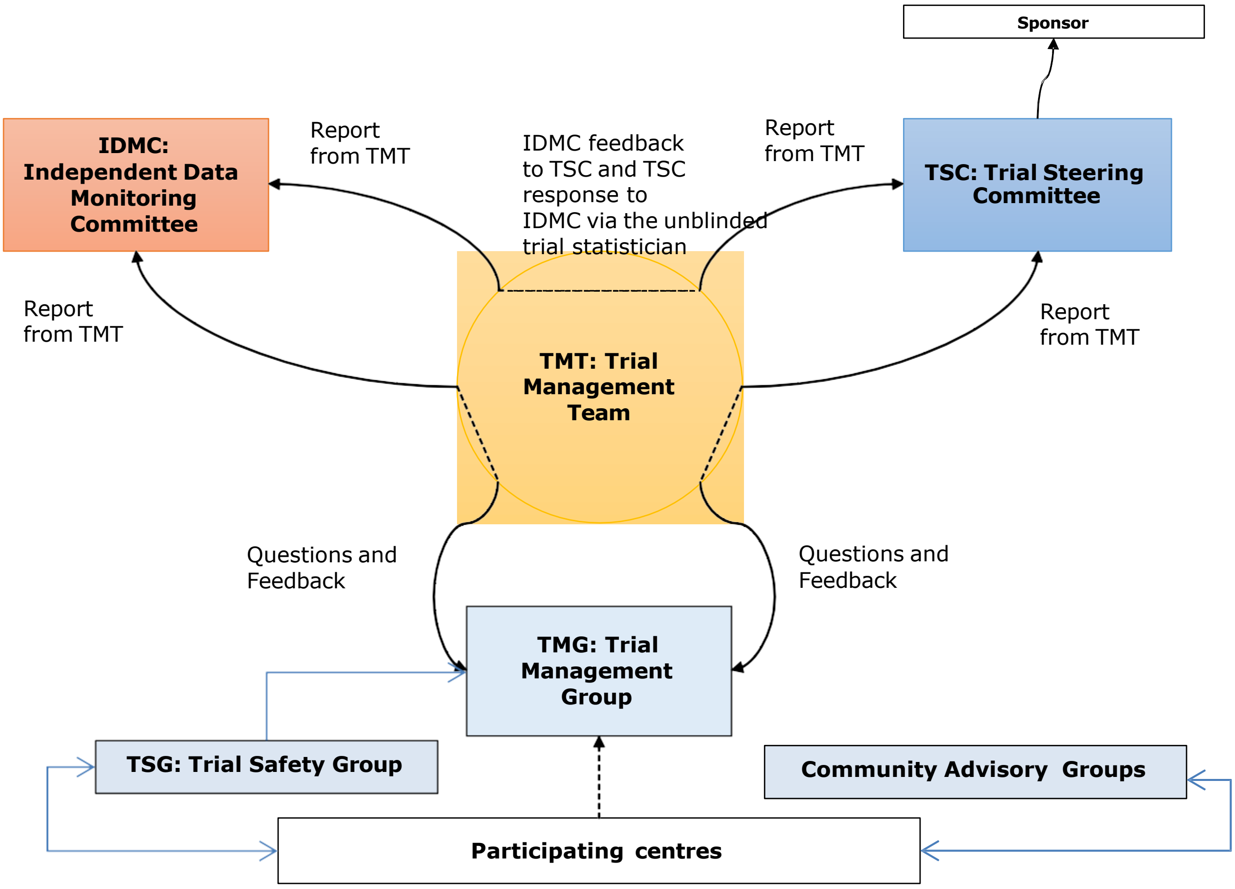

14.1 TRIAL MANAGEMENT GROUP (TMG)

A Trial Management Group (TMG) will be formed comprising the Coordinating Investigator, other lead investigators (clinical and non-clinical), members of the MRC/UVRI and LSHTM Uganda Research Unit Trial Coordinating Centre and the MRC Clinical Trials Unit (CTU) at UCL and community contributors. The TMG will be responsible for the day-to-day running and management of the trial. It will meet approximately once a month usually by teleconference. The full details can be found in the TMG Charter.

14.2 TRIAL SAFETY GROUP (TSG)

The Trial Safety Group (TSG) includes medically-qualified staff at MRC/UVRI and LSHTM Uganda Research Unit who are not assessing participants directly, medically qualified staff at MRC CTU at UCL including the Trial Physician, Ludwig Maximillians University, IAVI and EuroVacc (or a medically-qualified delegate), and representatives from the other product providers. The TSG is responsible for reviewing SAEs, notable events, interruptions and discontinuations. The role of the TSG will be described in the trial SMP.

14.3 TRIAL MANAGEMENT TEAM (TMT)

A Trial Management Group (TMT) will be formed comprising the Chair of the TMG, members of the MRC/UVRI and LSHTM Uganda Research Unit, and others with coordinating operational responsibilities such as members of MRC CTU at UCL, Imperial College, IAVI and EuroVacc. The full details can be found in the TMG Charter.

14.4 TRIAL STEERING COMMITTEE (TSC)

The Trial Steering Committee (TSC) has independent members, including the Chair and community representatives as well as selected members of the TMG. The role of the TSC is to provide overall supervision for the trial and provide advice through its independent Chair to the TMG and the Sponsor. Reports for the TSC will be prepared by the Trial Coordinating Centre. The ultimate decision for the continuation of the trial lies with the TSC, although exceptionally the Sponsor may act independently of the TSC to stop the trial. Further details of TSC functioning are presented in the TSC Charter.

14.5 INDEPENDENT DATA MONITORING COMMITTEE (IDMC)

An Independent Data Monitoring Committee (IDMC) will be formed. The IDMC will be the only group who sees the confidential, accumulating unblinded data for the trial. Reports to the IDMC will be produced by the unblinded trial statistician (who is based at MRC CTU at UCL) with support as needed from other members of the TMT. Recommendations from the IDMC to the TSC, and responses from the TSC to the IDMC, are relayed through the unblinded trial statistician.. The IDMC will meet regularly during the trial and the frequency of meetings will be dictated in the IDMC charter. The IDMC will consider data using the statistical analysis plan (see **Section 9.5**) and will advise the TSC. The IDMC can recommend premature closure or reporting of the trial, or that recruitment to any research arm be discontinued.

Further details of IDMC functioning and the procedures for interim analysis and monitoring are provided in the IDMC Charter.

14.6 PARTICIPANT AND PUBLIC INVOLVEMENT ADVISORY GROUPS

Each participating centre has an established Community Advisory Group/Board composed of stakeholders that represent the local community as well as local health care providers and allied organisations. In some instances individuals who have participated in previous studies are members.

Prior to implementation, the study design and procedures will be discussed with these local groups, questions answered, and their advice sought with respect to any modifications to facilitate recruitment and retention.

Progress will be reported to the Community Advisory Group/Board during the conduct of the study, issues arising will be aired and the plan to address these formulated. These meetings are likely to follow the formal reports to the Trial Steering Committee. There will also be a meeting just before dissemination of the results to ensure that the key messages are clear and the impact on the community fully explored.

14.7 ROLE OF STUDY SPONSOR

The study will be sponsored by Imperial College, London with responsibilities defined by a written clinical trial agreement with collaborators.

15 PARTICIPANT AND PUBLIC INVOLVEMENT

Participant and Public Involvement (PPI) in research is defined as research being carried out 'with' or 'by' members of the public rather than 'to', 'about' or 'for' them.

The Good Participatory Practice Guidelines for HIV prevention trials[49] provide a framework for effective stakeholder engagement which is based on the principles of respect, mutual understanding, integrity, transparency, accountability, and community stakeholder autonomy.

15.1 PPI STRATEGY

PrEPVacc will have a comprehensive PPI strategy which will involve participants, the public and key community stakeholders throughout the life-cycle of the trial. At the core of the strategy will be the commitment to active PPI during the study, building partnerships with participants, the public, local and international stakeholders to shape decisions about the research. The term 'participant and public' includes former participants, people who use local health care and social services and people who represent those services.

The goal is to gain a broad and diverse range of perspectives rather than achieve representativeness of specific population groups.

The strategy will describe

- the models of PPI that will be used in the PrEPVacc trial
- the use of PPI through the research cycle of the trial
- the framework for assessing the impact of PPI
- the procedures for supporting PPI

15.2 PPI CONTRIBUTORS

The majority of PPI contributors will participate in the Community Advisory Group/Board local to each centre. A smaller number of individuals with experience of research will be invited to participate in the oversight and trial committees described in section 14, such as the Trial Management Group and the Trial Steering Committee.

The types of issues that views will be sought on include

- The protocol
- The information sheet and consent and supporting materials
- The recruitment and retention strategy including advertising materials
- The results and key messages

15.3 REPORTING AND EVALUATING IMPACT OF PPI

An impact assessment form will be used to collate the purpose, implementation and response for each PPI activity throughout the course of the trial. If recommendations from PPI activity are not implemented, the reason why will be documented on the assessment form.

16 PUBLICATION AND DISSEMINATION OF RESULTS

The preparation of a manuscript for publication in a peer-reviewed professional journal or an abstract for presentation, oral or written, to a learned society or symposium will be discussed on the Trial Management Group calls.

Authorship should reflect work done by the investigators and other personnel involved in the analysis and interpretation of the data, in accordance with generally recognised principles of scientific collaboration.

Details regarding the roles and responsibilities and timelines are contained in the PrEPVacc Consortium Agreement and will be reflected in the Clinical Trial Agreement.

17 DATA AND/OR SAMPLE SHARING

Data will be shared according to a controlled access approach, based on the following principles:

- No data should be released that would compromise an ongoing trial or study.
- There must be a strong scientific or other legitimate rationale for the data to be used for the requested purpose.
- Investigators who have invested time and effort into developing a trial or study should have a period of exclusivity in which to pursue their aims with the data, before key trial data are made available to other researchers.
- The resources required to process requests should not be under-estimated, particularly successful requests which lead to preparing data for release. Therefore adequate resources must be available in order to comply in a timely manner, and the scientific aims of the study must justify the use of such resources.
- Data exchange complies with Information Governance and Data Security Policies in all of the relevant countries.

Researchers wishing to access data should contact the Trial Management Group in the first instance using the trial administration email PrEPVacc-MRC@mrcuganda.org.

18 PROTOCOL AMENDMENTS

After the protocol has been approved by the national ethics committees and regulatory authorities, no changes may be made without the documented agreement of both the investigators and the Sponsor.

VERSION	DATE	AMENDMENT N°	MAIN MODIFICATIONS
2.0	04 Sept 2019	1	• NCT number added (on cover and in text)<br>• Weight added to schedule weeks -8, 0, 16, 26, 48<br>• STI tests after week 26 amended to "only if indicated" on schedule<br>• DBS updated (on schedule and in section 6.6) to be taken and stored at weeks 48, 74 and every 24 weeks thereafter (4ml added to total sample volume)<br>• DBS and TDF added to abbreviations<br>• Index updated accordingly<br>• Details TAF/FTC as PrEP added to section 1.4.6<br>• Number of participants changed from "upto 1668" to "a minimum of 1668" to allow for extras if replacements needed<br>• Sections 5.2.4, 5.3.6 and 6.3.3 updated to ease and clarify operational actions<br>• Packaging of AIDSVAX and MVA updated<br>• Under secondary safety outcomes in summary and section 9.2.1.B "Grade 3 and worse solicited clinical and laboratory adverse events" updated to "Grade 3 and above solicited adverse events which last more than 72 hours and Grade 3 and above laboratory adverse events that are confirmed on repeat testing if possible" to be consistent with section 7.2<br>• References 47 and 48 details expanded<br>• Minor formatting changes
3.0	12 Nov 2019		• Summary updated to clarify objectives<br>• Objectives added to Section 1.5<br>• Eligibility for Creatinine clearance/estimated glomerular filtration rate updated to exclude anyone with <60ml/min throughout, and clarifications to clinical monitoring of PrEP drugs added to 6.5.3

4.0	11 June 2020	2	• Summary updated to clarify IDMC safety & effectiveness review and number of participants<br>• Objective 4 added to summary and section 1.5.1<br>• Schema (Figure 1) updated to reflect timepont change -6 to -8 and vaccine analysis<br>• Table 1 schedule updated to reflect changes in weight and blood timepoints, blood volumes (minor reductions) and visit days allowances<br>• Section 1.2 – results of HVTN702 updated and results reference added<br>• Section 1.4.6 TAF/FTC results updated<br>• Section 2.1 capacity building added<br>• Sections 5.2.3, 5.3.5 and 5.4.2 amended to allow extra vaccines in case of droput<br>• Vaccine concentrations updated in selected sections<br>• Section 6.1 blood volumes corrected<br>• Table 7 – discomfort replaced by pain/tenderness and blisters deleted to as this is covered under other events<br>• Section 6.7.1 updated to clarify HIV test result reporting and to clarify the breakdown of the 61ml extra blood<br>• Section 11.1.3 updated to clarify data collection<br>• Section 11.1.4 updated to provide further detail on samples<br>• Section 14.5 and figure 2 updated to confirm blinding<br>• Pharmacy plan replaced with study operations manual (SOM) throughout<br>• Minor amendments and corrections have been made throughout to clarify discrepancies

5.0	29 September 2021		• Summary and elsewhere updated as Mozambique will not take part in the trial and to clarify that documents (details of trial team, participant information sheets, informed consent form and toxicity table) are no longer appended, but submitted as separate documents<br>• Summary, schema and Table 1 amended to clarify flexibility with windows and emphasise visit number over weeks for alignment to procedures<br>• Section 1.51l5 updated to reflect new ethical guidelines for HIV prevention trials and current access to PrEP in each site<br>• Sections 4.2, 5.2.7, 5.7, 6.2.1 and 9.1 updated to better describe the adaptive nature of trial and how a recommendation to interrupt one combination arm would be implemented<br>• Section 6 updated to clarify that paritcipants may be enrolled directly in the trial without enrolling in the Registration Cohort first<br>• Section 6.2.1 corrected to clarify Sponsor will not have a copy of informed consent<br>• Table 7 updated with timing for collection of solicited adverse events (AE) and to remove night sweats which will be collected as other AE instead

19 REFERENCES

1. http://www.unaids.org/sites/default/files/media_asset/unaids-data-2018_en.pdf accessed 13December 2018
2. Flynn, N.M., Forthal, D.N., Harro, C.D. et al Placebo-controlled phase 3 trial of a recombinant glycoprotein 120 vaccine to prevent HIV-1 infection *J Infect Dis* 2005;191:654-65
3. Pitisuttithum P., Gilbert P., Gurwith M., et al Randomised double-blind placebo-controlled efficacy trial of a bivalent recombinant glycoprotein 120 HIV-1 vaccine amont injection drug users in Bangkok Thailand *J Infect Dis* 2006;194:1671-71
4. Buchbinder SP., Mehrotra DV., Duerr A., et al Efficacy assessment of a cell-mediated immunity HIV-1 vaccine (the Step study): a doubl-blind, randomized, placebo-controlled, test of concept trial. *Lancet* 2008 372 (9653):1881-1893
5. Gray GE., Allen M., Moodie Z., et al Safety and efficacy assessment of the HVTN 503/Phambili Study: A double-blind randomized placebo-controlled test of concept study of a Clade B-based HIV-1 vaccine in South Africa *Lancet Infect Dis* 2011:11(7):507-515
6. Hammer SM., Sobieszczyk ME., Janes H., et al Effiacy trial of a DNA/rAd5 HIV-1 preventive vaccine N Eng J Med 2013;369:2083-2092
7. Rerks-Ngarm S., Pitisuttithum P., Nitayaphan S., et al. Vaccination with ALVAC and AIDSVAX to prevent HIV-1 infection in Thailand. *N Engl J Med* 2009 Dec 3;361(23):2209–20
8. Haynes BF, Gilbert PB, McElrath MJ, et al. Immune-correlates analysis of an HIV-1 vaccine efficacy trial. *N Engl J Med*. 2012 Apr 5;366(14):1275–86.
9. Zolla-Pazner S, deCamp A, Gilbert PB, et al. Vaccine-induced IgG antibodies to V1V2 regions of multiple HIV-1 subtypes correlate with decreased risk of HIV-1 infection. *PloS One*. 2014;9(2):e87572
10. press release: https://www.niaid.nih.gov/news-events/experimental-hiv-vaccine-regimen-ineffective-preventing-hiv
11. ClinicalTrials.gov Identifier: NCT03060629
12. Grant R., Lama JT, Anderson PL, et al Preexposure chemoprophylaxis for HIV prevention in men who have sex with men *N Eng J Med* 2010 363 2587-99
13. Baeten JM., Donnell D., Ndase P., et al. Parnter PrEP Study Team. Antiretroviral prophylaxis for HIV prevention in heterosexual men and women *N Eng J Med* 2012;367:399-410
14. Thigpen MC, Kebaabetswe PM, Paxton LA, Smith DK, Rose CE, Segolodi TM, Henderson FL, Pathak SR, Soud FA, Chillag KL, Mutanhaurwa R, Chirwa LI, Kasonde M, Abebe D, Buliva E, Gvetadze RJ, Johnson S, Sukalac T, Thomas VT, Hart C, Johnson JA, Malotte CK, Hendrix CW, Brooks JT; TDF2 Study Group. 2012. Antiretroviral preexposure prophylaxis for heterosexual HIV transmission in Botswana. *N Engl J Med*. 2012 Aug 2;367(5):423-34
15. McCormack S., Dunn D., Desai M., et al. Pre-exposure prophylaxis to prevent the acquisition of HIV-1 infection (PROUD): effectiveness results from the pilot phase of a pragmatic open-label randomized trial. Lancet 2016;387:53-60
16. Molina JM., Capitant C., Spire B., et al. On-demand preexposure prophylaxis in men at high risk for HIV-1 infection *N Eng J Med* 2015;373:2237-46
17. Baeten J., Grant R., McCormack S., et al HIV incidence in persons using TDF/FTC for HIV pre-exposure prophylaxis (PrEP): worldwide experience from 46 studies. *HIVR4P 2018* 21-25 October 2018; Abstract OA
18. Guideline on when to start antiretroviral therapy and on pre-exposure prophylaxis for HIV World Health Organization 2015; ISBN 978 92 4 1509565
19. J.K. Kyongo1, M. Kiragu2, R. Karuga1, et al How long will they take it? Oral pre-exposure prophylaxis (PrEP) retention for female sex workers, men who have sex with men and young women in a demonstration project in Kenya *AIDS 2018* 23 - 27 July 2018;Abstract WEAE0403
20. Fitch L et al. Tracking global oral PrEP provision: the who, what and where of oral PrEP. *HIVR4P 2018* 21-25 October 2018; Abstract OA04.01

21. Hojilla JC, Vlahov D, Crouch PC, et al HIV pre-exposure prophylaxis (PrEP) uptake and retention among men who have sex with men in a community based sexual health clinic. *AIDS Behav* 2018; 22: 1096–9
22. Chan PA., Mena L., Patel R., et al. Retention in care outcomes for HIV pre-exposure prophylaxis implementation programmes among men who have sex with men in three US cities. *J Int AIDS Soc* 2016; 19: 20903
23. Grulich A., Guy R., Amin J., et al. Population-level effectiveness of rapid, targeted, high-coverage roll-out of HIV pre-exposure prophylaxis in men who have sex with men: the EPIC-NSW prospective cohort study. *Lancet HIV* 2018 Published Online October 17, 2018 http://dx.doi.org/10.1016/S2352-3018(18)30215-7
24. Pantaleo G, Holly J, Tomaras GD, et al. Comparing Different Priming Strategies to Optimize HIV Vaccine Antibody Responses: Results from HVTN 096/EV04. *HIVR4P 2016* October 2016; Abstract OA.
25. Pala P, Levin J, Nanvubya A, et al. EV06 Trial: Modulation of the Immunogenicity of the DNA-HIV-PT123 and AIDSVAX®B/E combination HIV vaccine in adult Ugandans by S. mansoni Infection *HIVR4P 2016* October 2016; Abstract OA.
26. Rouphael N, Morgan C, Li S, Jensen R, Sanchez B, Swann E, et al. DNA and Protein Co-administration Elicits Higher HIV-specific nAb and Comparable CD4+ T-cell Responses as Traditional Prime/Boost Regimens (HVTN 105). In: HIV Research for Prevention Conference. Chicago, US; 2016
27. Hosseinipour M, Innes C, Naidoo S, Tomaras GD, Morris L, DeCamp A, et al. Co-administration of 3 doses of DNA/Protein Improves Antibody Responses to a Prime-Boost Approach, but Needle-Free Injection of DNA Shows No Benefit. In: HIV Research for Prevention Conference. Madrid, Spain; 2018.
28. Nicole L. Yates, Hua-Xin Liao, Youyi Fong, Allan deCamp, Nathan A. Vandergrift, William T. Williams, S. Munir Alam, Guido Ferrari, Zhi-yong Yang, Kelly E. Seaton, Phillip W. Berman, Michael D. Alpert, David T. Evans, Robert J. O'Connell, Donald Francis, Faruk Sinangil, Carter Lee, Sorachai Nitayaphan, Supachai Rerks-Ngarm, Jaranit Kaewkungwal, Punnee Pitisuttithum, James Tartaglia, Abraham Pinter, Susan Zolla-Pazner, Peter B. Gilbert, Gary J. Nabel, Nelson L. Michael, Jerome H. Kim, David C. Montefiori, Barton F. Haynes, Georgia D. Tomaras. 2014.Vaccine-Induced Env V1–V2 IgG3 Correlates with Lower HIV-1 Infection Risk and Declines Soon After Vaccination Sci Transl Med. Author manuscript; available in PMC 2014 Sep 19
29. Cosgrove CA, Lacey CJ, Cope AV, Bartolf A, Morris G, Yan C, Baden S, Cole T, Carter D, Brodnicki E, Shen X, Joseph S, DeRosa SC, Peng L, Yu X, Ferrari G, Seaman M, Montefiori DC, Frahm N, Tomaras GD, Stöhr W, McCormack S, Shattock RJ. 2016 Comparative Immunogenicity of HIV-1 gp140 Vaccine Delivered by Parenteral, and Mucosal Routes in Female Volunteers; MUCOVAC2, A Randomized Two Centre Study. *PLoS One.* May 9;11(5):e0152038.
30. Joachim A, Bauer A, Joseph S, Geldmacher C, Munseri PJ, Aboud S, Missanga M, Mann P, Wahren B, Ferrari G, Polonis VR, Robb ML, Weber J, Tatoud R, Maboko L, Hoelscher M, Lyamuya EF, Biberfeld G, Sandström E, Kroidl A, Bakari M, Nilsson C, McCormack S. 2016. Boosting with Subtype C CN54rgp140 Protein Adjuvanted with Glucopyranosyl Lipid Adjuvant after Priming with HIV-DNA and HIV-MVA Is Safe and Enhances Immune Responses: A Phase I Trial. PLoS One. May 18;11(5)
31. Joseph S, Quinn K, Greenwood A, Cope AV, McKay PF, Hayes PJ, Kopycinski JT, Gilmour J, Miller AN, Geldmacher C, Nadai Y, Ahmed MI, Montefiori DC, Dally L, Bouliotis G, Lewis DJ, Tatoud R, Wagner R, Esteban M, Shattock RJ, McCormack S, Weber J. 2017. A Comparative Phase I Study of Combination, Homologous Subtype-C DNA, MVA, and Env gp140 Protein/Adjuvant HIV Vaccines in Two Immunization Regimes. Front Immunol. 2017 Feb 22;8:149
32. Viegas EO, Kroidl A Munseri P, Missanga MT, Nilsson C, Tembe N, Bauer A, Joachim A, Joseph S, Mann P, Geldmacher C, Fleck S, Stöhr W, Scarlatti G, Aboud S, Bakari M, Maboko L, Hoelscher M, Wahren B, Robb ML, Weber JN, McCormack S, Biberfeld G, Jani IV, Sandström E, Lyamuya E, TaMoVac study group. 2018 Optimizing the immunogenicity of HIV prime-boost DNA-MVA-

rgp140/GLA vaccines in a phase II randomized factorial trial design. PLoS One. 2018 Nov 29;13(11):e0206838.

33. Kratochvil S, McKay PF, Kopycinski JT, Bishop C, Hayes PJ, Muir L, Pinder CL, Cizmeci D, King D, Aldon Y, Wines BD, Hogarth PM, Chung AW, Kent SJ, Held K, Geldmacher C, Dally L, Santos NS, Cole T, Gilmour J, Fidler S, Shattock RJ. A Phase 1 Human Immunodeficiency Virus Vaccine Trial for Cross-Profiling the Kinetics of Serum and Mucosal Antibody Responses to CN54gp140 Modulated by Two Homologous Prime-Boost Vaccine Regimens. Front Immunol. 2017 May 24;8:
34. Volz A, Sutter G. 2013. Protective efficacy of Modified Vaccinia virus Ankara in preclinical studies. Vaccine. Sep 6;31(39):4235-40.
35. Currier JR, Ngauy V, de Souza MS, et al. Phase I safety and immunogenicity evaluation of MVA-CMDR, a multigenic, recombinant modified vaccinia Ankara-HIV-1 vaccine candidate. *PLoS One*. Nov 15 2010;5(11):e13983.
36. Sandström E, Nilsson C, Hejdeman B, et al. HIV Immunogenicity Study 01/02 Team.2008. Broad immunogenicity of a multigene, multiclade HIV-1 DNA vaccine boosted with heterologous HIV-1 recombinant modified vaccinia virus Ankara. *J Infect Dis* Nov 15 2008;198(10):1482-90
37. Nilsson C, Godoy-Ramirez K, et al. Broad and potent cellular and humoral immune responses after a second late HIV-modified vaccinia virus ankara vaccination in HIV-DNA-primed and HIV-modified vaccinia virus Ankara-boosted Swedish vaccinees. *AIDS Res Hum Retroviruses*. 2014 Mar;30(3):299-311
38. Bakari M, Aboud S, Nilsson C, et al. Broad and potent immune responses to a low dose intradermal HIV-1 DNA boosted with HIV-1 recombinant MVA among healthy adults in Tanzania. *Vaccine* Oct 26 2011;29(46):8417-28
39. Nilsson C, Hejdeman B, Godoy-Ramirez K, et al. HIV-DNA Given with or without Intradermal Electroporation Is Safe and Highly Immunogenic in Healthy Swedish HIV-1 DNA/MVA Vaccinees: A Phase I Randomized Trial. *PLoS One* 2015 Jun 29;10(6)
40. Munseri PJ, Kroidl A, Nilsson C, et al. Priming with a simplified intradermal HIV-1 DNA vaccine regimen followed by boosting with recombinant HIV-1 MVA vaccine is safe and immunogenic: a phase IIa randomized clinical trial. *PLoS One*. Apr 15 2015;10(4)
41. Ake JA, Schuetz A, Pegu P, et al. Safety and Immunogenicity of PENNVAX-G DNA Prime Administered by Biojector 2000 or CELLECTRA Electroporation Device With Modified Vaccinia Ankara-CMDR Boost. *J Infect Dis*. Nov 27 2017;216(9):1080-1090
42. Hare CB, Coll J, Ruane P, Molina J-M, Mayer KH, Jessen H, Grant RM, De Wet JJ, Thompson M, DeJesus E, Ebrahimi R, Mera Giler R, Das M, Brainard D, McCallister S 2019. The Phase 3 DISCOVER study: Daily F/TAF or F/TDF for HIV Pre-exposure Prophylaxis. Abstract No. 104. CROI, Seattle, Washington
43. Markowitz M, et al. Phase I/II study of the pharmacokinetics, safety and antiretroviral activity of tenofovir alafenamide, a new prodrug of the HIV reverse transcriptase inhibitor tenofovir, in HIV-infected adults. *J Antimicrob Chemother*. May 2014;69(5):1362-9.
44. Cottrell ML, Garrett KL, Prince HMA, et al. Single-dose pharmacokinetics of tenofovir alafenamide and its active metabolite in the mucosal tissues. *J Antimicrob Chemother*. 2017;72(6):1731-1740.
45. Schwartz JL, Thurman AR, Brache V, et al. HIV Prevention in Healthy Women: Safety and Pharmacokinetics of a Potential New Tenofovir Alafenamide Fumarate (TAF)-based Oral PrEP Regimen. *HIVR4P 2018* 21-25 October 2018; Abstract OA
46. Raphael N, Morgan C, Li S, et al. DNA and Protein Co-administration Elicits Higher HIV-specific nAb and Comparable CD4+ T-cell Responses as Traditional Prime/Boost Regimens (HVTN 105). *HIVR4P 2016* October 2016; Abstract OA.
47. Royston P, Barthel FM, Parmar MK, et al. Designs for clinical trials with time-to-event outcomes based on stopping guidelines for lack of benefit. Trials 2011; 12:81
48. Dunn DT, Glidden DV, Stirrup OT., et al . The averted infections ratio: a novel measure of effectiveness of experimental HIV pre-exposure prophylaxis agents. *Lancet HIV* 2018; 5: e329–34
49. https://www.unaids.org/en/resources/documents/2011/20110629_JC1853_GPP_Guidelines_2011%20OK
