# Supplementary material for: HIV risk perception, trust and PrEP adherence among participants in an HIV prevention trial: a qualitative longitudinal study, South Africa
Source: BMJ Open. 2025 Apr 23;15(4):e086742. doi: 10.1136/bmjopen-2024-086742 (PMC12020760; doi:10.1136/bmjopen-2024-086742)
Supplement: online supplemental file 2 [file bmjopen-15-4-s002.docx]

**In-depth Interview (IDI) guide Version 1.0**

OWENZA UCWANINGO: Qala ngokuchaza lokhu okulandelayo kumbambiqhaza;

**Interviewer**: Start by explaining the following to participant;

- Lengxoxo izothatha isikhathi esingangemizuzu enga-45 kuya kwihora elilodwa, futhi izoqoshwa.
- This interview will take about 45 minutes to an hour activity, and it will be audio recorded.
- Ngizothanda ukuzwa imibono yakho. Ayikho impendulo engalungile nelungile.
- I would like to hear your views. There are no right or wrong answers.
- Ngicela ukhululeke unikezele ngemibono yakho.
- Please feel free to share your ideas.
- Noma imuphi umbuzo ongazizwa ukhululekile ngawo, ngicela ukhulululeke ukungawuphenduli. Nakuba, ngezizathu zokuthekela ulwazi, sizothanda ukuzwa yonke into ozofisa ukuyisho. Ngakhoke, uma lokhu kungenabungozi kuwena, zama ukusitshela konke.
- Any question you feel uncomfortable with, please feel free not to answer. However, for the purpose of learning, we would like to hear everything you want to say. Therefore, if this has no harm to you, try to tell us everything.
- Amagama akho azogcinwa eyimfihlo-uma ngabe sesishicilela ingxoxo yethu, asiwasebenzisi amagama aqotho abantu. Kunalokho, sizokunikezela ngenombolo esizokwazi ukukuhlonza ngalo.
- Your names will be kept confidential-when we write our discussion, we never use people’s real names. Instead, we will allocate you with a number that we will be able to identify you with.
- Yonke imininingwane ozoyixoxa nami izogcina Phakathi kwethu sobabili (gcizelela ukuthi ngisho ithimba labasebenzi basemtholampilo angenke lazi ukuthi yini eshiwo umbambiqhaza kuwe)
- All the information you share with me will remain between the two of us (emphasise that even the clinic team will not know what the participant tells you)
- **Ngizothokoza ukuphendula yonke imibuzo engaqhamuka**
- **I will be happy to answer any questions that may come up**
- Ngicela ufake izingcingo zakho ukuba zingamemezi uma kungakulungela
- Please put your cell phones on silent if possible
- Ingabe kukulungele ukuthi siqale ingxoxo yethu?
- Is it fine with you that we start our discussion?

**QAPHELA: QOPHA USUKU NESIKHATHI SOKUQALA KWENGXOXO**

**NOTE: RECORD THE DATE AND TIME OF THE START OF THE INTERVIEW**

**Ulwazi lwangaphambilini**

**Background Information**

[Iminyaka, Inkolo, Imfundo, isimo somshado Umsebenzi]

[Age, Religion, Education, Marital status, Employment]

**Ulwazi mayelana nempilo yakho ngesikhathi samanje**

**Current personal Information**

1. Ngicela ungitshele kafushane ngawe?

Please tell me a bit about yourself?

- Izindawo oke wahlala kuzo
- Places where you have lived.
- Ubudlelwano (umndeni, ushadile/awushadile/abantwana uma bekhona Kanye nohlekisana nabo, ngesikhathi samanje nesikhathi esedlule)
- Relationship (family, married/single/children if any and partners, present and past)

**Umbuzo 1: Ulwazi kanye nokubamba iqhaza kulolucwaningo/kulokhu kuhlolwa**

**Topic 1: Knowledge and participation in the study/ trial**

1. Ngicela ungixoxele kabanzi ngalolucwaningo obambe iqhaza kulo manje?

Tell me about the study you are participating in now?

(*vumela umbambiqhaza ukuba axoxe ngemikhiqizo ayisebenzisayo, kungani futhi kanjani)*

*(Allow the participants to discuss about the products they are using, why and how)*

- *Ingabe bayazi mayelana nendlela abahlonzwe ngayo futhi kusho ukuthini kubo lokhu?*
- *Do participants know about the randomisation in the study and what this means to them?*
- *Ingabe bayazi mayelana nephilisi Kanye nemijovo yokuvikela (PrEP) kulolucwaningo?*
- *Do they know about the pill and HIV prevention injection (PrEP) in the study?*

1. Kungani ukhethe ukubamba iqhaza kulolucwaningo?

Why did you choose to participate in the study?

*(landelela mayelana nokugqugquzeleka, umthelela wontanga, isipiliyoni kucwaningo olwedlule, igciwane lesandulela ngculazi noma ingculazi uqobo noma izifo ezithelelana ngokocansi)*

*(Probe about motivation, peer influence, previous research experience, HIV/AIDS/STIs)*

**Umbuzo 2: Ulwazi/ ukusentsenziswa kanye nokwamukeleka kuka PrEP**

**Topic 2: Knowledge/ Use and acceptability of PrEP**

1. Yini oyaziyo ngo-PrEP?

What do you know about PrEP?

(ARVs, athathwa umuntu ongathelelekile, izindlela zokuvikela)

(ARVSs, taken by an uninfected person, prevention methods).

2. Ucabangani ngephilisi olithathayo kulolucwaningo?

What do you think about the pill that you are taking in this study?

*(Thola kabanzi ngendlela elibukeka ngayo, indlela elinambitheka ngayo, usayizi walo, okuthandekayo nokungathandeki, ukwesaba, ingabe bayakwazi yini ukuhlukanisa uma ngabe baku TAF noma TDF)*

*(Probe more about its appearance, taste, size, likes and dislikes, fears, are they able to tell whether they are on TAF or TDF)*

1. Yini oyicabangayo ngokusebenzisa ama- ARVs kodwa ungenalo igciwane lesandulela ngculazi ( imizwa, izinsolo, izinkinga zengcindezi, imiphumela engemihle, njalo njalo)

What do you think about taking ARVs, but you are HIV negative (feelings; regrets, stress problems, side effects, etc)

1. Abantu kuyenzeka ukuthi bekhohlwe ukudla amaphilisi abo sikhathini esiningi. Ingabe zikhona izikhathi lapho ungawadli khona amaphilisi akho njengoba uyaleziwe? (thola kabanzi ngokungawadli amaphilisi)

People often forget to take pills they are supposed to take on a regular basis. Are there times you have failed to take the pill as instructed? (probe more about medication non-adherence)

1. Yini ekwenze kwaba lula noma kwaba nzima ukudla amaphilisi? *(izingqinamba kanye nabaluleki)*

What has made it easy/difficult to take the pills? *(barriers & facilitators)*

1. Ingabe iphilisi liyavikela igciwane sesandulela ngculazi noma ingculazi kanye nezifo ezithathelana ngokocansi? Izizathu? (*thola kabanzi ukuthi ucabangani ngokuthathwa kwephilisi ekubeni bathola umjovo baphinde futhi bagqugquzelwe ngokusebanzisa ikhondomu)*

Does the pill protect against HIV/AIDS/STIs? *Reasons? (Probe more on what they think about taking the pill, but they were vaccinated and encouraged to use condoms)*

**Umbuzo 3: Ukusabela kwabanye abantu (abahlekisana nabo noma abantu abasondelene nabo(imindeni, abasebenza nabo, ontanga, njalo njalo)**

**Topic 3: Other people’s reactions (partners/ people around them (family, workmates, peers, etc)**

1. Ingabe kukhona omunye umuntu owaziyo ukuthi ubambe iqhaza kulolucwaningo? Ubani?

Is there anyone else who knows that you are participating in this study? Who?

1. Ingabe abantu ohlekisana nabo/ abantu osondelene nabo (umndeni, osebenza nabo, ontanga, njalo njalo) bacabangani ngokungenelela kwakho kulolucwaningo? (*buza ngezinto ezinhle ezenzekalile, izingqinamba, izikhalazo, kanye nokwesaba okuvela kumuntu ohlekisana naye/* kubantu osondelene nabo (umndeni, osebenza nabo, ontanga, njalo njalo)

What do your partners/ people around you (family, workmates, peers, etc) think about your enrolment in this study? (*Ask about good things that happened, challenges, concerns, and fears from partner*/ people around them (family, workmates, peers, etc)

1. Umuntu ohlekisana naye/ abantu osondelene nabo (umndeni, osebenza nabo, ontanga, njalo njalo)bakweseke kanjani ukuba ingxenye yalolucwaningo kwakho?

How did your partner/ people around you (family, workmates, peers, etc) support your participation in the trial?

**Umbuzo 4: Izingcuphe ezingaba khona**

**Topic 4:** **Risk Perception**

1. **Kufanele kubuzwe kwinhlolovo yokuqala yalolucwaningo**: ingabe uzizwa usengcupheni yokutheleleka ngegciwane lesandulela ngculazi noma ingculazi? Chaza impendulo yakho.

**To be asked at first interview in the study:** Do you feel at risk of contracting HIV/AIDS? Explain your answer.

1. **Kumele ubuzwe kwinhlolovo elandelayo:** uzibona lkanjani izingcuphe manje, uma uqhathanisa ngenkathi uqala kulolucwaningo?

**To be asked at follow up visits:** How is your perception of risk now, compared to when you started in this study?

**Umbuzo 5: ukwamkeleka kwenqubomgomo yocwaningo**

**Topic 5: Acceptability of study procedures**

1. Sithini isipiliyoni sakho ngokusebenzisa idayari kulelicwaningo? (*kulula, kunzima, okunye ongakusho)*

What are your experiences with the diary used during the study? *(Easy, difficult, any other comments)*

1. Uthini imicabanga yakho mayelana nezinye izinqubo ezenziwayo (ukuthathwa kwamasampuli, imijovo nokuxilongwa)?

What were your thoughts about the other study procedures (taking of samples, vaccination, and examinations)?

1. Buza mayelana nokuzithwala; kungani ucabanga ukuthi wena/uphathina wakho nigqugquzelwe ukuba ningazitholi nizithwele niseyingxenye kulolucwaningo?

Ask about pregnancy; why do you think you/your partner are encouraged not to get pregnant while participating in this study?

- Uzizwa kanjani ngalemfuneko yokungenelela kulolucwaningo (thola kabanzi nmuzwa ongemuhle ongavela kuphathina kamabambiqhaza, izingxoxo zokuhlehliswa kokuzithwala Kanye nezinye izimo)
- How do you feel about this requirement for participation in the study (probe more about feelings of resentment from partner, discussions to postpone pregnancy and other issues)?

1. Manje, ake sikhulume ngokubamba kwakho iqhaza kulolucwaningo

Now, let us talk about your participation in the study.

- Iziphi izinto ezikwenze ukuba uqhubeke ubambe iqhaza kulolucwaningo?
- What factors made you to continue participating in the study?
- Kungaba yini “leyonto” engakwenza udikile ukubamba iqhaza kulolucwaningo?
- What could be that ‘thing’ that may cause you to drop out of the study?

**Owenza ucwaningo:** gcina ngokugqugquzela umbambiqhaza ukuba abuze imibuzo uma ngabe enayo; phendula leyo mibuzo ozokwazi ukuyiphendula, futhi yidlulisele phambili ukuthola ulwazi olwanele ngayo uma ungakhoni ukuyiphendula.

**Interviewer:** End by encouraging the participant to ask any questions they may have; answer those you are able to and refer them for clarification of those you are unable to answer.

Manje, bonga umbambiqhaza bese uvala ingxoxo.

Now, thank the participant and close the discussion.
