# Supplementary material for: HIV risk perception, trust and PrEP adherence among participants in an HIV prevention trial: a qualitative longitudinal study, South Africa
Source: BMJ Open. 2025 Apr 23;15(4):e086742. doi: 10.1136/bmjopen-2024-086742 (PMC12020760; doi:10.1136/bmjopen-2024-086742)
Supplement: Supplementary file 3 [file bmjopen-2024-086742.reviewer_comments.pdf]

PEER REVIEW HISTORY

BMJ Open publishes all reviews undertaken for accepted manuscripts. Reviewers are asked to complete a checklist review form and are provided with free text boxes to elaborate on their assessment. These free text comments are reproduced below.

ARTICLE DETAILS

Title (Provisional)

HIV risk perception, trust and PrEP adherence among participants in an HIV prevention trial: a qualitative longitudinal study, South Africa.

Authors

Chimukuche, Rujeko Samanthia; Shandu, Londiwe; Zulu, Silindile; Khanyile, Phindile; Singh, Nishanta; Gaffoor, Zakir; Kawuma, Rachel; McCormack, Sheena; Seeley, Janet; PrEPVACC, STUDY GROUP

VERSION 1 - REVIEW

**Reviewer**	**1**
**Name**	**Atukunda, Esther**
**Affiliation**	**Mbarara University of Science and Technology**
**Date**	**23-Apr-2024**
**COI**	**ok**

Line 18-25 is confusing, re-write.

Please correct inconsistencies in referencing (line 48/49). Correct many typos that make reading difficult, eg in results section, “Four participants stated there were single, and 26 indicated there were involved in a relationship”.

Please provide a reference for the first part of this assertion, “This perception therefore correlates with the willingness to use PrEP, which is key in PrEP uptake and adherence (11)”

The TCC framework details could be integrated into the methods section for more clarity..

Please provide details on the eligibility criteria for this particular substudy, how participants were identified, consented and interviewed. Where they were interviewed and at what time during the main study.

Provide the ethics statement, and details of the trial/ trial registration

Results/findings are presented as discussion, e.g…”HIV risk perception plays a pivotal role in shaping individuals' behaviours and decisions concerning their sexual health….Confidence is defined as one's self-assurance and belief in their own abilities or in the abilities of their team or organization (15). The TCC framework states that individuals develop confidence and trust in institutions when they receive accurate and reliable information about preventative measures from trustworthy sources(15). In this analysis….”

The results presented under Trust, confidence and cooperation are widely disconnected from the quotes, and need to be further synthesized in the narrative that proceed the verbatim for more clarity

The discussion could be hinged on the presented key findings in the results section

Some references are outdated

**Reviewer**	**2**
**Name**	**Maggioni, Martina A.**
**Affiliation**	**Charite - Universitatsmedizin Berlin**
**Date**	**31-Oct-2024**
**COI**	**no**

Dear Autors,

find here below minor comments:

1-The Acronyms PrEP and TCC should be clearly disclosed in the Abstract.

2- Lines 11-16: please provide a justification for the sample size you defined

3- Please provide information about the Ethic Committee Approval for this study

Major comments are summarised as below:

Title: HIV risk perception, trust and PrEP adherence among participants in an HIV prevention trial: a qualitative longitudinal study, South Africa.

Summary of the paper:

This study utilized **in-depth longitudinal interviews** (at three time points) with **purposive sampling** to explore changes in participants' risk perception and adherence to PrEP over time, and to understand how continued engagement/cooperation with the **PrEPVacc vaccine clinical trial** (in Durban, South Africa) was influenced by trust and confidence, in accordance with the **TCC framework**.

Behaviors indicative of cooperation/engagement with the clinical trial included: 1. attending trial visits; 2. adhering to study procedures; 3. participating in counseling sessions; 4. consistently taking PrEP; and 5. committing to regular HIV testing. The key drivers of engagement in the PrEP trial comprised perceived risk stemming from a lack of interpersonal trust (in sexual partners), trust in the information provided by the trial team, confidence in the eSicacy of PrEP, and an implied trust in the trial team to maintain a consistent supply of PrEP medications.

Strengths of the Paper:

1. **Longitudinal Design**: The use of a longitudinal approach allows for the collection of data over time, oSering insights into changes in participants' HIV risk perception and PrEP adherence. This design helps identify patterns and key shifts in behaviour, which are crucial for understanding how trust, confidence, and intervention adherence,

develops throughout the course of a clinical trial, and potentially in real life settings

2. **Use of TCC Framework**: The application of the Trust, Confidence, and Cooperation (TCC) framework is a well-chosen model that eSectively explores how participants' trust in the clinical trial influences their risk perception and health behaviours. This theoretical grounding strengthens the analysis and helps tie participants' experiences to broader themes of trust and health intervention acceptance.

3. **Diverse Sample**: The study sample includes participants from diSerent age-groups, genders and socio-economic backgrounds, which provides a more comprehensive view of the experiences of people at risk of HIV in the context of a clinical trial. This variation helps ensure that the findings are not limited to a narrow population.

Areas for Improvement:

1. **Definitions:** While the paper discusses concepts related to trust, confidence, and cooperation, several terms have not been clearly defined, even though their meanings are implicit throughout the text. Clarifying these definitions would enhance the reader's understanding of how these concepts interact and influence participants' behaviours within the study. Based on the paper here are the relevant terms:

    a) **Trust:** Belief in the reliability and integrity of another party (an individual or institution), influencing willingness to cooperate/engage based on positive expectations. In this paper there are two forms:

        a) Interpersonal trust or mistrust of sexual partners: this is clear in the text and is the focus

        b) Institutional trust (trust in the clinical trial team): this has not been made explicit in the definition and is only presented in passing as trust in the competence and integrity of the clinical trial team to deliver accurate information)

    b) **Confidence**: Belief in:

        a) one’s own abilities to navigate negative consequences during cooperation (e.g. engaging in clinical trial)

        b) the eSectiveness of an intervention (like PrEP), and

        c) the competence of the institution or trial team

            - the first component - (a)- has been defined explicitly in the text but not applied in the analysis. The other two have not been included in the definition but have been applied in the analysis

    c) **Cooperation:** The act of working together towards common goals, characterized by mutual support and active participation. This has been defined explicitly in the paper . Various parts of the paper mention behaviours indicative of cooperation with the study team including: 1. attendance of trial visits; 2. adhering to study procedures; 3. attending counselling sessions; 4. adherence to PreP; 5. committing to regular HIV testing

2. Relevance of quotes:

a) Regarding trust, the paper has provided relevant quotes related to mistrust in

sexual partner as driving the desire to take PreP. These include mistrust in partner because of: 1. not knowing their status while having condomless sex with them; 2. Knowing that they engage in risky behaviour- multiple partners, condomless sex. However, there are no relevant quotes and adequate discussion of institutional trust, focus is on interpersonal trust

b) In terms of confidence there are no quotes or discussion of views related to self-confidence. The focus is on confidence in eSicacy of PreP and confidence in trial team- which however has been reported under cooperation section. For example , quotes **in lines 29-39 and 41-46 of page 9** are about behaviour change as a result of institutional trust .

c) As for cooperation, hardly any of the quotes presented in the results are about evidence showing engagement with the study i.e. exhibiting cooperative behaviours. For example, quote **in lines 26-33 of page 12** implies that participants were encouraged or required to change their sexual practices in light of their involvement in the study...is this a study requirement they were expected to adhere to? Also, the reaction quoted in lines **43-53 of page 13** indicates ***a lack of confidence*** in the ability of public facilities to meet their ongoing health needs. This could have been presented in the "confidence" section of the paper

3. **Discussion on Broader Applicability of Findings**: While the paper highlights that the trial provided better access to PrEP and healthcare support than typical real-life settings, it also acknowledges participants' concerns about limited access to PrEP after the trial, particularly in public clinics. However, the discussion lacks detail on other real- life barriers, such as logistical and transport challenges, that could impact adherence outside of the clinical environment. Expanding on these aspects and suggesting future research directions could enhance the applicability of the findings to broader populations.

4. **Potential oversimplification:** Also, while the TCC model emphasizes **cooperation**, it assumes that once trust and confidence are established, cooperation will naturally follow. However, cooperation might be influenced by **other dynamics**, such as personal priorities, competing interests, social norms, or barriers to access (e.g., logistical challenges, cost of the intervention), which may not be adequately captured in the TCC model. It would be useful to add this to the discussion. The study lacks an explicit discussion on how trust in the trial was historically established in the specific setting. While it emphasizes the role of trust in the clinical trial staS and the information provided, it does not explore the **historical context** or past experiences that may have shaped participants' trust in the trial team. Have the trial site engaged with the community where the participants come from in the past with positive outcomes?

5. **Additional Exploration of Non-Adherers**: The paper focuses on the experiences of those who continued to adhere to PrEP. Including more details about participants who dropped out or did not adhere fully could shed light on the barriers that remain even within the trial setting.

6. **Clearer Description of Data Saturation**: While the paper mentions that some level of saturation was reached, it would benefit from a more explicit explanation of how data saturation was assessed and determined. This would further support the validity of the findings.

VERSION 1 - AUTHOR RESPONSE

Reviewer: 1

1) **Line 18-25 is confusing, re-write.**
Response: We have edited the text. Lines 90-95

HIV risk perception is related to the level of trust a person has in the behaviour of their sexual partner, this influences whether someone believes they need to take preventive measures to maintain their HIV negative status. This perception therefore correlates with the willingness to use PrEP, which is key in PrEP uptake and adherence.

2) **Please correct inconsistencies in referencing (line 48/49). Correct many typos that make reading difficult, eg in results section, "Four participants stated there were single, and 26 indicated there were involved in a relationship".**
Response: We have edited the sentences.
Four participants stated that they were single with no partners, and 26 indicated that they were in a relationship. Line 199-200
3) **Please provide a reference for the first part of this assertion, "This perception therefore correlates with the willingness to use PrEP, which is key in PrEP uptake and adherence (11)"**
Response: We have edited the sentence with one reference.
This perception therefore correlates with the willingness to use PrEP which is key in PrEP uptake and adherence (11). Line 93-95
4) **The TCC framework details could be integrated into the methods section for more clarity…**
Response: The TCC framework was used to create the coding framework and analyse the data. We have included this in the analysis process text:
Data analysis was done manually, led by the first author (RSC), and the research assistants (LS, PM, and SZ) who collected the data. The coding framework was developed by RSC using the TCC model and from the data which the entire team reviewed and used to identify emerging codes. The main codes were reviewed, discussed and agreed upon by consensus for validity, and similar codes were merged to avoid duplication. For this paper, major themes were identified using the theoretical framework: trust, confidence and cooperation. Thereafter, for this analysis a matrix coding framework was developed in MS Excel on to which coded data were manually inserted by pasting information and illustrative quotes from the interviews against the matching themes. Line 186-194.
5) **Please provide details on the eligibility criteria for this particular substudy, how participants were identified, consented and interviewed. Where they were interviewed and at what time during the main study.**
Response: We have included a detailed description of the recruitment process.
Individuals who were HIV negative (18 to 40 years) and considered to be at high risk of HIV acquisition as per the baseline risk assessment criteria, were recruited to join the PrEPVacc study. Every participant was encouraged to take PrEP once they enrolled in the study. Thirty participants, nine percent of the total enrolled in Durban were

purposively selected to be representative of those randomised to TAF/ FTC (Descovy) or TDF/FTC (Truvada), age group (18-40 years), gender and socio-economic characteristics. The type of oral PrEP given was known to participants within the clinical trial (participants were not blinded to the type of PrEP). Line 151-159.

6) **Provide the ethics statement, and details of the trial/ trial registration**
Response: We thank you for the comment. We have edited and included the ethics statement and detail in lines 140-141:
Ethical approval for the study was obtained from the South Africa Human Research Ethics Committee: Protocol ID EC020-11/2019.

7) Results/findings are presented as discussion, e.g…"HIV risk perception plays a pivotal role in shaping individuals' behaviours and decisions concerning their sexual health….Confidence is defined as one's self-assurance and belief in their own abilities or in the abilities of their team or organization (15). The TCC framework states that individuals develop confidence and trust in institutions when they receive accurate and reliable information about preventative measures from trustworthy sources(15). In this analysis…
Response: We have edited the results section.
Our results have shown that participants' trust in the clinical trial was shaped by their past experiences of engaging in high-risk behaviours with multiple partners. Lines 207-210, Lines 266-269.

8) **The results presented under Trust, confidence and cooperation are widely disconnected from the quotes, and need to be further synthesized in the narrative that proceed the verbatim for more clarity**
Response: We have edited the results section
Lines 253-255
Client-centred counselling at every visit helped build trust and reinforce adherence and motivation. The participants were motivated to keep coming for their trial visits and to recognise the HIV risk they had previously exposed themselves to.
Lines 330-331
Our results indicate that trust put in the health services provided by the clinical trial encouraged cooperation among participants, and instilled confidence in the support provided.

9) **The discussion could be hinged on the presented key findings in the results section**

Response: We have added more text to the discussion
Lines 422-426: *Trust was also cultivated by the clinical trial community and public engagement teams during recruitment and during the ongoing trial who engaged with the communities, sharing HIV prevention information on the services provided by the clinical trial. Community engagement teams offered pre-screening workshops prior to trial participation. In addition, the clinical trial site was known in the community as a place where several studies had been conducted.*

Lines 430-443: *Our results highlighted concern over post-trial access to PrEP. This has been highlighted in other studies with participants stating several health system barriers such as long queues at public health facilities and transport costs associated with travel to clinics. A study conducted among adolescent girls in Kenya and South Africa showed that barriers to oral PrEP included being unable to collect PrEP refills due to school commitments, and lacking transport to fetch PrEP from clinics. Differentiated models that include mobile and community-based platforms for oral PrEP delivery can be considered as these might address facility-based barriers to oral PrEP access.*
Barriers to uptake of PrEP have been recorded in the study including people reporting side effects from PrEP leading to discontinued use. Interpersonal barriers due to, for example, resistance from family members to PrEP uptake, were also reported, which corroborates the finds from other studies (31). Such barriers persisted in the clinical trial despite supportive services such as counselling and constant health monitoring.

10) Some references are outdated

Response: We have included new references in lines 435, 437, 442 and 616-626

Beesham I, Milford C, Smit J, Joseph Davey DL, Baeten JM, Heffron R, et al. Post-trial access to and use of pre-exposure prophylaxis in Durban, South Africa. BMC Public Health. 2023;23(1):1210.

Rousseau E, Katz AWK, O'Rourke S, Bekker LG, Delany-Moretlwe S, Bukusi E, et al. Adolescent girls and young women's PrEP-user journey during an implementation science study in South Africa and Kenya. PLoS One. 2021;16(10):e0258542.

Rousseau E, Bekker LG, Julies RF, Celum C, Morton J, Johnson R, et al. A community-based mobile clinic model delivering PrEP for HIV prevention to adolescent girls and young women in Cape Town, South Africa. BMC Health Serv Res. 2021;21(1):888.

Stoebenau K, Muchanga G, Ahmad SS-O, Bwalya C, Mwale M, Toussaint S, et al. Barriers and facilitators to uptake and persistence on prep among key populations in Southern Province, Zambia: a thematic analysis. BMC Public Health. 2024;24(1):1617.

Reviewer: 2

11) The Acronyms PrEP and TCC should be clearly disclosed in the Abstract.

Response: We have added the full text version to the abstract

In this qualitative analysis, our aim was to explore risk perceptions and understand how individuals assess and respond to HIV risks, as well as their uptake and adherence to oral pre-exposure prophylaxis (PrEP). Lines 43-44

Data analysis was conducted using the Trust, Confidence and Cooperation (TCC) framework that included constructs of trust, confidence and cooperation. Line 49-50

12) **Lines 11-16: please provide a justification for the sample size you defined**
Our sample for the qualitative sub-study was 9% of the total clinical trial sample as described in lines 151-155.

*Thirty participants, nine percent of the total enrolled in Durban were purposively selected to be representative of those randomised to TAF/ FTC (Descovy) or TDF/FTC (Truvada), age group (18-40 years), gender and socio-economic characteristics***.** *The sample size was based on our experience and that of others, to enable us to gather sufficient data on our areas of focus.*

13) **Please provide information about the Ethic Committee Approval for this study**
Response: We have added ethics the statement in lines 140-141

Ethical approval for the study was obtained from the South Africa Human Research Ethics Committee: Protocol ID EC020-11/2019.

Areas for Improvement:

14) **Definitions:** While the paper discusses concepts related to trust, confidence, and cooperation, several terms have not been clearly defined, even though their meanings are implicit throughout the text. Clarifying these definitions would enhance the reader's understanding of how these concepts interact and influence participants' behaviours within the study. Based on the paper here are the relevant terms:
    a) **Trust:** Belief in the reliability and integrity of another party (an individual or institution), influencing willingness to cooperate/engage based on positive expectations. In this paper there are two forms:

Interpersonal trust or mistrust of sexual partners: this is clear in the text and is the focus

Institutional trust (trust in the clinical trial team): this has not been made explicit in the definition and is only presented in passing as trust in the competence and integrity of the clinical trial team to deliver accurate information)

Response: Thank you for this comment. We have included the definitions of trust in the text to read: *Trust is the willingness to make oneself vulnerable believing in the integrity of another whilst expecting beneficial outcomes(15). It can be interpersonal trust or lack thereof between two individuals or institutional trust where individuals place confidence in an institution (16)* in lines 110-113.

15) Confidence: Belief in:
    a) one's own abilities to navigate negative consequences during cooperation (e.g. engaging in clinical trial)
    b) the effectiveness of an intervention (like PrEP), and
    c) the competence of the institution or trial team

the first component - (a)- has been defined explicitly in the text but not applied in the analysis. The other two have not been included in the definition but have been applied in the analysis

Response: We have edited the text to include the concept of confidence in the analysis to read in line 240-255:

The participants' confidence in the health services that were provided through the clinical trial and the provision of PrEP as an intervention significantly influenced their perception of low HIV risk.

"It was because, every time I always check my blood, but I thought I will get to the point where I get scared to. What I like here is that…what actually brought me here was I heard that there is PrEP, the pills to prevent HIV infection, that is what motivated me to come here. Also, that we check blood whenever we come here... Like I have said, there are a lot of things here. They can show us things, even the counsellors here they know how to talk with you.." Female 28 years (IDI 3).

The client centred counselling at every visit helped build trust and reinforce adherence and motivation. The participants were motivated to keep coming and to recognise the HIV risk they had previously exposed themselves to.

16) Cooperation: The act of working together towards common goals, characterized by mutual support and active participation. This has been defined explicitly in the paper . Various parts of the paper mention behaviours indicative of cooperation with the study team including: 1. attendance of trial visits; 2.adhering to study procedures; 3. attending counselling sessions; 4. Adherence to PreP; 5. committing to regular HIV testing

Response: We thank you for the comment, the definition of cooperation is in line 334-335

Cooperation involves the act of working together with others towards common goals or objectives.

Relevance of quotes:

a) **Regarding trust, the paper has provided relevant quotes related to mistrust in sexual partner as driving the desire to take PreP. These include mistrust in partner because of: 1. not knowing their status while having condomless sex with them; 2. Knowing that they engage in risky behaviour- multiple partners, condomless sex. However, there are no relevant quotes and adequate discussion of institutional trust, focus is on interpersonal trust**

Response: We have added quotes on institutional trust in lines 244-255

The participants' confidence in the health services that were provided at the clinical trial and PrEP as an intervention significantly influenced their perception of low HIV risk.

"It was because, every time I always check my blood, but I thought I will get to the point where I get scared to. What I like here is that…what actually brought me here was I heard that there is PrEP, the pills to prevent HIV infection, that is what motivated me to come here. Also, that we check blood whenever we come here... Like I have said, there are a lot of things here. They can show us things, even the counsellors here they know how to talk with you..." Female 28 years (IDI 3).

The client centred counselling at every visit helped build trust and reinforce adherence and motivation. The participants were motivated to keep coming and to recognise the HIV risk they had previously exposed themselves to.

17) **In terms of confidence there are no quotes or discussion of views related to self-confidence.**
The focus is on confidence in efficacy of PreP and confidence in trial team- which however has been reported under cooperation section. For example , quotes in lines 29-39 and 41-46 of page 9 are about behaviour change as a result of institutional trust .
Response: Thank you. We have edited the text and added quotes on self-confidence.
A growing self-confidence in the knowledge of their own HIV status was also noticed amongst the participants in the clinical trial.
"Er, first of all what makes me to continue with the study is that I will always know that I am not sick. I am always here, you see that thing, ja. That is what I like about here also the treatment here there are no problems, they all right." Male 31 years (IDI 2)
The belief and trust in the competence of the clinical trial team built the participants confidence. Lines 290-299.

18) **As for cooperation, hardly any of the quotes presented in the results are about evidence showing engagement with the study i.e. exhibiting cooperative behaviours. For example, quote in lines 26-33 of page 12 implies that participants were encouraged or required to change their sexual practices in light of their involvement in the study...is this a study requirement they were expected to adhere to?**
Response: We thank you for this comment. The quote from the participant was explaining how they have shown commitment to the study by changing their behaviour adhering to preventative measures as advised by the clinical trial staff as explained in lines 335-343.
Our results also showed participants cooperated with the trial by taking PrEP, attending counselling sessions and adhering to the study procedures. They recognized the potential seriousness of HIV transmission and the implications of not knowing one's HIV status. Consequently, as the study progressed participants adjusted their behaviour by emphasizing moral responsibility and cooperation.
"Currently because I am part of this research study, I have to conduct myself in a good moral manner because at the end of the day, you cannot know or tell any of the persons you meet their HIV status and you cannot convince them either for them to know their HIV status by having their test done." Female, 30 years (IDI 2)

19) Also, the reaction quoted in lines 43-53 of page 13 indicates a lack of confidence in the ability of public facilities to meet their ongoing health needs. This could have been presented in the "confidence" section of the paper
Response: We have edited the text and added this to the confidence section in lines 320-331
Lack of confidence in the ability of public health facilities to provide PrEP was highlighted by participants. Participants reflected on the importance of access to PrEP as important in adherence. They expressed concern at the prospect of the study ending and the potential unavailability of and access to PrEP through public clinics.
"I do not want to lie; I would really feel sad if the study were to come to an end and there is no other way of continuing with PrEP because when you ask, most of the public

clinics do not offer PrEP and even though they have done the training for PrEP but they haven't started to receive it or to distribute it and so it will really be difficult for us who are really relying on it and as we have gotten used to it." Female, 30 years (IDI 1)Our results indicate that trust put in the health services provided by the clinical trial encouraged cooperation among participants, and instilled confidence in the support provided.

20) **Discussion on Broader Applicability of Findings: While the paper highlights that the trial provided better access to PrEP and healthcare support than typical real-life settings, it also acknowledges participants' concerns about limited access to PrEP after the trial, particularly in public clinics. However, the discussion lacks detail on other real life barriers, such as logistical and transport challenges, that could impact adherence outside of the clinical environment. Expanding on these aspects and suggesting future research directions could enhance the applicability of the findings to broader populations.**

    Response: We thank you for the comment. We have added some text in Lines 430-437:

    Our results highlighted concern over post-trial access to PrEP. This has been highlighted in other studies with participants stating several health system barriers such as long queues at public health facilities and transport costs associated with travel to the clinics. A study conducted among adolescent girls in Kenya and South Africa showed that barriers to oral PrEP included being unable to collect PrEP refills due to school commitments, and lacking transport to fetch PrEP from clinics. Differentiated models that include mobile and community-based platforms for oral PrEP delivery can be considered as these might address facility-based barriers to oral PrEP access.

21) **Potential oversimplification: Also, while the TCC model emphasizes cooperation, it assumes that once trust and confidence are established, cooperation will naturally follow. However, cooperation might be influenced by other dynamics, such as personal priorities, competing interests, social norms, or barriers to access (e.g., logistical challenges, cost of the intervention), which may not be adequately captured in the TCC model. It would be useful to add this to the discussion. The study lacks an explicit discussion on how trust in the trial was historically established in the specific setting. While it emphasizes the role of trust in the clinical trial staS and the information provided, it does not explore the historical context or past experiences that may have shaped participants' trust in the trial team. Have the trial site engaged with the community where the participants come from in the past with positive outcomes?**

    Response: *Trust was also cultivated by the clinical trial community and public engagement teams during recruitment and during the ongoing trial who engaged with the communities sharing HIV prevention information on the services provided by the clinical trial. Community engagement teams offered pre-screening workshops prior to trial participation. In addition, the clinical trial site was known in the community as a place where several studies had been conducted.* Lines 422-426.

22) Additional Exploration of Non-Adherers: The paper focuses on the experiences of those who continued to adhere to PrEP. Including more details about participants who dropped out or did not adhere fully could shed light on the barriers that remain even within the trial setting.

Response: We thank you for the comment and we have added this text in lines 370-381

However in the study there were people who discontinued PrEP who said that they had to stop taking PrEP because of the side effects they experienced.

'Whenever I took PrEP tablets, if I didn't take them, I won't have anything, but once I take it at 20:00, I would know that I will not sleep... Ey, I took them for the first three days, then maybe two weeks passed by. I tried them again, wanting to see the actual cause of my sickness. As I have had both the tablets and the injection when I started, I wasn't sure which one was making me sick. So, I continued taking the tablets for two days for that following two weeks. Still, the something happened, that's when I stopped taking them, I never tried them again." Female 29 years (IDI2)

and Lines 439-443 :

Barriers to uptake of PrEP have been recorded in the study including people reporting side effects from PrEP leading to discontinued use. Interpersonal barriers due to, for example, resistance from family members to PrEP uptake, were also reported, which corroborates the findings from other studies (31). Such barriers persisted in the clinical trial despite supportive services such as counselling and constant health monitoring.

23) Clearer Description of Data Saturation: While the paper mentions that some level of saturation was reached, it would benefit from a more explicit explanation of how data saturation was assessed and determined. This would further support the validity of the findings.

Response: We thank you for the comment. Our sample for the qualitative sub-study was 9% of the total clinical trial sample as described in lines 151-156.

Thirty participants, nine percent of the total enrolled in Durban were purposively selected to be representative of those randomised to TAF/ FTC (Descovy) or TDF/FTC (Truvada), age group (18-40 years), gender and socio-economic characteristics. ***.*** *The sample size was based on our experience and that of others, to enable us to gather sufficient data on our areas of focus. The type of oral PrEP given was known to participants within the clinical (participants were not blinded to the type of PrEP).*

VERSION 2 - REVIEW

**Reviewer**	**2**
**Name**	**Maggioni, Martina A.**
**Affiliation**	**Charite - Universitatsmedizin Berlin**
**Date**	**13-Jan-2025**

COI

The authors addressed very well all my suggestions and I think the manuscript has been extremely improved and it should be accepted for publication in his present form.
